# Modified Atmosphere Does Not Reduce the Efficacy of Phytosanitary Irradiation Doses Recommended for Tephritid Fruit Flies

**DOI:** 10.3390/insects11060371

**Published:** 2020-06-15

**Authors:** Vanessa S. Dias, Guy J. Hallman, Olga Y. Martínez-Barrera, Nick V. Hurtado, Amanda A. S. Cardoso, Andrew G. Parker, Luis A. Caravantes, Camilo Rivera, Alexandre S. Araújo, Florence Maxwell, Carlos E. Cáceres-Barrios, Marc J. B. Vreysen, Scott W. Myers

**Affiliations:** 1 Insect Pest Control Laboratory, Joint FAO/IAEA Division of Nuclear Techniques in Food and Agriculture, IAEA, Wagramer Strasse 5, 1400 Vienna, Austria; oyamaba@gmail.com (O.Y.M.-B.); nickvahurt312013@gmail.com (N.V.H.); amandaascardoso@gmail.com (A.A.S.C.); a.g.parker@protonmail.com (A.G.P.); lax.caravantes@gmail.com (L.A.C.); cam1lo.11194@gmail.com (C.R.); alexandressa2@live.com (A.S.A.); F.Maxwell@iaea.org (F.M.); C.E.Caceres-Barrios@iaea.org (C.E.C.-B.); M.Vreysen@iaea.org (M.J.B.V.); 2 Phytosanitation, 3917 Estancia Drive, Oceanside, CA 92058, USA; 3 USDA, APHIS, PPQ, Science and Technology, Otis Laboratory 1398 W. Truck Rd., Buzzards Bay, MA 02542, USA; scott.w.myers@usda.gov

**Keywords:** hypoxia, normoxia, phytosanitation, oxygen effect, radiotolerance, radiation sensitivity, radioprotection, radioresistance

## Abstract

Phytosanitary irradiation (PI) has been successfully used to disinfest fresh commodities and facilitate international agricultural trade. Critical aspects that may reduce PI efficacy must be considered to ensure the consistency and effectiveness of approved treatment schedules. One factor that can potentially reduce PI efficacy is irradiation under low oxygen conditions. This factor is particularly important because storage and packaging of horticultural commodities under low oxygen levels constitute practices widely used to preserve their quality and extend their shelf life. Hence, international organizations and regulatory agencies have considered the uncertainties regarding the efficacy of PI doses for insects infesting fresh commodities stored under low oxygen levels as a rationale for restricting PI application under modified atmosphere. Our research examines the extent to which low oxygen treatments can reduce the efficacy of phytosanitary irradiation for tephritids naturally infesting fruits. The effects of normoxia (21% O_2_), hypoxia (~5% O_2_), and severe hypoxia (< 0.5% O_2_) on radiation sensitivity of third instars of *Anastrepha fraterculus* (sensu lato), *A. ludens* (Loew), *Bactrocera dorsalis* (Hendel), and *Ceratitis capitata* (Wiedemann) were evaluated and compared at several gamma radiation doses. Our findings suggest that, compared to normoxia, hypoxic and severe-hypoxic conditioning before and during irradiation can increase adult emergence and contribute to advancement of larval development of tephritid fruit flies only at low radiation doses that are not used as phytosanitary treatments. With phytosanitary irradiation doses approved internationally for several tephritids, low oxygen treatments applied before and during irradiation did not increase the emergence rates of any fruit fly species evaluated, and all treated insects died as coarctate larvae. Thus, the findings of our research support a re-evaluation of restrictions related to phytosanitary irradiation application under modified atmospheres targeting tephritid fruit flies.

## 1. Introduction

Ionizing radiation is a relatively recent phytosanitary treatment that is increasing in use [1]. One factor that has the potential to reduce the efficacy of phytosanitary irradiation (PI) is reduced oxygen levels before and during radiation treatments [2,3]. Oxygen reduction may be caused intentionally to preserve commodity quality [4,5] or occur when commodities respire after packaging. International organizations and regulatory agencies have responded to this risk by restricting the application of PI in situations where commodities are maintained under modified atmospheres with low oxygen levels [6,7]. The International Plant Protection Convention (IPPC) encourages the restriction of PI treatments targeting insect pests for commodities stored in modified atmospheres [8,9,10,11,12,13], with the exception of *Rhagoletis pomonella* (Walsh) [14] and *Grapholita molesta* (Busck) [15]. The United States Department of Agriculture Animal and Plant Health Inspection Service (USDA APHIS) imposes partial restrictions on radiation treatments in modified atmospheres and recently reduced the oxygen limits for commodities that are in packaging or other conditions from 18 to 10% [7].

The concerns addressed by international plant protection and regulatory organizations regarding the application of PI under modified atmosphere are reasonable, considering the mechanisms underlying radiation sensitivity in several organisms. Insects pre-conditioned and irradiated under hypoxic or anoxic conditions may be protected against radiation damage by, among others, the reduction of reactive oxygen species (ROS) formation [16,17,18] and increased enzymatic antioxidant protection [19,20,21]. Tephritids, drosophilids, lepidopterans, and coleopterans irradiated with nonlethal doses of radiation, often below recommended phytosanitary irradiation doses, under reduced oxygen levels are less radiation sensitive than those irradiated in normoxia (21 kPa O_2_) [18,22,23,24,25,26,27,28,29,30]. However, critical factors must be considered before assuming that PI application under modified atmosphere will always increase insect emergence, survival or fertility, and, therefore, decrease the efficacy of approved radiation treatments.

The radioprotective effect of hypoxia or anoxia can vary significantly depending on both the absorbed radiation dose and the insect pest species treated at the least radiation sensitive developmental stage commonly found in traded fruit and vegetables. For instance, wandering larvae of *Trichoplusia ni* (Hübner) irradiated in normoxia at 200 Gy failed to complete their development but approximately 15 to 20% of the wandering larvae irradiated at the same dose in near anoxia (0.1 kPa O_2_) emerged as adults [28]. Moreover, irradiating wandering larvae of *T. ni* under severe hypoxia (<2.5 kPa O_2_) or near anoxia (<0.1 kPa O_2_) induced radioprotection and, consequently, increased adult emergence with radiation doses up to 150 Gy, whereas moderate hypoxia (≥5 kPa O_2_) had no detectable radioprotective effect [30]. However, the effect of either severe hypoxia (<2.5 kPa O_2_) or near anoxia (0.1 kPa O_2_) on radiation sensitivity of wandering *T. ni* larvae irradiated at approved or proposed phytosanitary radiation doses for lepidopterans (i.e., 250–400 Gy) remains to be determined. *Rhagoletis pomonella* third instars infesting apples irradiated under near anoxia were not significantly less radiation sensitive than those infesting apples irradiated in normoxia at a maximum absorbed dose of 57 Gy [22]. Similarly, *Bactrocera dorsalis* and *Zeugodacus cucurbitae* (Coquillett) third instars irradiated with the generic dose of 150 Gy for all tephritids and *Ceratitis capitata* third instars irradiated with the low dose of 100 Gy in modified atmosphere packages (either 1–4% O_2_ or 7.2% O_2_) did not emerge as adults [25,29].

In this study, we evaluated the effect of pre-conditioning and irradiation of tephritid third instars under low oxygen levels on adult emergence and whether this potentially decreased the efficacy of phytosanitary radiation treatments. Using the same experimental approach, we simultaneously assessed the effect of hypoxia (~5% O_2_) and severe hypoxia (<0.5% O_2_) compared to normoxia on radiation sensitivity of third instars of *Anastrepha fraterculus* (sensu lato), *A. ludens*, *B. dorsalis*, and *C. capitata* with several radiation doses, including those approved as phytosanitary radiation treatments. Only naturally infested fruits containing tephritid third instars were treated, and sub-samples of dead puparia were dissected to determine the stage of development achieved until the insect death. Our study provides compelling evidence that low levels of oxygen combined with radiation treatments do not reduce the efficacy of phytosanitary irradiation doses approved for tephritid fruit flies. We further discuss the implications of our findings for the application of adopted phytosanitary radiation treatment schedules under modified atmospheres.

## 2. Materials and Methods 

### 2.1. Tephritids

The fruit flies *A. fraterculus* (Brazilian-1 morphotype), *A. ludens*, *B. dorsalis*, and *C. capitata* were used in our study. The *A. fraterculus* colony (~F-35) was collected from infested guavas in Argentina, the *A. ludens* colony (~F-6) from wild-infested oranges in Mexico, the *B. dorsalis* colony (~F-70) from mangoes in Kenya, and the *C. capitata* colony (~F-25) from wild-infested oranges in Argentina. Experiments were carried out using the same colony without the addition of wild flies during the period of our radiation treatments. Colonies were maintained at the Insect Pest Control Laboratory (IPCL) of the Joint FAO/ IAEA Division of Nuclear Techniques in Food and Agriculture in Seibersdorf, Austria. 

Rearing protocols consisted of routinely collecting and transferring eggs to an artificial diet, followed by pupariation and adult maintenance. Briefly, eggs of *A. fraterculus* and *A. ludens* were separately oviposited in silicon sealed devices (13.9 cm diameter) containing water positioned on the top of the cage containing sexually mature adults. *Bactrocera dorsalis* females oviposited eggs in perforated polyethylene bottles (250 ml) containing hundreds of minuscule holes (~0.5 mm diameter). A small quantity of guava juice (≤0.5 ml) was placed inside the bottles and served as an oviposition stimulant for *B. dorsalis*. *Ceratitis capitata* females oviposited eggs through a panel made of voile. Eggs from *A. fraterculus* and *A. ludens* were collected and transferred to an artificial diet based on carrot powder and torula yeast [31,32], whereas the eggs from *B. dorsalis* and *C. capitata* were transferred to an artificial diet based on wheat bran and yeast [33]. Larvae from all species were then held in diet trays until pupariation in sawdust (GOLDSPAN^®^ smoke, Brandenburg, Germany). Puparia were then collected, weighted to determine insect density, transferred to screen-mesh cages, followed by adult emergence. Adults were fed water and a dry diet (3 sucrose: 1 hydrolyzed yeast, MP Biomedicals™, Eschwege, Germany) ad libitum. All insects were maintained under laboratory conditions at 25 ± 0.5°C, 70 ± 10% relative humidity, and 14L:10D photoperiod.

### 2.2. Fruit Infestation, Incubation, and Maintenance

Physiologically mature, but unripe, mangoes (*Mangifera indica* L.—cultivars ‘Tommy Atkins’ and ‘Kent’) from Brazil, Guatemala, Israel, and Senegal, and mandarins (*Citrus reticulata* Blanco) from Israel, Spain, and South Africa were obtained from a local market and used in our experiments. Fruit contamination by microorganisms was minimized by applying multiple sanitization measures before and after natural infestation. Before infestation, mangoes and mandarins were separately washed with soap, rinsed, soaked for 15 min in antifungal solution (4% sodium benzoate and 1% sodium hypochlorite), and re-rinsed. Four to five pre-sanitized mangoes were naturally infested for three to six hours by *A. fraterculus* (~11 days old) and for six to twenty-four hours by *A. ludens* (~15 days old) in a screen-mesh cage (45 × 45 × 45 cm) containing up to 1500 sexually mature insects. For *B. dorsalis* and *C. capitata*, 40 pre-sanitized mandarins were naturally infested in a screen-mesh cage (60 × 60 × 60 cm) containing up to 4500 sexually mature insects (~10 days old) for 45 min to two hours. After infestation, a second sanitization round was applied to all infested fruit to prevent the development of microorganisms. Each fruit was then numbered with a black permanent marker, weighed (digital balance, model IS 32001, VRW, Milan, Italy), and the perimeter measured. Subsequently, numbered mangoes infested by either *A. fraterculus* ($\bar{x}$_mass_ = 511 g ± 5.0, $\bar{x}$_perimeter_ = 29 cm ± 0.5) or *A. ludens* ($\bar{x}$_mass_ = 527g ± 3.6, $\bar{x}$_perimeter_ = 29 cm ± 0.1) were individually placed into plastic containers and incubated at 25 °C for 10–12 days or 14–16 days, respectively. Numbered mandarins infested by either *B. dorsalis* ($\bar{x}$_mass_ = 109 g ± 0.8, $\bar{x}$_perimeter_ = 20 cm ± 0.1) or *C. capitata* ($\bar{x}$_mass_ = 107 g ± 1.5, $\bar{x}$_perimeter_ = 20 cm ± 0.1) were placed into a plastic tray covered with paper towels and sheets of voile tightened by a rubber band and incubated at 25 °C for 8–9 days or 9–10 days, respectively. After the incubation period, most infested fruit were ripe. Late third instars were used in all experiments because they are the least radiation sensitive stage for tephritid fruit fly pre-puparial stages [2].

### 2.3. Low Oxygen Treatments 

Prior to irradiation, infested fruits were transferred to plastic chambers, and then exposed to a specific modified atmosphere treatment. Infested mangoes were placed individually in plastic chambers (13.0 cm diameter × 18.5 cm high) containing a perforated plastic support ring (9.5 cm diameter × 5.5 cm high) positioned on the bottom of the plastic chamber to elevate the fruit to the center of the cylinder to improve radiation dose consistency among treatments. The plastic chambers used to treat infested mangoes were built with a lid containing four sides interlocking, one plastic luer-lock valve (Cole-Parmer^®^, Vernon Hills, IL, USA) attached to the left side of the lid, and a hole (0.6 cm diameter) placed in the right side of the lid to allow the inside air to be expelled during gas flushing. The top hole placed in the mangoes’ chamber was sealed with an adhesive septum (1.5 cm diameter) after gas flushing. For infested mandarins, two fruits were placed inside a galvanized steel-mesh cylinder (7 cm diameter × 15 cm high) containing a rubber stopper (6 cm diameter × 5.5 cm high) at the base, which was used to hold the fruits in the center of the cylinder and improve radiation dose distribution. The galvanized steel-mesh cylinder with the two infested elevated mandarins were then placed inside a plastic chamber (12.5 cm diameter × 19.5 cm high). The plastic chamber used to treat the infested mandarins contained a screw lid on the top sealed with vacuum grease (Dow Corning^®^, Midland, MI, USA), two plastic luer-lock valves (Cole-Parmer^®^, Vernon Hills, USA) attached to the bottom and upper parts for gas flushing, and a hole (0.6 cm diameter) covered by an adhesive septum (1.5 cm diameter) placed on the top side to allow for further determination of the gas concentration inside the chamber. Before gas flushing, Gafchromic™ HD-V2 dosimetry films (Ashland^®^, Bridgewater, NJ, USA) were positioned below and above each mango or mandarin that was later gamma irradiated, allowing the absorbed radiation dose to be measured at different positions.

Two hypoxic atmospheres were achieved by flushing the plastic chambers for up to three minutes with certified gas mixtures (Linde Gas GmbH, Stadl-Paura, Austria) composed of either 5% oxygen, 16% carbon dioxide, 1% argon, and 78% nitrogen (hypoxia) or 0% oxygen, 21% carbon dioxide, 1% argon, and 78% nitrogen (severe hypoxia). These gas mixtures simulated the possible mild to extreme atmospheric conditions during phytosanitary irradiation under controlled atmospheres, such as in apple storage and modified atmosphere packaging, in which oxygen can be partially or completely replaced by carbon dioxide due to fruit respiration [34]. Infested mandarins and mangoes were either conditioned under low oxygen atmospheres (hypoxia or severe hypoxia) for six hours or kept under ambient air (normoxia) before irradiation. Oxygen and carbon dioxide concentrations were monitored hourly using a CheckMate 3 gas analyzer (Dansensor^®^, Ringsted, Denmark) with uncertainties of ±0.01 (0–0.999% O_2_) or ±1.0% (1–100% O_2_) for oxygen and ±0.5% for carbon dioxide. 

### 2.4. Radiation Treatments

Mandarins and mangoes infested with third instars pre-conditioned to a given atmospheric regime were irradiated in a Gammacell 220 (MDS Nordion, Ottawa, Canada) located at the IPCL in Seibersdorf, Austria. Throughout the experiments, the gamma radiation dose rates ranged from 75 to 95 Gy·min^‒1^. Gamma irradiation of infested fruits covered a range of doses with sub-lethal and lethal effects for each fruit fly species. Briefly, mangoes infested with either *A. fraterculus* or *A. ludens* larvae were irradiated with nominal doses of 25, 35, 50, and 70 Gy. Mandarins infested with *B. dorsalis* larvae were irradiated with nominal doses of 30, 40, 80, 116, and 150 Gy. Mandarins infested with *C. capitata* larvae were irradiated with nominal doses of 20, 30, 50, 70, and 100 Gy. Non-irradiated fruits conditioned to normoxia, hypoxia, or severe hypoxia (controls) were handled similarly to irradiated fruits. 

Absorbed dose was verified for all irradiated fruits using a Gafchromic^TM^ dosimetry system (IAEA 2004) with 95% confidence limits that varied between ±1.9% and ±5.9% for different batches of film used for this work. Three pieces (1 × 1 cm) of Gafchromic^TM^ HD-V2 dosimetry film were individually packed in paper envelopes (2.5 × 2.5 cm, FWT-80, Far West Technologies, Goleta, CA, USA), and then sealed in a plastic bag (3.5 × 3.5 cm) to keep them from getting wet. Plastic bags containing three dosimeters were then positioned above and below the infested fruit, allowing the measurement of the absorbed dose at two reference points for mangoes (bottom and top) and three reference points for mandarins (bottom, middle, and top). The dosimeters were read using a portable densitometer (DoseReader 4^®^, RadGen, Budapest, Hungary) approximately 24 h after exposure.

### 2.5. Post-Treatment Evaluations 

Treated and control fruits were individually labelled and placed in plastic containers (21 × 21 × 14.5 cm for mangoes and 9.5 × 9.5 × 11.5 cm for mandarins) until dissection. Mangoes and mandarins were dissected within seven days after treatment, and the number of third instars (dead and live) and puparia were recorded. Live third instars and puparia were transferred to plastic containers (9.5 cm diameter × 5.5 cm high) containing moist sawdust (GOLDSPAN^®^ smoke, Brandenburg, Germany) for further development. Treatment efficacy was determined by prevention of adult emergence. At least a month after pupariation, enough time to ensure that the insects enclosed inside the puparia were dead, we sampled puparia from each treatment and opened them to establish the stage of development at the time of death. Dead insects dissected from the puparia were classified as coarctate larvae, cryptocephalic pupae, phanerocephalic pupae, pharate adults, and partially emerged adults [35,36]. Emerged adults were classified as deformed or fully formed.

### 2.6. Statistics

Adult emergence was adjusted using Abbott’s correction for control mortality and analyzed with generalized linear mixed model (GLMM) for all fruit fly species. Developmental stage achieved after treatment until insect death was evaluated using either GLMM assuming normal distribution or a generalized linear model assuming a binomial distribution and logit link function. Radiation dose, low oxygen treatments and their interaction were modeled as fixed effects. Temporal cohort (block) was modeled as random effect. The statistical significance of the fixed effects and their interaction were determined using either Type III Wald chi-square or F tests. Post-hoc pairwise comparisons of estimated marginal means between the levels of radiation doses and low oxygen treatments were performed with Bonferroni adjustment [37]. Model selection was performed using Akaike’s information criterion [38]. Statistical analyses were carried out in R (version 3.6.1) using *lme4* [39], *brglm2* [40], *bbmle* [41], and *emmeans* [42] packages.

## 3. Results

Emergence rates for *A. fraterculus*, *A. ludens*, *B. dorsalis*, and *C. capitata* infesting irradiated and non-irradiated mangoes or mandarins under normoxia, hypoxia, and severe hypoxia are shown in Table 1, Table 2, Table 3 and Table 4. Dosimetry, oxygen and carbon dioxide measurements were obtained for all infested fruits irradiated under different oxygen levels and are summarized in Table S1 and Table S2. 

As radiation dose increased, adult emergence decreased significantly for *A. fraterculus* (dose: χ^2^ = 777, df = 4, *p* < 0.0001), *A. ludens* (dose: χ^2^ = 340, df = 4, *p* < 0.0001), *B. dorsalis* (dose: χ^2^ = 1759, df = 5, *p* < 0.0001), and *C. capitata* (dose: χ^2^ = 892, df = 5, *p* < 0.0001). Short-term hypoxia or severe hypoxia alone did not affect emergence rates in *B. dorsalis* (atmosphere: χ^2^ = 5, df = 2, *p* = 0.107) and *C. capitata* (atmosphere: χ^2^ = 0.3, df = 2, *p* = 0.873). Conversely, insects infesting non-irradiated mangoes treated with hypoxia exhibited higher emergence rates than those infesting non-irradiated fruits exposed to either normoxia or severe hypoxia in *A. fraterculus* (atmosphere: χ^2^ = 12, df = 2, *p* = 0.003, Table 1) and *A. ludens* (atmosphere: χ^2^ = 13, df = 2, *p* = 0.001, Table 2).

**Figure 1:**
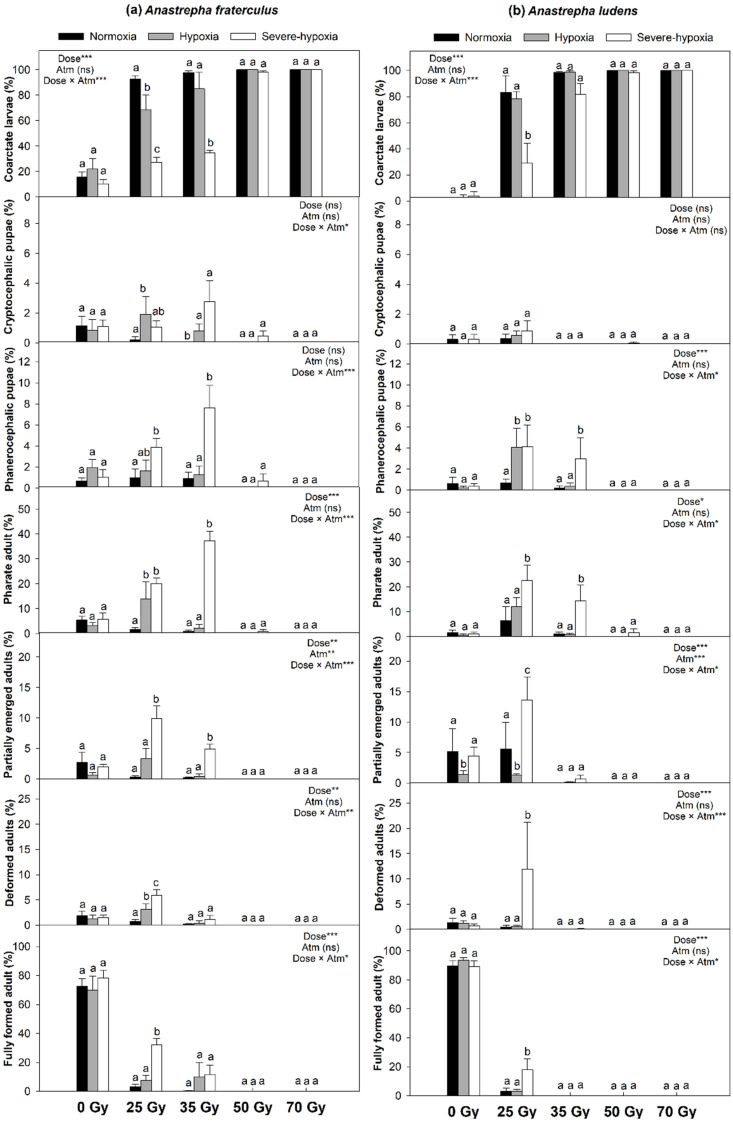
Percentages (mean ± SE) of dead insects dissected from puparia and emerged adults of (**a**) *A. fraterculus* and (**b**) *A. ludens* after irradiation of third instars at different nominal doses and atmospheric conditions and their controls. Generalized linear mixed model (GLMM) for the effect of dose, atmosphere (atm), and their interaction on each developmental stage (‘***’ *p* < 0.0001, ‘**‘ *p* = 0.001, ‘*’ *p* < 0.05, ns = non-significant). Bars followed by different letters within the same radiation dose are significantly different from each other (*p* < 0.05). Details in Table S7 (*A. fraterculus*) and Table S8 (*A. ludens*).

**Figure 2:**
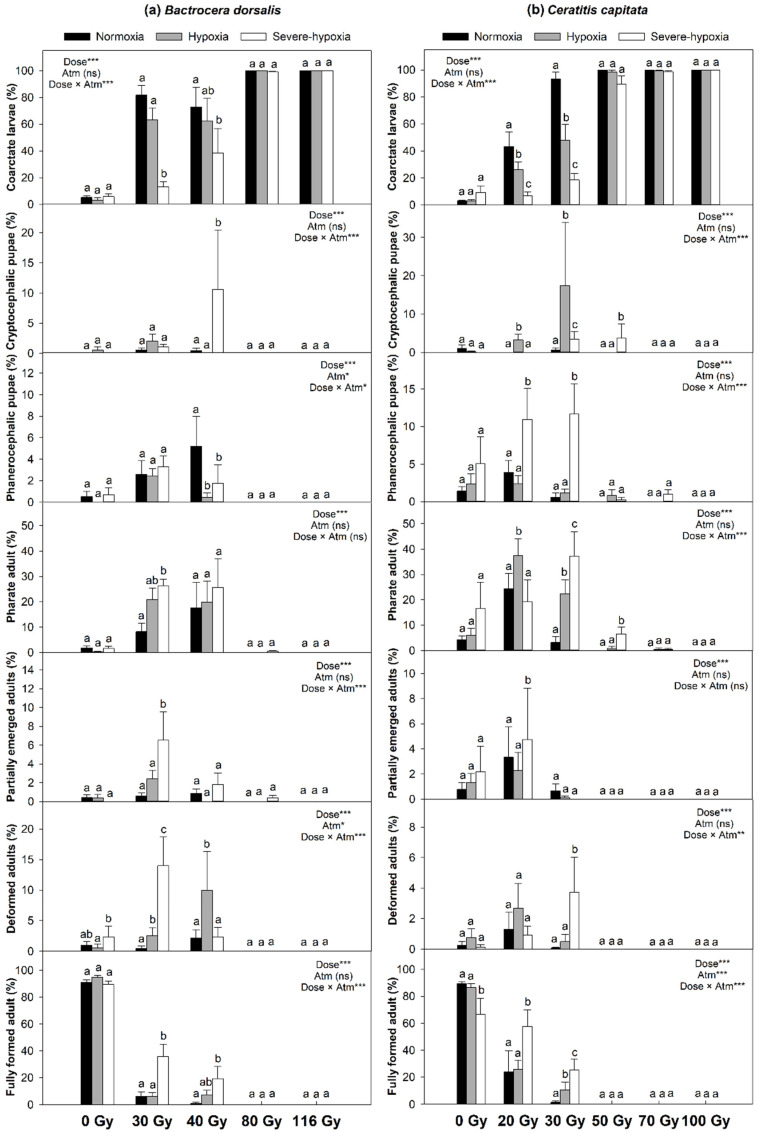
Percentages (mean ± SE) of dead insects dissected from puparia and emerged adults of (**a**) *B. dorsalis* and (**b**) *C. capitata* after irradiation of third instars at different nominal doses and atmospheric conditions and their controls. GLMM models for the effect of dose, atmosphere (atm), and their interaction on each developmental stage (‘***’ *p* < 0.0001, ‘**‘ *p* = 0.001, ‘*’ *p* < 0.05, ns = non-significant). Bars followed by different letters within the same radiation dose are significantly different from each other (*p* < 0.05). Details in Table S9 (*B. dorsalis*) and Table S10 (*C. capitata*).

In general, low oxygen treatments, particularly severe hypoxia, applied before and during irradiation exerted a radioprotective effect and contributed to advancement of larval development only at low doses of irradiation (Figure 1 and Figure 2). The protective effect provided by low oxygen treatments observed in insects irradiated at low doses was not enough to reduce the damage in third instars irradiated at the higher doses recommended for phytosanitary treatments. As a result, no larvae irradiated with phytosanitary doses developed to the pupal or adult stages.

The proportion of insects that died as coarctate larvae after treatment was not affected by low oxygen treatments alone but increased as radiation dose increased, particularly for third instars irradiated under normoxia, in *A. fraterculus* (Figure 1a), *A. ludens* (Figure 1b), *B. dorsalis* (Figure 2a), and *C. capitata* (Figure 2b). The highest numbers of coarctate larvae were observed for insects irradiated in normoxia and the lowest numbers were found for insects irradiated with low doses of gamma rays under severe hypoxia. Regardless of the low oxygen treatment, all insects died as coarctate larvae when irradiated at phytosanitary doses.

Low oxygen treatments and irradiation did not affect the proportion of insects that died as cryptocephalic pupae in *A. ludens* (Figure 1b). In *A. fraterculus*, a higher number of insects died as cryptocephalic pupae when irradiated under hypoxia or severe hypoxia at low doses (25 and 35 Gy) compared to insects irradiated in normoxia (Figure 1a). In *B. dorsalis*, the highest number of cryptocephalic pupae was observed for third instars irradiated at 40 Gy in severe hypoxia (Figure 2a). Third instars of *C. capitata* irradiated with 20, 30 or 50 Gy at low levels of oxygen, particularly hypoxia, died more often as cryptocephalic pupae than insects irradiated in normoxia (Figure 1b).

The proportion of phanerocephalic pupae was higher in third instars irradiated at 25 or 35 Gy under severe hypoxia than in normoxia for *A. fraterculus* (Figure 1a) and *A. ludens* (Figure 1b). In *B. dorsalis*, larvae irradiated at 40 Gy in normoxia died more often as phanerocephalic pupae than third instars irradiated at 40 Gy in either hypoxia or severe hypoxia (Figure 2a). Third instars of *C. capitata* irradiated at 20 or 30 Gy under severe hypoxia survived to the phanerocephalic pupal stage in a higher proportion of cases than insects irradiated in normoxia or hypoxia with the same doses (Figure 2b).

The proportion of insects that reached the pharate adult stage was higher for third instars irradiated at 25 or 35 Gy under low oxygen conditions, particularly in severe hypoxia, than larvae irradiated in normoxia for both *A. fraterculus* (Figure 1a) and *A. ludens* (Figure 1b). In *B. dorsalis*, a higher proportion of third instars irradiated at 30 Gy in severe hypoxia reached the pharate adult stage than insects irradiated in normoxia (Figure 2a). Low oxygen treatments, especially severe hypoxia, before and after irradiation of *C. capitata* third instars at 20 to 50 Gy increased the proportion of insects that reached the pharate adult stage (Figure 2b).

Hypoxia or severe hypoxia alone did not affect the proportion of insects that died as partially emerged adults in *A. fraterculus*, *B. dorsalis*, and *C. capitata*. For *A. ludens*, the proportion of third instars that died as partially emerged adults was lowest for insects exposed to hypoxia, and highest for insects irradiated at 25 Gy under severe hypoxia (Figure 1b). Third instars of *A. fraterculus* irradiated at 25 or 35 Gy died as partially emerged adults more often than insects irradiated in either normoxia or hypoxia (Figure 1a). The proportion of partially emerged adults was higher for third instars irradiated under severe hypoxia at 20 Gy for *C. capitata* (Figure 2b) and at 30 Gy for *B. dorsalis* (Figure 2a) than for insects irradiated at the same doses in either normoxia or hypoxia. 

Low oxygen treatments prior to and during irradiation at sublethal doses had a radioprotective effect in treated third instars, leading to significant increases in the proportion of insects that reached the adult stage. In *A. fraterculus*, irradiation of third instars at 25 Gy under hypoxia or severe hypoxia increased the proportions of deformed and fully formed adults (Figure 1a). In *A. ludens*, the proportions of third instars irradiated at 25 Gy under severe hypoxia that emerged as deformed or fully formed adults (Figure 1b) were higher than for insects irradiated in either normoxia or hypoxia. In *B. dorsalis*, severe hypoxia alone increased the proportion of third instars that developed into deformed adults and, when combined with radiation doses of 30 or 40 Gy, hypoxia and severe hypoxia increased the proportions of insects that developed into deformed or fully formed adults (Figure 2a). In *C. capitata*, severe hypoxia alone reduced the proportion of third instars that developed into fully formed adults, and a higher proportion of third instars irradiated at 20 or 30 Gy under low oxygen treatments developed into deformed or fully formed adult compared to insects irradiated in normoxia (Figure 2b).

## 4. Discussion

All third instars of *A. fraterculus*, *A. ludens*, *B. dorsalis*, and *C. capitata* pre-conditioned and irradiated under normoxia, hypoxia (~5% O_2_) or severe hypoxia (<0.5% O_2_) with approved phytosanitary doses failed to emerge as adults and died as coarctate larvae. Radioprotective effects of low oxygen treatments were observed only in larvae irradiated with low doses of gamma rays that are not used as phytosanitary treatments. These findings corroborate previous studies evaluating the effect of low oxygen treatments on PI efficacy using either the same or complementary experimental approaches for several fruit fly species. Two experimental approaches have been used in studies evaluating the effect of low oxygen atmospheres on PI treatments for plant pest insects: gas flushing to replace the atmospheric oxygen and modified atmosphere packaging (MAP). 

The replacement of atmospheric oxygen by flushing with nitrogen or certified gas mixtures has been applied in PI studies with dipterans, lepidopterans, and coleopterans, including the fruit fly species *R. pomonella* [18,22,23,24,27,28,30]. In the fruit fly study, third instars maintained in a nitrogen rich atmosphere under severe hypoxic conditions before, during, and after irradiation showed decreased radiation sensitivity to a dose of 30 Gy compared with insects irradiated in air. However, no difference was found with absorbed doses around 57 Gy [22]. The evidence that low oxygen levels do not reduce the efficacy of the radiation dose used as PI treatment for *R. pomonella* supported the adoption of a phytosanitary treatment without restrictions regarding modified atmospheres by the IPPC [14]. Based on the same experimental approach used for *R. pomonella*, our study showed a similar trend for *A. fraterculus*, *A. ludens*, *B. dorsalis*, and *C. capitata*. That is, PI treatments against these fruit fly species under hypoxic (~5 O_2_, ~16% CO_2_) and severe hypoxic (<0.5 O_2_, ~21% CO_2_) conditions, as expected, decreased radiation sensitivity only with low doses of gamma rays but failed to provide sufficient radioprotection to enable fruit fly development to the adult stage with radiation doses approved as phytosanitary treatments. 

As an applied experimental approach, MAP has been used to evaluate the effect of reduced oxygen and increased carbon dioxide concentrations on PI efficacy under realistic commercial conditions and durations [43]. Despite the limited precision of the oxygen and carbon dioxide concentrations usually obtained in MAP bags [25], this approach has contributed considerably to our understanding of the effect of modified atmospheres on PI efficacy for *Drosophila suzukii* and some tephritid species [25,26,29]. For tephritid fruit flies, the findings obtained by the research on PI under MAP conditions corroborate those from gas flushing studies, including ours. Third instars of *B. dorsalis*, *C. capitata* and *Z. cucurbitae* pre-conditioned and irradiated in MAP bags (1 to 15% O_2_, 8 to 22% CO_2_) did not emerge as adults after irradiation with approved PI doses [25,29]. A small increase in radioprotection, measured in terms of adult emergence, was reported for *Z. cucurbitae* third instars irradiated with 50 Gy under MAP conditions with oxygen levels varying from 1 to 4%, but it was not statistically significant [25].

The radioprotective effect of low oxygen treatments in fruit fly third instars irradiated with sublethal doses observed in our study may be explained by the activation of highly conserved cellular mechanisms in response to hypoxia [44]. In general, insects exposed to functional hypoxia—a condition characterized by the inadequate oxygen supply to meet the ATP demands of an organism despite compensations [45], can use acute and chronic cellular responses to prevent damage due to oxygen deprivation [46]. Short-term strategies mediated by sensory neurons drive acute responses, whereas long-term strategies mediated by hypoxia-inducible factors (HIF), among others [20,21,47], drive chronic responses to ensure normal development and survival [45,48,49,50,51,52,53]. In *B. dorsalis*, for example, exposure of third instars to a severe hypoxic treatment (~0.2% O_2_) for up to 15 h induced the expression of HIF-1α and HIF-1-responsive genes, which, ultimately, rescued survival after reoxygenation [54]. Interestingly, the HIF-1α and HIF-1-responsive genes expressed in *B. dorsalis* were responsive to severe hypoxic treatments similar to those used in our study. Thus, we can speculate that the activation of HIF-1α and HIF-1-responsive genes might be one of the mechanisms underlying the partial radioprotection reported in the present study for fruit fly third instars irradiated with sublethal doses under hypoxia and severe hypoxia. These HIF-1-responsive genes expressed by third instars of *B. dorsalis*, particularly those encoding heat shock proteins and superoxide dismutases 1 and 2, have a critical role in the cellular response against several stress factors [55,56].

The transient upregulation of antioxidant enzymes during hypoxia may also offer protection against future stressors, as observed in many cases of physiological conditioning hormesis. For instance, beetles, fruit flies, and moths exposed to low oxygen treatments showed increased antioxidant activity that later protected them from oxidative stress induced by ionizing radiation [18,19,57]. However, the induction of such radioprotective effects in phytosanitary irradiation may depend on oxygen and carbon dioxide levels, duration of exposure, and radiation dose applied during the treatment [1,2,30,43]. It has been suggested that oxygen levels above 5 kPa apparently do not induce greater radioprotection in insects, an aspect that must be tested across different species taking into consideration carbon dioxide levels as well [30]. For tephritid fruit flies, the radioprotection offered by hypoxic and severe hypoxic exposures before and during irradiation seems to be limited to sublethal doses of radiation that are not used as PI treatments. We have shown the significant lethality of radiation doses approved as phytosanitary treatments by confirmed death of treated insects as coarctate larvae. In such cases, it is very likely that the oxidative damage generated by PI doses was too high to be repaired. Perhaps PI under low oxygen atmospheres may not be a significant problem for tephritids irradiated at approved phytosanitary irradiation doses because they are already in hypoxic conditions inside intact fruit [58,59], thus, already experiencing reduced detrimental effects of radiation. We have accounted for this factor by using only naturally infested fruits in our experiments. 

There is enough evidence supporting the fact that modified atmospheres do not reduce the efficacy of phytosanitary irradiation for tephritids. Furthermore, modified atmosphere itself can be used as a phytosanitary treatment [60], and the hormetic effect of low oxygen conditioning can be reverted with longer durations of hypoxia that are harmful to organisms [21]. Based on these findings and considerations, regulatory plant protection agencies and international organizations may consider reviewing their restrictions on PI under modified atmospheres targeting tephritid fruit flies. Future studies should focus on the effect of modified atmospheres on phytosanitary irradiation efficacy using other insect taxa and considering approved PI doses in their experimental designs. The role played by carbon dioxide levels combined with low oxygen treatments, seems to be a relevant factor that also deserves some attention, as pointed out by other authors [30]. 

## 5. Conclusions

Our study provides evidence that hypoxic and severe-hypoxic conditioning before and during irradiation can only increase adult emergence and contribute to larval development advancement of tephritid fruit flies at low doses of gamma radiation that are not used as phytosanitary treatments. At the doses used for phytosanitation purposes, low oxygen treatments did not increase the emergence rate of any fruit fly species evaluated, and all treated insects died as coarctate larvae. Thus, international organizations and regulatory agencies may consider re-evaluating restrictions related to phytosanitary irradiation applications under modified atmospheres targeting tephritid fruit flies.

## Figures and Tables

**Table 1:** Numbers of replicates, larvae per fruit (mean ± SE), treated larvae, dead insects, and adult survival (mean ± SE) of *Anastrepha fraterculus* third instars irradiated at different nominal doses and atmospheric conditions.

Dose (Gy)	Atmospheric Conditions ^1^	n	No. Larvae Per Fruit	Total No. Larvae Treated	Total No. Insects Dead	Adult Emergence (%) ^2^
0	Normoxia	36	180 ± 33	8976	2051	75.8 ± 2.4 AB
	Hypoxia	33	222 ± 36	7318	1078	80.5 ± 2.3 A
	Severe hypoxia	40	162 ± 26	6475	1243	70.6 ± 3.7 B
25	Normoxia	30	210 ± 29	6287	6141	2.2 ± 0.5 A
	Hypoxia	32	268 ± 37	8578	8187	6.0 ± 1.3 A
	Severe hypoxia	27	265 ± 50	7145	4771	38.7 ± 4.3 B
35	Normoxia	12	207 ± 47	2483	2476	0.2 ± 0.3 A
	Hypoxia	10	327 ± 94	3275	3274	0.3 ± 0.0 A
	Severe hypoxia	12	225 ± 78	2701	2625	5.5 ± 3.0 A
50	Normoxia	17	131 ± 24	2224	2224	0.0 ± 0.0 A
	Hypoxia	11	224 ± 66	2462	2462	0.0 ± 0.0 A
	Severe hypoxia	17	136 ± 27	2318	2318	0.0 ± 0.0 A
70	Normoxia	28	196 ± 32	5501	5501	0.0 ± 0.0 A
	Hypoxia	31	191 ± 30	5911	5911	0.0 ± 0.0 A
	Severe hypoxia	32	216 ± 39	6896	6896	0.0 ± 0.0 A

^1^ Normoxia (~21.0% O_2_, 0.0% CO_2_), hypoxia (5.5 ± 0.1% O_2_, 15.7 ± 0.2% CO_2_) and severe hypoxia (0.3 ± 0.02% O_2_, 22.2 ± 0.2% CO_2_). ^2^ Different letters within the same radiation dose indicate significant differences (estimated marginal means contrasts, *p* < 0.05). Raw data are available in Table S3.

**Table 2:** Numbers of replicates, larvae per fruit (mean ± SE), treated larvae, dead insects, and adult emergence (mean ± SE) of *Anastrepha ludens* third instars irradiated at different nominal doses and atmospheric conditions.

Dose (Gy)	Atmospheric Conditions ^1^	n	No. Larvae Per Fruit	Total No. Larvae Treated	Total No. Insects Dead	Adult Emergence (%) ^2^
0	Normoxia	27	109 ± 15	5985	1723	68.3 ± 3.1 A
	Hypoxia	18	209 ± 44	3757	648	81.3 ± 2.6 B
	Severe hypoxia	24	161 ± 30	3863	1247	64.7 ± 4.5 A
25	Normoxia	38	232 ± 30	8797	8602	2.1 ± 0.5 A
	Hypoxia	25	222 ± 38	5539	5261	9.3 ± 4.3 A
	Severe hypoxia	26	102 ± 23	2639	1891	35.6 ± 5.4 B
35	Normoxia	25	164 ± 27	4088	4082	0.1 ± 0.1 A
	Hypoxia	12	239 ± 65	2864	2863	0.1 ± 0.1 A
	Severe hypoxia	16	145 ± 35	2315	2293	1.5 ± 0.9 A
50	Normoxia	23	119 ± 30	2731	2731	0.0 ± 0.0 A
	Hypoxia	12	250 ± 62	2996	2996	0.0 ± 0.0 A
	Severe hypoxia	19	170 ± 36	3237	3233	0.1 ± 0.1 A
70	Normoxia	20	100 ± 26	1990	1990	0.0 ± 0.0 A
	Hypoxia	14	176 ± 49	2468	2468	0.0 ± 0.0 A
	Severe hypoxia	19	128 ± 38	2435	2435	0.0 ± 0.0 A

^1^ Normoxia (~21.0% O_2_, 0.0% CO_2_), hypoxia (5.2 ± 0.1% O_2_, 15.8 ± 0.2% CO_2_) and severe hypoxia (0.3 ± 0.03% O_2_, 21.4 ± 0.1% CO_2_). ^2^ Different letters within the same radiation dose indicate significant differences (estimated marginal means contrasts, *p* < 0.05). Raw data are available in Table S4.

**Table 3:** Numbers of replicates, larvae per fruit (mean ± SE), treated larvae, dead insects, and adult emergence (mean ± SE) of *Bactrocera dorsalis* third instars irradiated at different nominal doses and atmospheric conditions.

Dose (Gy)	Atmospheric Conditions ^1^	n	No. Larvae Per Fruit	Total No. Larvae Treated	Total No. Insects Dead	Adult Emergence (%) ^2^
0	Normoxia	36	123 ± 10	18,397	3057	84.7 ± 1.5 A
	Hypoxia	24	86 ± 11	4050	844	82.4 ± 2.7 A
	Severe hypoxia	47	108 ± 10	11,168	2269	81.0 ± 2.2 A
30	Normoxia	8	78 ± 17	1172	1105	5.5 ± 1.8 A
	Hypoxia	16	141 ± 25	4523	3872	14.4 ± 3.6 A
	Severe hypoxia	7	101 ± 21	1508	816	45.9 ± 5.6 B
40	Normoxia	41	119 ± 17	4899	4787	3.8 ± 1.1 A
	Hypoxia	34	133 ± 21	4523	4264	12.0 ± 3.2 A
	Severe hypoxia	43	89 ± 14	3820	3143	21.8 ± 3.8 B
80	Normoxia	16	143 ± 33	2289	2288	0.01 ± 0.01 A
	Hypoxia	31	116 ± 22	3699	3699	0.0 ± 0.0 A
	Severe hypoxia	14	76 ± 23	1069	1069	0.0 ± 0.0 A
116	Normoxia	48	70 ± 8	6405	6405	0.0 ± 0.0 A
	Hypoxia	32	80 ± 12	4967	4967	0.0 ± 0.0 A
	Severe hypoxia	36	63 ± 9	4511	4511	0.0 ± 0.0 A
150	Normoxia	33	187 ± 31	6175	6175	0.0 ± 0.0 A
	Hypoxia	28	66 ± 10	1852	1852	0.0 ± 0.0 A
	Severe hypoxia	28	141 ± 20	3938	3938	0.0 ± 0.0 A

^1^ Normoxia (~21.0% O_2_, 0.0% CO_2_), hypoxia (5.3 ± 0.04% O_2_, 15.0 ± 0.1% CO_2_) and severe hypoxia (0.3 ± 0.02% O_2_, 21.6 ± 0.1% CO_2_). ^2^ Different letters within the same radiation dose indicate significant differences (estimated marginal means contrasts, *p* < 0.05). Raw data are available in Table S5.

**Table 4:** Numbers of replicates, larvae per fruit (mean ± SE), treated larvae, dead insects, and adult emergence (mean ± SE) of *Ceratitis capitata* third instars irradiated at different nominal doses and atmospheric conditions.

Dose (Gy)	Atmospheric Conditions ^1^	n	No. Larvae Per Fruit	Total No. Larvae Treated	Total No. Insects Dead	Adult Emergence (%) ^2^
0	Normoxia	21	69 ± 7	5901	1293	78.3 ± 2.5 A
	Hypoxia	9	63 ± 19	1004	267	74.8 ± 3.1 A
	Severe hypoxia	29	46 ± 6	2462	424	77.6 ± 3.6 A
20	Normoxia	4	76 ± 21	529	514	10.5 ± 5.6 A
	Hypoxia	10	49 ± 14	977	796	29.1 ± 6.2 B
	Severe hypoxia	10	66 ± 18	1320	591	67.3 ± 6.0 C
30	Normoxia	12	78 ± 15	2590	2470	3.6 ± 1.6 A
	Hypoxia	9	32 ± 9	543	489	12.2 ± 6.0 AB
	Severe hypoxia	12	44 ± 9	1053	846	19.8 ± 5.2 B
50	Normoxia	9	73 ± 36	654	653	0.6 ± 0.6 A
	Hypoxia	20	62 ± 16	1248	1247	0.2 ± 0.2 A
	Severe hypoxia	20	44 ± 11	880	880	0.0 ± 0.0 A
70	Normoxia	6	70 ± 18	1031	1031	0.0 ± 0.0 A
	Hypoxia	11	124 ± 27	2727	2727	0.0 ± 0.0 A
	Severe hypoxia	10	86 ± 21	1334	1334	0.0 ± 0.0 A
100	Normoxia	39	39 ± 5	2969	2969	0.0 ± 0.0 A
	Hypoxia	19	59 ± 9	2138	2138	0.0 ± 0.0 A
	Severe hypoxia	33	27 ± 4	1550	1550	0.0 ± 0.0 A

^1^ Normoxia (~21.0% O_2_, 0.0% CO_2_), hypoxia (5.2 ± 0.05% O_2_, 15.6 ± 0.1% CO_2_) and severe hypoxia (0.4 ± 0.03% O_2_, 21.5 ± 0.1% CO_2_). ^2^ Different letters within the same radiation dose indicate significant differences (estimated marginal means contrasts, *P* < 0.05). Raw data are available in Table S6.

## Supplementary Materials

The following are available online at https://www.mdpi.com/2075-4450/11/6/371/s1, Table S1. Dosimetry, oxygen (O_2_) and carbon dioxide (CO_2_) levels for infested mangoes irradiated at different doses in normoxia, hypoxia, and severe hypoxia atmospheres; Table S2. Dosimetry, oxygen (O_2_) and carbon dioxide (CO_2_) levels for infested mandarins irradiated at different doses in normoxia, hypoxia, and severe hypoxia atmospheres; Table S3. Experimental data for *Anastrepha fraterculus*. Table S4. Experimental data for *Anastrepha ludens*. Table S5. Experimental data for *Bactrocera dorsalis*. Table S6. Experimental data for *Ceratitis capitata*; Table S7. Percentages (mean ± SE (total number of individuals)) of dead insects dissected from puparia (CAL, CCP, PCP, PHA, PEA) and emerged adults (DFA, FFA) of *Anastrepha fraterculus* after irradiation of third instars at different nominal doses and atmospheric conditions; Table S8. Percentages (mean ± SE (total number of individuals)) of dead insects dissected from puparia (CAL, CCP, PCP, PHA, PEA) and emerged adults (DFA, FFA) of *Anastrepha ludens* after irradiation of third instars at different nominal doses and atmospheric conditions; Table S9. Percentages (mean ± SE (total number of individuals)) of dead insects dissected from puparia (CAL, CCP, PCP, PHA, PEA) and emerged adults (DFA, FFA) of *Bactrocera dorsalis* after irradiation of third instars at different nominal doses and atmospheric conditions; Table S10. Percentages (mean ± SE (total number of individuals)) of dead insects dissected from puparia (CAL, CCP, PCP, PHA, PEA) and emerged adults (DFA, FFA) of *Ceratitis capitata* after irradiation of third instars at different nominal doses and atmospheric conditions.
