# Supplementary material for: Modified Atmosphere Does Not Reduce the Efficacy of Phytosanitary Irradiation Doses Recommended for Tephritid Fruit Flies
Source: Insects. 2020 Jun 15;11(6):371. doi: 10.3390/insects11060371 (PMC7348963; doi:10.3390/insects11060371)
Supplement: Supplementary file 1 [file insects-11-00371-s001.pdf]

insects MDPI

Supplementary materials

Modified atmosphere does not reduce the efficacy of phytosanitary irradiation doses recommended for tephritid fruit flies

Vanessa S. Dias [1,†,*], Guy J. Hallman [2,†,*], Olga Y. Martínez-Barrera [1], Nick V. Hurtado [1], Amanda A. S. Cardoso [1], Andrew G. Parker [1], Luis A. Caravantes [1], Camilo Rivera [1], Alexandre S. Araújo [1], Florence Maxwell [1], Carlos E. Cáceres-Barrios [1], Marc J. B. Vreysen [1], and Scott W. Myers [3]

1 Insect Pest Control Laboratory, Joint FAO/IAEA Division of Nuclear Techniques in Food and Agriculture, IAEA, Wagramer Strasse 5, 1400 Vienna, Austria
2 Phytosanitation, 3917 Estancia Drive, Oceanside, CA 92058, United States of America
3 USDA, APHIS, PPQ, Science and Technology, Otis Laboratory 1398 W. Truck Rd., Buzzards Bay, MA 02542, United States of America
† These authors contributed equally to the work
* Correspondence: V.Dias@iaea.org, vanessasidias@hotmail.com (VSD); n5551212@yahoo.com (GJH)

Table S1. Dosimetry, oxygen (O_2) and carbon dioxide (CO_2) levels for infested mangoes irradiated at different doses in normoxia, hypoxia, and severe hypoxia atmospheres

Tephritid species	Atmospheric conditions	O_2(%) [mean ± SE]	CO_2 (%) [mean ± SE]	Nominal dose	Absorbed dose (mean ± SE) [min, max]	
					Bottom (Gy)	Up (Gy)
Anastrepha fraterculus	Normoxia	21	0	0 Gy	-	
	Hypoxia	5.64 ± 0.11	15.55 ± 0.40			
	Severe hypoxia	0.32 ± 0.04	22.47 ± 0.35			
	Normoxia	21	0	25 Gy	24.47 ± 0.56 [21, 34]	27.42 ± 0.59 [21, 29]
	Hypoxia	5.52 ± 0.11	15.91 ± 0.48		24.47 ± 0.56 [21, 30]	26.19 ± 0.33 [20, 30]
	Severe hypoxia	0.34 ± 0.06	22.41 ± 0.36		25.52 ± 0.82 [22, 39]	27.42 ± 0.59 [21, 34]
	Normoxia	21	0	35 Gy	37.74 ± 1.15 [32, 44]	34.20 ± 0.68 [30, 38]
	Hypoxia	4.98 ± 0.17	17.38 ± 0.72		39.68 ± 0.90 [36, 44]	37.85 ± 1.28 [32, 45]
	Severe hypoxia	0.40 ± 0.03	21.46 ± 0.31		39.89 ± 1.37 [31, 49]	38.84 ± 1.64 [31, 48]
	Normoxia	21	0	50 Gy	55.51 ± 1.23 [45, 69]	52.57 ± 1.10 [43, 59]
	Hypoxia	5.01 ± 0.12	16.38 ± 0.65		57.10 ± 0.63 [53, 60]	54.22 ± 1.09 [48, 60]
	Severe hypoxia	0.41 ± 0.04	22.33 ± 0.44		57.65 ± 0.89 [52, 66]	52.46 ± 1.37 [40, 64]
	Normoxia	21	0	70 Gy	66.01 ± 1.12 [59, 88]	73.51 ± 1.01 [59, 84]
	Hypoxia	5.70 ± 0.12	15.00 ± 0.36		67.18 ± 1.28 [57, 85]	75.30 ± 0.89 [62, 85]
	Severe hypoxia	0.33 ± 0.05	22.19 ± 0.39		69.66 ± 1.33 [59, 88]	74.88 ± 0.88 [57, 85]
Anastrepha ludens	Normoxia	21.0	0.0	0 Gy	-	
	Hypoxia	5.32 ± 0.17	15.89 ± 0.25			
	Severe hypoxia	0.29 ± 0.04	21.40 ± 0.29			
	Normoxia	21	0	25 Gy	25.26 ± 0.33 [21, 30]	26.41 ± 0.33 [24, 33]
	Hypoxia	5.23 ± 0.12	15.68 ± 0.33		26.78 ± 0.57 [21, 31]	26.82 ± 0.44 [24, 32]
	Severe hypoxia	0.33 ± 0.07	21.30 ± 0.17		28.86 ± 0.62 [21, 35]	26.76 ± 0.44 [22, 34]
	Normoxia	21	0	35 Gy	34.43 ± 0.53 [30, 40]	32.41 ± 0.71 [26, 42]
	Hypoxia	5.05 ± 0.10	16.77 ± 0.71		40.76 ± 1.11 [37, 48]	37.74 ± 1.15 [32, 44]
	Severe hypoxia	0.28 ± 0.05	21.49 ± 0.37		36.99 ± 1.27 [47, 67]	34.59 ± 1.21 [27, 42]
	Normoxia	21	0	50 Gy	55.34 ± 1.01 [47, 67]	51.29 ± 1.24 [37, 67]
	Hypoxia	4.90 ± 0.08	15.96 ± 0.55		56.97 ± 1.19 [49, 63]	52.40 ± 0.96 [47, 58]
	Severe hypoxia	0.27 ± 0.04	21.33 ± 0.33		56.01 ± 1.37 [47, 67]	51.93 ± 1.18 [42, 67]
	Normoxia	21	0	70 Gy	68.80 ± 1.72 [56, 87]	74.56 ± 1.25 [63, 85]
	Hypoxia	5.40 ± 0.16	15.00 ± 0.43		72.87 ± 2.19 [63, 88]	75.35 ± 0.94 [70, 84]
	Severe hypoxia	0.40 ± 0.08	21.56 ± 0.38		70.95 ± 1.70 [57, 84]	73.95 ± 1.27 [60, 81]

Table S2. Dosimetry, oxygen (O2) and carbon dioxide (CO2) levels for infested mandarins irradiated at different doses in normoxia, hypoxia, and severe hypoxia atmospheres

Tephritid species	Atmospheric conditions	O_2(%) [mean ± SE]	CO_2 (%) [mean ± SE]	Nominal dose	Absorbed dose (mean ± SE) [min, max]		
					Bottom (Gy)	Middle (Gy)	Up (Gy)
Bactrocera dorsalis	Normoxia	21	0	0 Gy	-		
	Hypoxia	5.23 ± 0.09	14.76 ± 0.21				
	Severe hypoxia	0.28 ± 0.03	21.74 ± 0.13				
	Normoxia	21	0	30 Gy	34.48 ± 1.21 [28, 41]	37.57 ± 1.10 [31, 43]	38.03 ± 1.55 [33, 45]
	Hypoxia	5.34 ± 0.15	15.03 ± 0.27		35.06 ± 0.56 [31, 42]	37.85 ± 0.68 [31, 44]	35.86 ± 0.72 [26, 41]
	Severe hypoxia	0.56 ± 0.08	21.41 ± 0.33		35.95 ± 0.87 [32, 40]	37.99 ± 0.90 [32, 42]	38.44 ± 1.07 [33, 45]
	Normoxia	21	0	40 Gy	46.47 ± 0.89 [39, 59]	51.73 ± 1.46 [42, 62]	46.90 ± 0.78 [39, 56]
	Hypoxia	5.36 ± 0.09	14.54 ± 0.15		45.72 ± 0.63 [39, 52]	46.70 ± 0.67 [40, 52]	45.42 ± 0.74 [35, 54]
	Severe hypoxia	0.38 ± 0.04	21.50 ± 0.14		48.40 ± 0.99 [41, 59]	55.43 ± 1.28 [41, 67]	46.68 ± 0.85 [38, 56]
	Normoxia	21	0	80 Gy	72.58 ± 2.23 [65, 96]	82.65 ± 1.46 [71, 90]	77.33 ± 1.65 [69, 91]
	Hypoxia	5.10 ± 0.09	14.54 ± 0.15		79.73 ± 1.70 [62, 94]	96.12 ± 0.83 [77, 94]	79.78 ± 1.69 [66, 96]
	Severe hypoxia	0.68 ± 0.08	20.90 ± 0.17		78.97 ± 2.75 [63, 90]	82.09 ± 1.38 [74, 89]	82.94 ± 2.60 [70, 98]
	Normoxia	21	0	116 Gy	114.54 ± 0.75 [101, 131]	121.63 ± 0.72 [101, 136]	110.06 ± 0.99 [94, 131]
	Hypoxia	5.27 ± 0.08	14.52 ± 0.15		117.50 ± 1.26 [99, 135]	123.12 ± 1.45 [95, 149]	121.33 ± 2.04 [85, 158]
	Severe hypoxia	0.37 ± 0.03	21.49 ± 0.15		118.41 ± 0.84 [104, 132]	121.63 ± 0.72 [114, 152]	117.82 ± 1.79 [98, 152]
	Normoxia	21	0	150 Gy	148.99 ± 0.85 [142, 164]	155.81 ± 0.49 [142, 173]	136.68 ± 1.63 [120, 184]
	Hypoxia	5.39 ± 0.15	14.84 ± 0.22		152.01 ± 3.02 [127, 186]	164.89 ± 3.11 [143, 194]	159.50 ± 3.62 [130, 197]
	Severe hypoxia	0.21 ± 0.04	22.45 ± 0.50		151.58 ± 1.27 [142, 161]	156.10 ± 1.48 [142, 173]	136.80 ± 3.18 [123, 157]

Table S2. Continued.

-Ceratitis capitata	Normoxia	21	0	0 Gy	-		
	Hypoxia	5.11 ± 0.11	15.54 ± 0.29				
	Severe hypoxia	0.30 ± 0.06	21.63 ± 0.14				
	Normoxia	21	0	20 Gy	20.63 ± 0.76 [18, 23]	21.97 ± 0.85 [19, 25]	20.14 ± 0.68 [19, 23]
	Hypoxia	5.03 ± 0.11	15.98 ± 0.21		21.63 ± 0.33 [19, 24]	23.13 ± 0.57 [19, 28]	20.06 ± 0.62 [16, 25]
	Severe hypoxia	0.56 ± 0.03	21.26 ± 0.12		21.52 ± 0.42 [19, 25]	21.59 ± 0.31 [20, 25]	19.14 ± 0.48 [19, 23]
	Normoxia	21	0	30 Gy	32.41 ± 0.47 [26, 38]	36.38 ± 1.03 [27, 44]	31.61 ± 0.52 [25, 35]
	Hypoxia	5.18 ± 0.12	15.39 ± 0.14		33.14 ± 0.46 [30, 36]	34.84 ± 0.58 [31, 39]	33.51 ± 0.95 [25, 38]
	Severe hypoxia	0.43 ± 0.03	21.64 ± 0.31		31.93 ± 0.59 [27, 35]	34.69 ± 0.69 [27, 40]	30.82 ± 0.65 [26, 38]
	Normoxia	21	0	50 Gy	49.57 ± 0.59 [48, 52]	53.77 ± 1.06 [50, 58]	49.57 ± 0.87 [46, 53]
	Hypoxia	5.16 ± 0.15	15.92 ± 0.21		53.48 ± 0.99 [47, 60]	56.27 ± 0.73 [52, 62]	53.20 ± 1.05 [49, 63]
	Severe hypoxia	0.59 ± 0.04	20.88 ± 0.06		52.06 ± 0.50 [48, 56]	53.76 ± 0.76 [47, 58]	55.46 ± 1.07 [45, 65]
	Normoxia	21	0	70 Gy	70.62 ± 1.13 [64, 76]	75.47 ± 0.90 [71, 79]	71.60 ± 0.98 [67, 76]
	Hypoxia	5.48 ± 0.10	15.44 ± 0.17		70.84 ± 0.79 [61, 75]	75.01 ± 1.19 [65, 88]	72.95 ± 0.89 [69, 83]
	Severe hypoxia	0.59 ± 0.03	21.06 ± 0.09		70.54 ± 0.86 [63, 75]	77.09 ± 1.12 [71, 85]	75.45 ± 0.88 [72, 84]
	Normoxia	21	0	100 Gy	101.74 ± 0.73 [85, 118]	108.69 ± 0.95 [95, 128]	97.08 ± 0.89 [81, 113]
	Hypoxia	5.08 ± 0.08	15.46 ± 0.16		104.50 ± 1.39 [91, 123]	108.91 ± 1.25 [94, 123]	111.74 ± 1.08 [100, 127]
	Severe hypoxia	0.26 ± 0.03	21.68 ± 0.16		103.57 ± 0.78 [93, 120]	108.16 ± 0.86 [91, 129]	96.67 ± 1.24 [76, 115]

Table S3. Experimental data for *Anastrepha fraterculus*

Block	Nominal dose	Absorbed dose: bottom	Absorbed dose: top	Atmospheric conditions	O_2(%)	CO_2 (%)	Adult emergence (corrected)	No. insects treated
1	0	0	0	Normoxia	21	0	56.05095541	471
1	25	21.5	23.9	Normoxia	21	0	1.054745118	439
1	70	60.8	78.4	Normoxia	21	0	0	608
1	25	22.2	23.6	Normoxia	21	0	10.84237303	242
1	70	61.7	63.4	Normoxia	21	0	0	272
1	0	0	0	Normoxia	21	0	73.52941176	34
2	0	0	0	Normoxia	21	0	75	8
2	25	23.7	24.1	Normoxia	21	0	0	35
2	70	62.2	74.4	Normoxia	21	0	0	54
3	0	0	0	Normoxia	21	0	88.23529412	17
3	25	20.6	26.7	Normoxia	21	0	0	34
3	70	62.2	76.1	Normoxia	21	0	0	20
4	0	0	0	Normoxia	21	0	66.66666667	87
4	25	22.2	27.3	Normoxia	21	0	0	41
4	70	62.9	75	Normoxia	21	0	0	42
5	0	0	0	Normoxia	21	0	40	10
5	25	21.4	25.7	Normoxia	21	0	2.673796791	187
5	70	59.4	78.8	Normoxia	21	0	0	275
6	25	22.9	25.9	Normoxia	21	0	0	18
6	70	57.3	75.2	Normoxia	21	0	0	32
6	0	0	0	Normoxia	21	0	65.2173913	322
7	0	0	0	Normoxia	21	0	91.51291513	271
7	25	23.3	29.2	Normoxia	21	0	0	37
8	0	0	0	Normoxia	21	0	85.35353535	198
8	25	23.3	26.2	Normoxia	21	0	2.19176428	588
8	70	71.8	83.7	Normoxia	21	0	0	396
9	0	0	0	Normoxia	21	0	86.60436137	321
9	25	22	28.5	Normoxia	21	0	0	166
9	70	62	80.7	Normoxia	21	0	0	666
10	0	0	0	Normoxia	21	0	93.48534202	307
10	25	20.5	22.3	Normoxia	21	0	0	152
10	70	59.3	81.5	Normoxia	21	0	0	116
11	0	0	0	Normoxia	21	0	95.65217391	575
11	25	24	27.2	Normoxia	21	0	1.412776413	74
11	70	58	75.7	Normoxia	21	0	0	66
12	0	0	0	Normoxia	21	0	100	28
12	25	22.4	28.9	Normoxia	21	0	0	282
12	70	62.5	74.8	Normoxia	21	0	0	194
13	0	0	0	Normoxia	21	0	67.15328467	137
13	25	21.4	28.8	Normoxia	21	0	4.295568562	312
13	70	65.6	79.7	Normoxia	21	0	0	1
14	0	0	0	Normoxia	21	0	63.44086022	186
14	25	22.3	26.6	Normoxia	21	0	3.190832361	247
14	70	60.1	73.2	Normoxia	21	0	0	93
15	0	0	0	Normoxia	21	0	88.28125	256
15	25	23.9	24.4	Normoxia	21	0	2.225914905	458
15	70	62.8	71.2	Normoxia	21	0	0	140
16	0	0	0	Normoxia	21	0	96.52173913	115
16	0	0	0	Normoxia	21	0	88.4244373	311
16	25	23.5	26.3	Normoxia	21	0	3.451262874	94
16	25	24.2	26	Normoxia	21	0	2.219873872	341
16	70	57.7	75.3	Normoxia	21	0	0	113
17	0	0	0	Normoxia	21	0	61.92851205	1203
17	0	0	0	Normoxia	21	0	67.64705882	306
18	25	20.9	28.7	Normoxia	21	0	0	479
19	0	0	0	Normoxia	21	0	89.69072165	97
19	0	0	0	Normoxia	21	0	88.8030888	259
19	25	28.6	23.3	Normoxia	21	0	4.212357265	133
19	70	74.7	76.7	Normoxia	21	0	0	200
19	70	73.1	72.2	Normoxia	21	0	0	136
19	70	74.1	72.7	Normoxia	21	0	0	163

Table S3. Continued.

20	0	0	0	Normoxia	21	0	61.9047619	21
20	70	75.3	67.2	Normoxia	21	0	0	31
21	0	0	0	Normoxia	21	0	65.02057613	243
21	0	0	0	Normoxia	21	0	62.06896552	29
21	70	72.6	66.8	Normoxia	21	0	0	199
21	70	65.9	70	Normoxia	21	0	0	431
21	70	68.7	71.6	Normoxia	21	0	0	223
22	0	0	0	Normoxia	21	0	74.50980392	102
22	25	25.3	22.3	Normoxia	21	0	0	23
22	35	40.4	36.6	Normoxia	21	0	1.377931482	487
22	50	54.3	56.2	Normoxia	21	0	0	95
22	70	73	74.2	Normoxia	21	0	0	369
22	70	76.3	70	Normoxia	21	0	0	297
23	0	0	0	Normoxia	21	0	75.75757576	99
23	35	38.5	35.3	Normoxia	21	0	0	231
23	50	45.3	54.7	Normoxia	21	0	0	90
23	70	66.3	77	Normoxia	21	0	0	126
24	0	0	0	Normoxia	21	0	74.16879795	782
25	0	0	0	Normoxia	21	0	78.78787879	33
25	0	0	0	Normoxia	21	0	100	25
25	0	0	0	Normoxia	21	0	94.44444444	18
25	0	0	0	Normoxia	21	0	52.94117647	17
25	25	27	25.3	Normoxia	21	0	0	40
25	35	43.9	36	Normoxia	21	0	0	88
25	50	58.3	50.7	Normoxia	21	0	0	58
25	70	71.3	59.5	Normoxia	21	0	0	23
26	0	0	0	Normoxia	21	0	93.71196755	493
26	25	28.4	24.8	Normoxia	21	0	3.892156476	329
26	25	26.3	25.9	Normoxia	21	0	4.567352233	257
26	35	44	30.1	Normoxia	21	0	0	170
26	50	55.6	59.2	Normoxia	21	0	0	67
27	0	0	0	Normoxia	21	0	55.26315789	38
27	0	0	0	Normoxia	21	0	75.43859649	57
27	0	0	0	Normoxia	21	0	72	25
27	0	0	0	Normoxia	21	0	65	60
27	25	34.7	25.3	Normoxia	21	0	0	35
27	25	27.6	26.6	Normoxia	21	0	0	43
28	0	0	0	Normoxia	21	0	77.94336811	671
28	25	26.4	25.3	Normoxia	21	0	9.449731609	353
28	25	27.9	25.3	Normoxia	21	0	5.37777218	334
28	25	27	25.8	Normoxia	21	0	2.682193989	287
29	0	0	0	Normoxia	21	0	86.8852459	427
29	25	26.8	27	Normoxia	21	0	2.535117613	227
29	35	35.9	37.7	Normoxia	21	0	0.509266989	226
29	35	37.4	35.2	Normoxia	21	0	0.224355438	513
29	50	50.7	53.1	Normoxia	21	0	0	359
29	70	70.2	66.4	Normoxia	21	0	0	215
30	0	0	0	Normoxia	21	0	92.15686275	51
30	35	37.3	32	Normoxia	21	0	0	176
30	50	52.9	53.7	Normoxia	21	0	0	148
31	0	0	0	Normoxia	21	0	53.33333333	75
31	35	32.1	33.2	Normoxia	21	0	0	301
31	50	53.5	42.6	Normoxia	21	0	0	86
31	50	52.1	44.7	Normoxia	21	0	0	84
32	0	0	0	Normoxia	21	0	100	3
32	0	0	0	Normoxia	21	0	100	2
32	50	56.8	48.8	Normoxia	21	0	0	36
32	50	58.1	51.6	Normoxia	21	0	0	23
33	0	0	0	Normoxia	21	0	83.33333333	6

Table S3. Continued.

33	50	69.8	52.7	Normoxia	21	0	0	61
34	0	0	0	Normoxia	21	0	19.23076923	26
34	35	32.6	31.9	Normoxia	21	0	0	60
34	50	53.1	56.4	Normoxia	21	0	0	327
34	50	54.2	59.3	Normoxia	21	0	0	117
34	50	58.9	56.7	Normoxia	21	0	0	179
35	0	0	0	Normoxia	21	0	52.94117647	17
35	0	0	0	Normoxia	21	0	70.27027027	37
35	35	36.7	36	Normoxia	21	0	0	6
35	35	33.4	31.7	Normoxia	21	0	0	48
35	35	40.7	34.7	Normoxia	21	0	0	177
35	50	57.2	52	Normoxia	21	0	0	261
35	50	59.4	52.2	Normoxia	21	0	0	162
36	0	0	0	Normoxia	21	0	88.88888889	63
36	0	0	0	Normoxia	21	0	72.72727273	22
36	0	0	0	Normoxia	21	0	60	15
36	50	53.4	49.2	Normoxia	21	0	0	71
2	0	0	0	Hypoxia	5.54375	15.8875	77.77777778	9
2	0	0	0	Hypoxia	5.9325	15.3125	80	5
2	25	21.9	29	Hypoxia	5.9375	15.3125	7.843137255	17
2	70	63.6	84.1	Hypoxia	6.27375	14.825	0	13
3	0	0	0	Hypoxia	5.715	16.2625	85.18518519	27
3	25	22.3	26.8	Hypoxia	5.44125	16.0625	3.541666667	32
3	70	61.9	76.1	Hypoxia	6.13875	15.9	0	68
4	0	0	0	Hypoxia	5.91125	18.4125	80	30
4	25	22.2	25.8	Hypoxia	5.55875	18.5	0	32
4	70	61.6	77.7	Hypoxia	5.065	13.0625	0	14
5	0	0	0	Hypoxia	5.4	12.4	67.88990826	109
5	25	23.1	25.8	Hypoxia	5.455	10.275	3.246753247	308
5	70	60.7	84.5	Hypoxia	5.63	13.075	0	9
6	70	57.3	73.8	Hypoxia	5.8475	11.625	0	275
6	25	22.3	25.1	Hypoxia	5.66375	13.7375	27.25925926	90
6	70	57	71.3	Hypoxia	5.8375	12.3125	0	72
6	0	0	0	Hypoxia	6.31875	12.65	69.85294118	136
6	0	0	0	Hypoxia	5.555	14.2	50	164
7	0	0	0	Hypoxia	5.5875	14.8125	84.07821229	358
7	0	0	0	Hypoxia	5.885	13.325	83.93665158	442
7	25	23.1	26.1	Hypoxia	5.54625	12.2875	0	19
7	70	64.3	75	Hypoxia	6.2525	13.2125	0	505
8	0	0	0	Hypoxia	5.9275	12.975	86.65207877	457
8	0	0	0	Hypoxia	5.9	12.9625	88.32391714	531
8	25	23.5	24.6	Hypoxia	5.9	13.225	2.877608222	285
8	25	21.9	28.7	Hypoxia	5.63	14.325	8.091143875	362
8	70	60.3	78.5	Hypoxia	6.25125	13.4125	0	279
9	0	0	0	Hypoxia	5.91	14.8	82.15102975	437
9	25	22.2	27.4	Hypoxia	6.21875	15.9625	4.768574521	339
9	25	23.4	28.8	Hypoxia	6.0175	15.8125	17.30133808	307
9	70	62.4	74.8	Hypoxia	6.4625	14.1	0	318
10	0	0	0	Hypoxia	6.25375	13.2375	66.66666667	153
10	0	0	0	Hypoxia	5.9225	13.2625	81.08108108	185
10	25	22.3	26.3	Hypoxia	6.1675	16.225	3.146136503	204
10	70	59.7	82.6	Hypoxia	6.0125	13.875	0	162
10	70	62.4	81.8	Hypoxia	5.77	13.9625	0	31
11	0	0	0	Hypoxia	5.77	15.55	93.1372549	612
11	25	23.6	29.9	Hypoxia	5.74	17.9125	0.364269876	287
11	70	62.3	84.6	Hypoxia	6.37625	16.25	0	66
11	70	59.2	79.5	Hypoxia	6.01375	17.0375	0	336
12	0	0	0	Hypoxia	5.5925	14.625	90.33457249	538
12	0	0	0	Hypoxia	5.91125	14.8625	72.97297297	37
12	25	26.2	27.5	Hypoxia	6.29625	14.475	0.934579439	107

Table S3. Continued.

12	25	25.8	24.5	Hypoxia	5.835	14.85	0	100
12	70	71.6	73.1	Hypoxia	6.2875	15.1875	0	77
12	70	65.8	72.8	Hypoxia	5.89125	12.4875	0	148
13	25	24.1	26.8	Hypoxia	6.1375	13.725	0	1
13	70	59.9	74.6	Hypoxia	5.7375	19.3	0	657
14	25	21.4	27.8	Hypoxia	5.75875	16.25	5.254237288	360
14	0	0	0	Hypoxia	5.9675	14.0125	100	1
14	70	65.4	73.8	Hypoxia	5.70375	15.6875	0	242
14	70	64.4	71.1	Hypoxia	5.8375	13.95	0	217
15	0	0	0	Hypoxia	5.64	15.6	96.14035088	285
15	25	23.1	27.6	Hypoxia	5.68375	12.7125	0.891923908	254
15	25	22.6	27.7	Hypoxia	5.5275	15.425	0	256
15	70	62.7	71.2	Hypoxia	5.51375	17.075	0	246
16	0	0	0	Hypoxia	7.415	13.525	84.61538462	52
16	25	23.6	28.9	Hypoxia	5.3175	15.9625	11.51782403	507
16	25	25.6	24.4	Hypoxia	7.53375	12.4	11.26453855	96
16	70	76.7	76.4	Hypoxia	6.6025	14.15	0	66
16	70	73.4	75.8	Hypoxia	6.8525	13.4375	0	22
17	0	0	0	Hypoxia	5.555	15.0875	83.50515464	388
17	70	70.5	67.4	Hypoxia	4.18625	18.7	0	304
19	0	0	0	Hypoxia	6.49875	15.5375	89.86486486	148
19	25	25.4	23.8	Hypoxia	5.2875	15.2	25.85739306	78
22	0	0	0	Hypoxia	5.33375	14.9875	51.02040816	49
22	0	0	0	Hypoxia	5.44125	15.0625	64.0625	64
22	25	28.3	25.9	Hypoxia	5.32625	16.8625	8.603238866	78
22	50	58.5	56	Hypoxia	5.0325	15.1125	0	94
22	70	73.4	70.2	Hypoxia	5.1875	15.75	0	442
23	0	0	0	Hypoxia	5.335	18.6875	59.45945946	37
23	35	38.6	39.3	Hypoxia	4.6675	13.55	0	489
23	50	58.3	59.1	Hypoxia	5.2275	18.0125	0	225
24	0	0	0	Hypoxia	5.22	15.55	70.93235832	547
24	25	25.9	25.3	Hypoxia	4.69	16.375	6.461943899	772
24	25	25.6	25.3	Hypoxia	5.5425	14.1375	0.701313842	769
24	35	37.3	38.9	Hypoxia	5.88125	14.6375	0	616
24	35	36.3	38.5	Hypoxia	4.665	20.775	0	954
24	50	58.5	55	Hypoxia	5.52	17.075	0	46
24	50	60.3	52.6	Hypoxia	4.86125	19.7375	0	21
24	70	77	79.6	Hypoxia	4.48875	14.925	0	64
24	70	71.7	73.9	Hypoxia	5.81875	15	0	54
25	0	0	0	Hypoxia	6.6525	13.8625	73.33333333	15
25	25	28.3	27.1	Hypoxia	6.04	14.4625	0	47
25	35	43.8	44.8	Hypoxia	4.84875	16.375	0	13
25	25	26.7	25.9	Hypoxia	5.1525	15.675	1.039272201	354
25	35	42.2	43.8	Hypoxia	4.81625	16.8125	0	161
25	50	55.7	53.9	Hypoxia	4.76	16.1125	0	198
25	50	57.6	59.6	Hypoxia	5.6325	15.5625	0	61
25	50	56.8	55.2	Hypoxia	5.20625	15.5375	0	167
25	70	77.3	72.8	Hypoxia	5.49375	13.8875	0	90
25	70	79.9	70.8	Hypoxia	5.41375	16.7375	0	131
25	70	70.1	74.9	Hypoxia	6.40875	14.4	0	94
25	70	84.7	73.6	Hypoxia	5.32625	16	0	141
26	0	0	0	Hypoxia	4.6585714	22.4	96.50924025	487
26	25	29.6	25.4	Hypoxia	4.9028571	18.342857	16.45307703	227
26	25	26.5	25.3	Hypoxia	4.4228571	19.871429	1.83982684	348
26	35	42.9	35.5	Hypoxia	4.33	20.214286	0	366
26	50	56.2	48.9	Hypoxia	5.16	14.985714	0	160
27	0	0	0	Hypoxia	4.9742857	16.5	82.69230769	52
28	0	0	0	Hypoxia	4.345	19.3875	100	605
28	0	0	0	Hypoxia	4.63875	20.5375	98.50746269	201
28	25	27.8	25.4	Hypoxia	4.277625	19.7875	6.301487189	509
28	25	26.5	25.3	Hypoxia	4.25875	19.7	7.702710944	533
28	25	27.5	25.3	Hypoxia	4.79	20.025	6.109441865	315
29	0	0	0	Hypoxia	5.8085714	18.328571	66.97247706	109

Table S3. Continued.

29	25	28.2	24.6	Hypoxia	4.7557143	24.028571	2.16416707	585
29	35	40.4	34.5	Hypoxia	4.9571429	18.171429	0	265
29	35	41.7	36.4	Hypoxia	4.4814286	18.714286	0	321
29	50	52.6	55.7	Hypoxia	4.5142857	14.485714	0	784
29	50	55.4	52.4	Hypoxia	4.2157143	20.271429	0	383
29	70	70.4	79.7	Hypoxia	4.3414286	19.714286	0	321
29	70	66.3	71.5	Hypoxia	4.7014286	12.757143	0	447
30	0	0	0	Hypoxia	5.4614286	18.057143	100	48
30	35	36.6	34.8	Hypoxia	5.9428571	17.928571	0	50
30	35	37	32	Hypoxia	5.1628571	16.642857	2.5	40
30	50	58.2	48.1	Hypoxia	4.9914286	13.271429	0	323
1	70	61.8	73.1	Severe hypoxia	0.21825	24.0375	0	109
1	0	0	0	Severe hypoxia	0	27.7375	60.83333333	240
1	0	0	0	Severe hypoxia	0.150875	20.7625	45.57823129	147
1	25	22.9	28.5	Severe hypoxia	0.095375	26.5875	24.70672573	531
1	25	22.5	24.5	Severe hypoxia	0.366625	22.075	44.09839113	413
1	70	60.9	74.4	Severe hypoxia	0.19075	21.5125	0	429
2	0	0	0	Severe hypoxia	0.08825	21.15	7.272727273	55
2	25	22.3	28.1	Severe hypoxia	0.035875	22.75	22.22222222	60
3	0	0	0	Severe hypoxia	0.2355	21.025	66.66666667	12
3	25	22.3	26.8	Severe hypoxia	0.206125	21.275	17	20
3	70	63.5	82.7	Severe hypoxia	0.179375	20.9625	0	5
3	70	61.7	75.5	Severe hypoxia	0.143875	21.825	0	33
4	0	0	0	Severe hypoxia	0.198625	25.225	68.75	16
4	25	22.7	27.4	Severe hypoxia	0.19725	23.25	37.5	4
4	70	61.9	78.3	Severe hypoxia	0.041625	30.1	0	65
5	0	0	0	Severe hypoxia	0.152375	21.85	50	2
5	0	0	0	Severe hypoxia	0.2945	21.4375	0	1
5	25	21.7	25.9	Severe hypoxia	0.033625	25.8375	75	10
5	70	63	84.6	Severe hypoxia	0.03375	27.025	0	184
6	0	0	0	Severe hypoxia	0.017	24.55	36.95652174	46
6	25	25.1	26.4	Severe hypoxia	0.196	22.7625	0	57
7	25	22.7	26.2	Severe hypoxia	0.053375	21.4125	2.718263521	402
7	70	60.9	74.1	Severe hypoxia	0.202125	23.95	0	89
7	0	0	0	Severe hypoxia	0.1205	23.3875	100	1
8	0	0	0	Severe hypoxia	0.058625	22.375	76.05633803	426
8	25	23.1	24.7	Severe hypoxia	0.0985	21.8875	63.73491124	250
8	70	59.8	77.8	Severe hypoxia	0.169875	20.725	0	504
9	0	0	0	Severe hypoxia	0.034875	23.925	85.51401869	214
9	25	22.4	26.8	Severe hypoxia	0.136125	21.675	46.42512794	97
9	70	67.6	78.9	Severe hypoxia	0.105875	22.8375	0	142
10	0	0	0	Severe hypoxia	0.255625	21.925	60.86956522	23
10	25	22	24.3	Severe hypoxia	0.163875	21.1125	59.83686438	261
10	70	59	77.9	Severe hypoxia	0.077625	22.2625	0	257
11	0	0	0	Severe hypoxia	0.138375	22.7375	88.06818182	176
11	25	23.9	26.3	Severe hypoxia	0.161125	22.1375	36.36363636	115
11	70	61.3	77.3	Severe hypoxia	0.09125	22.8375	0	116
12	0	0	0	Severe hypoxia	0.16875	22.2	56.36363636	165
12	25	23.2	31.1	Severe hypoxia	0.10225	23.4	0.5	200
12	70	71.2	76.9	Severe hypoxia	0.184375	21.3	0	124
13	0	0	0	Severe hypoxia	0.208625	21.4875	58.75	160
13	0	0	0	Severe hypoxia	0.190125	22.075	65.70512821	312
13	25	24.1	29.7	Severe hypoxia	0.227	21.4625	16.54589372	54
13	70	66.6	80.5	Severe hypoxia	0.294375	21.375	0	23
14	0	0	0	Severe hypoxia	0.2595	22.3625	41.66666667	48
14	25	25.2	31.7	Severe hypoxia	0.263125	22.65	45.03631961	91
15	0	0	0	Severe hypoxia	0.22575	21.65	91.85803758	479

Table S3. Continued.

15	0	0	0	Severe hypoxia	0.05725	24.55	82.0754717	212
15	25	25	27.1	Severe hypoxia	0.259	21.725	12.11109256	159
15	70	67.5	76	Severe hypoxia	0.0615	24.8125	0	107
15	70	66.6	71	Severe hypoxia	0.1795	22.95	0	56
16	0	0	0	Severe hypoxia	0.998	20.457143	95.69536424	302
16	0	0	0	Severe hypoxia	0.9478571	20.214286	55.72916667	192
16	25	24.5	29.6	Severe hypoxia	0.9692857	19.885714	33.98672201	35
16	25	23.6	31	Severe hypoxia	0.923	21.157143	57.8420956	817
16	70	64.9	76.8	Severe hypoxia	0.9664286	19.671429	0	90
17	0	0	0	Severe hypoxia	0.927625	20.75	96.17486339	366
17	25	24.5	23.8	Severe hypoxia	0.951875	20.2125	34.79711995	621
17	25	26.6	27	Severe hypoxia	1.0705714	20.042857	40.61844265	209
17	70	66.2	75.7	Severe hypoxia	0.376125	24.1875	0	84
17	70	75.7	69.9	Severe hypoxia	0.602875	23.1	0	881
18	70	70.8	74.8	Severe hypoxia	0.8345	20.8125	0	257
18	70	69.7	71.9	Severe hypoxia	1.227375	19.6375	0	172
19	0	0	0	Severe hypoxia	0.471875	21.1375	93.75	224
19	0	0	0	Severe hypoxia	0.42075	20.9375	85.69157393	629
19	70	72.1	69	Severe hypoxia	0.07025	22.4625	0	149
19	70	72.7	77.6	Severe hypoxia	0.2375	21.425	0	188
20	70	79.4	77.4	Severe hypoxia	0.587	20.125	0	67
20	70	78.1	74.6	Severe hypoxia	0.433875	22.3125	0	48
21	0	0	0	Severe hypoxia	0.520625	20.425	62.5	72
21	70	69.4	56.6	Severe hypoxia	0.44125	20.4375	0	510
21	70	70.8	73.1	Severe hypoxia	0.378125	20.6875	0	614
22	0	0	0	Severe hypoxia	0.5325	20.725	77.77777778	45
22	25	26.1	24.1	Severe hypoxia	0.378	20.6875	63.27067669	280
22	35	36.7	37.7	Severe hypoxia	0.425375	20.6	8.07748538	432
22	50	54.6	44.1	Severe hypoxia	0.3265	21.2125	0	214
22	70	82.2	72	Severe hypoxia	0.50425	20.8125	0	304
23	0	0	0	Severe hypoxia	0.328375	24.2375	62.96296296	81
23	35	38.7	36.7	Severe hypoxia	0.202125	23.725	13.03291139	395
23	50	51.9	49	Severe hypoxia	0.557625	23.5625	0	56
23	70	70.2	65.3	Severe hypoxia	0.676875	21.55	0	24
24	70	88.4	79.7	Severe hypoxia	0.481875	19.125	0	744
25	0	0	0	Severe hypoxia	0.510625	19.925	62.06896552	29
25	70	83.9	75.6	Severe hypoxia	0.563125	19.65	0	25
26	0	0	0	Severe hypoxia	0.121	25.971429	100	274
26	25	39.3	25.4	Severe hypoxia	0.3437143	23.757143	47.20707961	486
26	25	30	25.4	Severe hypoxia	0.483	24.8	24.50376784	135
27	0	0	0	Severe hypoxia	0.1637143	22.757143	96	50
27	25	28.7	31.2	Severe hypoxia	0.479	21.9	73.65775359	71
27	25	36	33.6	Severe hypoxia	0.1298571	25.628571	56.03250541	160
28	0	0	0	Severe hypoxia	0.118625	26.975	93.36569579	618
28	0	0	0	Severe hypoxia	0.408625	24.3375	99.08256881	218
28	25	27.3	26.4	Severe hypoxia	0.158875	25.9875	32.46731962	980
28	25	33.1	34.2	Severe hypoxia	0.329625	20.9125	75.50568582	627
29	0	0	0	Severe hypoxia	0.1084286	28.171429	89.38053097	226
29	70	73.4	74.5	Severe hypoxia	0.2831429	20.328571	0	392
30	0	0	0	Severe hypoxia	0.0232857	24.485714	83.17307692	208
30	35	46.5	47.6	Severe hypoxia	0.5322857	20.2	2.836879433	153
30	35	31.3	48.4	Severe hypoxia	0.4722857	23.128571	36.17021277	18
30	70	77.2	72.4	Severe hypoxia	0.1172857	22.385714	0	104
30	50	56.4	52.9	Severe hypoxia	0.5328571	24.428571	0	12
30	50	54.9	39.9	Severe hypoxia	0.4458571	27.314286	0	25
31	0	0	0	Severe hypoxia	0.528625	21.525	83.33333333	6
31	35	35.3	30.7	Severe hypoxia	0.492375	21.4375	0	79
31	35	38.4	34	Severe hypoxia	0.387375	22.425	0	204
31	50	53.6	45.8	Severe hypoxia	0.273625	22.8875	0	45
32	0	0	0	Severe hypoxia	0.544875	17.88875	78.125	32
32	35	49.1	36.3	Severe hypoxia	0.384125	20.8625	0	30
32	50	56.6	56.7	Severe hypoxia	0.653	21.325	0	85
32	50	56.2	57.8	Severe hypoxia	0.45925	21.025	0	35

Table S3. Continued.

33	35	42.9	40.2	Severe hypoxia	0.434875	21.1625	0	32
33	50	54.8	55	Severe hypoxia	0.434875	22.55	0	82
33	50	59.1	54.4	Severe hypoxia	0.238625	20.8125	0	367
33	50	62.2	64.1	Severe hypoxia	0.4435	22.35	0	225
33	50	57.8	53.4	Severe hypoxia	0.238625	20.8125	0	14
34	0	0	0	Severe hypoxia	0.385875	20.9875	53.125	32
34	35	40.6	38.5	Severe hypoxia	0.39925	20.75	6.265060241	83
34	35	39.9	42.7	Severe hypoxia	0.251	21.35	0	274
34	35	40.7	31.1	Severe hypoxia	0.401875	21.25	0	952
34	50	66.5	58.1	Severe hypoxia	0.144125	21.35	0	139
34	50	60	51.8	Severe hypoxia	0.589	20.5375	0	292
34	50	62.2	51.1	Severe hypoxia	0.444375	22.2625	0	263
34	50	58.4	51.8	Severe hypoxia	0.592625	21.7625	0	95
35	0	0	0	Severe hypoxia	0.417625	21.3125	58.92857143	56
35	0	0	0	Severe hypoxia	0.514125	20.675	81.25	16
35	50	55	53.2	Severe hypoxia	0.172625	24.8125	0	252
36	0	0	0	Severe hypoxia	0.383125	20.65	71.875	64
36	35	38.6	42.2	Severe hypoxia	0.4395	20.675	0	49
36	50	59.8	52.8	Severe hypoxia	0.4715	20.55	0	117

Table S4. Experimental data for *Anastrepha ludens*

Block	Nominal dose	Absorbed dose: bottom	Absorbed dose: top	Atmospheric conditions	O_2(%)	CO_2 (%)	Adult emergence (corrected)	No. insects treated
1	0	0	0	Normoxia	21	0	98.76543	81
1	0	0	0	Normoxia	21	0	83.33333	6
1	25	23.3	25.2	Normoxia	21	0	0	6
1	70	87.4	67.8	Normoxia	21	0	0	2
2	70	63.3	76.5	Normoxia	21	0	0	30
2	25	21.8	27.2	Normoxia	21	0	0	2
2	70	65.3	81.6	Normoxia	21	0	0	8
3	25	22.8	24	Normoxia	21	0	0	3
4	0	0	0	Normoxia	21	0	62.5	8
4	70	62.5	82	Normoxia	21	0	0	19
5	25	21.5	24.3	Normoxia	21	0	0	9
5	70	65.8	85.4	Normoxia	21	0	0	6
6	0	0	0	Normoxia	21	0	97.82609	46
6	25	23.4	25.1	Normoxia	21	0	3.484848	176
6	25	20.8	30.9	Normoxia	21	0	7.163822	371
6	70	55.9	68.5	Normoxia	21	0	0	7
6	70	55.7	79.7	Normoxia	21	0	0	90
7	0	0	0	Normoxia	21	0	93.88298	376
7	0	0	0	Normoxia	21	0	94.44444	162
7	25	22	27.4	Normoxia	21	0	2.003736	583
7	25	23.9	29.2	Normoxia	21	0	1.259265	506
7	70	64.6	75.3	Normoxia	21	0	0	264
7	70	65.9	74.3	Normoxia	21	0	0	100
8	0	0	0	Normoxia	21	0	47.05882	34
8	25	21	23.7	Normoxia	21	0	0	46
8	70	62.8	74.7	Normoxia	21	0	0	219
8	70	65	63.1	Normoxia	21	0	0	5
9	0	0	0	Normoxia	21	0	79.13669	139
9	25	23.6	31.7	Normoxia	21	0	0	82
9	70	76.1	80.2	Normoxia	21	0	0	38
9	70	68.8	72.8	Normoxia	21	0	0	230
10	0	0	0	Normoxia	21	0	74.84277	159
10	70	71.5	71.9	Normoxia	21	0	0	109
10	70	74.7	72.9	Normoxia	21	0	0	337
11	0	0	0	Normoxia	21	0	88.41463	164
11	50	52	49.3	Normoxia	21	0	0	1
11	35	38.1	36.4	Normoxia	21	0	0	102
11	35	38.3	35.7	Normoxia	21	0	0	233
11	50	58.2	48.4	Normoxia	21	0	0	99
12	0	0	0	Normoxia	21	0	33.58779	262
12	70	73.9	72.4	Normoxia	21	0	0	87
13	0	0	0	Normoxia	21	0	80.39216	102
13	0	0	0	Normoxia	21	0	52.57732	194
13	0	0	0	Normoxia	21	0	50	84
13	35	39.9	35.3	Normoxia	21	0	0	28
14	0	0	0	Normoxia	21	0	50	10
14	0	0	0	Normoxia	21	0	33.33333	12
14	0	0	0	Normoxia	21	0	53.33333	15
14	0	0	0	Normoxia	21	0	68	25
14	50	54.8	43.6	Normoxia	21	0	0	9
15	0	0	0	Normoxia	21	0	73.33333	30
15	0	0	0	Normoxia	21	0	42.69663	89
15	0	0	0	Normoxia	21	0	76.19048	63
15	50	48.8	43.2	Normoxia	21	0	0	75
16	35	31.7	25.7	Normoxia	21	0	0	43
17	0	0	0	Normoxia	21	0	52.27273	132

Table S4. Continued.

17	35	29.9	29.7	Normoxia	21	0	0	88
17	50	44	36.8	Normoxia	21	0	0	126
17	50	57.4	56.3	Normoxia	21	0	0	17
18	0	0	0	Normoxia	21	0	39.53488	129
18	0	0	0	Normoxia	21	0	37.5	24
18	0	0	0	Normoxia	21	0	64.44444	45
18	25	25.5	26.5	Normoxia	21	0	2.409604	176
18	25	25.4	25.9	Normoxia	21	0	4.609676	46
18	25	25.7	26	Normoxia	21	0	0	75
18	25	25.4	28.4	Normoxia	21	0	0	16
18	35	32.3	30.8	Normoxia	21	0	0	108
18	35	33.4	36	Normoxia	21	0	0	12
18	35	33.4	28.4	Normoxia	21	0	0	13
18	35	33.9	32.7	Normoxia	21	0	0	12
18	35	34.3	29.7	Normoxia	21	0	0	153
19	0	0	0	Normoxia	21	0	80.90909	110
19	0	0	0	Normoxia	21	0	97.92746	193
19	25	25.4	25.3	Normoxia	21	0	1.471499	456
19	25	25.7	25.4	Normoxia	21	0	0.458336	488
19	25	25.6	25.5	Normoxia	21	0	3.458782	194
19	25	25.5	26.3	Normoxia	21	0	0.251312	445
19	35	36.2	42.4	Normoxia	21	0	0	70
19	35	31.6	29.2	Normoxia	21	0	0	24
19	35	34.6	32.8	Normoxia	21	0	0	144
19	35	36.2	31.8	Normoxia	21	0	0	466
20	0	0	0	Normoxia	21	0	78.57143	70
20	0	0	0	Normoxia	21	0	94.11765	34
20	0	0	0	Normoxia	21	0	14.28571	7
20	0	0	0	Normoxia	21	0	77.61194	67
20	25	25.6	25.6	Normoxia	21	0	0	131
20	25	29.7	33	Normoxia	21	0	0	16
20	25	25.8	25.4	Normoxia	21	0	0	82
20	35	33.6	30.7	Normoxia	21	0	0	220
20	35	34.5	32.1	Normoxia	21	0	0	70
20	35	32.3	29.3	Normoxia	21	0	0	170
20	35	31.6	29.2	Normoxia	21	0	0	285
20	35	36.1	31.4	Normoxia	21	0	0	189
21	0	0	0	Normoxia	21	0	80.18868	106
21	0	0	0	Normoxia	21	0	42.03822	471
21	0	0	0	Normoxia	21	0	95.91837	98
21	25	25.8	25.4	Normoxia	21	0	0	279
21	0	0	0	Normoxia	21	0	39.52569	253
21	50	52.8	67.5	Normoxia	21	0	0	172
22	0	0	0	Normoxia	21	0	79.18919	370
22	0	0	0	Normoxia	21	0	92.16867	166
22	35	30.3	35.8	Normoxia	21	0	1.161341	402
22	25	28.6	25.3	Normoxia	21	0	1.087405	322
22	25	26.3	25.3	Normoxia	21	0	12.21434	258
22	25	26.3	26.2	Normoxia	21	0	0.235787	495
22	25	25.7	25.3	Normoxia	21	0	3.934207	356
22	50	53.2	55.9	Normoxia	21	0	0	85
23	0	0	0	Normoxia	21	0	48.3871	248
23	25	25.5	25.3	Normoxia	21	0	0	200
23	25	27.9	25.4	Normoxia	21	0	2.48	502
23	35	33	28.6	Normoxia	21	0	0	249
23	50	52.3	49.2	Normoxia	21	0	0	97
23	70	70.9	72.9	Normoxia	21	0	0	371
24	0	0	0	Normoxia	21	0	20	15
24	25	27.9	25.4	Normoxia	21	0	6.782946	516

Table S4. Continued.

25	0	0	0	Normoxia	21	0	84.63612	371
25	25	25.9	25.8	Normoxia	21	0	0.868771	136
25	25	26.6	29.8	Normoxia	21	0	0.312574	378
25	35	39.1	34.2	Normoxia	21	0	0.772241	306
25	50	60.1	51.2	Normoxia	21	0	0	138
25	70	72.2	79.8	Normoxia	21	0	0	35
26	0	0	0	Normoxia	21	0	95.2381	21
26	0	0	0	Normoxia	21	0	28.75	80
26	0	0	0	Normoxia	21	0	93.54839	31
26	25	25.7	25.5	Normoxia	21	0	3.163025	218
26	25	25.6	26	Normoxia	21	0	0.985056	280
26	35	34.6	34.4	Normoxia	21	0	0	90
26	50	55.2	50.9	Normoxia	21	0	0	25
26	50	54.1	52.9	Normoxia	21	0	0	99
27	0	0	0	Normoxia	21	0	92.71709	357
27	0	0	0	Normoxia	21	0	93.47826	184
27	25	26.7	25.8	Normoxia	21	0	15.24805	317
27	25	26.9	26	Normoxia	21	0	1.866208	111
27	25	27	25.8	Normoxia	21	0	3.579249	463
27	50	53.8	48.5	Normoxia	21	0	0	29
28	0	0	0	Normoxia	21	0	80.59701	67
28	0	0	0	Normoxia	21	0	40	5
28	0	0	0	Normoxia	21	0	75.55556	45
28	25	27.1	26.2	Normoxia	21	0	0	43
28	70	79.4	71	Normoxia	21	0	0	23
29	0	0	0	Normoxia	21	0	88.57143	35
29	0	0	0	Normoxia	21	0	95.74468	47
29	25	25.3	27.1	Normoxia	21	0	0	14
30	50	54.8	43.9	Normoxia	21	0	0	117
30	50	58.3	45.5	Normoxia	21	0	0	5
30	50	57.5	50.7	Normoxia	21	0	0	356
30	70	74.4	68.5	Normoxia	21	0	0	10
31	0	0	0	Normoxia	21	0	36.84211	19
31	50	59.7	52.8	Normoxia	21	0	0	18
31	0	0	0	Normoxia	21	0	71.875	64
31	0	0	0	Normoxia	21	0	66.66667	12
31	35	35.1	34.2	Normoxia	21	0	0	462
31	50	46	56.8	Normoxia	21	0	0	617
32	0	0	0	Normoxia	21	0	78.57143	42
32	50	58.8	61.7	Normoxia	21	0	0	8
32	50	62	54.2	Normoxia	21	0	0	330
32	50	68.1	53	Normoxia	21	0	0	62
32	50	58	50.6	Normoxia	21	0	0	227
32	0	0	0	Normoxia	21	0	66.66667	6
32	50	58.8	54.6	Normoxia	21	0	0	19
3	0	0	0	Hypoxia	6.13125	15.1	100	19
3	70	63.6	83.8	Hypoxia	5.44125	15.525	0	2
4	0	0	0	Hypoxia	6.29875	14.125	66.66667	6
4	25	20.7	27.7	Hypoxia	5.9125	15.475	0	4
6	0	0	0	Hypoxia	6.13125	14.875	84.61538	299
6	25	20.8	29.2	Hypoxia	5.92875	15.375	10.76023	114
6	25	23.3	24.9	Hypoxia	6.2675	13.2	0.367706	278
6	70	73.9	74.4	Hypoxia	6.41625	12.3375	0	166
6	70	65	77.3	Hypoxia	6.32625	12.575	0	15
7	0	0	0	Hypoxia	5.795	16.125	91.78082	219
7	0	0	0	Hypoxia	5.8875	15.275	88.4058	552
7	25	23.8	28.4	Hypoxia	5.8875	14.3625	15.29972	236
7	25	29.9	24.2	Hypoxia	5.83	13.575	0	354
7	70	65.2	78.7	Hypoxia	5.40125	17.4375	0	199
7	70	67.6	76.6	Hypoxia	5.775	14.2375	0	635
8	0	0	0	Hypoxia	6.1075	15.6125	81.25	16
8	25	22.6	26.6	Hypoxia	5.655	13.8375	0	11
8	70	62.9	71.8	Hypoxia	5.9475	13.025	0	68

Table S4. Continued.

9	0	0	0	Hypoxia	6.18875	15.3	61.53846	13
9	25	24.6	30.2	Hypoxia	5.97625	13.9	0	1
9	70	66.5	75.6	Hypoxia	5.7875	13.8875	0	1
10	0	0	0	Hypoxia	5.53	15.4375	70.35176	199
10	25	31	23.6	Hypoxia	6.12625	14.2875	1.56273	171
10	70	72.1	74.1	Hypoxia	4.95125	16.1	0	213
12	35	37.7	38.8	Hypoxia	5.0475	19.9375	0	784
12	50	63.3	57.5	Hypoxia	5.41375	15.55	0	552
12	70	88.2	70.7	Hypoxia	5.61375	15.8625	0	422
13	70	73.3	73.4	Hypoxia	5.3525	16.4125	0	3
13	25	27.8	26	Hypoxia	4.87	16.95	2.277247	72
13	50	49	50.4	Hypoxia	5.2725	15.9625	0	109
23	0	0	0	Hypoxia	5	15.075	72.8	265
23	25	27.6	25.3	Hypoxia	4.475	16.2875	1.033333	381
23	25	31	29.8	Hypoxia	5.14	14.1875	79.15333	394
23	25	28.4	25.3	Hypoxia	4.6275	14.975	79.11458	256
23	35	41.9	34.4	Hypoxia	4.94625	13.5375	0	22
23	50	53.1	51.3	Hypoxia	4.625	16.3375	0	417
24	0	0	0	Hypoxia	4.355	15.15	96.10778	334
24	25	26.7	26	Hypoxia	4.79375	13.7625	0	232
24	35	36.6	34.1	Hypoxia	5.72125	12.7375	0	485
24	50	57.8	46.7	Hypoxia	4.8875	13.1625	0	490
24	70	83.2	77.1	Hypoxia	5.04125	14.1	0	208
25	0	0	0	Hypoxia	4.64625	17.2875	88.52814	462
25	25	26.9	26.4	Hypoxia	4.5275	16.7875	1.618532	292
25	25	27.1	32.1	Hypoxia	4.94875	15.6125	5.52403	385
25	25	29.3	26.3	Hypoxia	5.5525	15.175	1.074117	440
25	35	38.4	35	Hypoxia	5.095	15.75	0	235
25	50	57.7	53.2	Hypoxia	4.63375	16.7	0	626
26	0	0	0	Hypoxia	4.3375	16.5625	78.82353	170
26	0	0	0	Hypoxia	5.695	15.275	96.22642	53
26	25	27.9	25.4	Hypoxia	5.01625	16.1375	8.728348	79
26	25	27.3	31.1	Hypoxia	4.8125	17.3125	4.925282	140
26	35	45.2	41.5	Hypoxia	5.14375	15.6625	0	16
26	50	57.6	53.5	Hypoxia	5.045	14.7875	0	71
27	0	0	0	Hypoxia	4.87	16.5625	69.17148	519
27	0	0	0	Hypoxia	4.3975	16.7125	84.9642	419
27	25	30.3	25.9	Hypoxia	4.66375	16.3375	1.599607	259
27	25	26.8	25.9	Hypoxia	4.99	15.4125	1.70493	486
27	0	0	0	Hypoxia	4.52	16.3375	74.21384	159
27	35	39.8	41.8	Hypoxia	5.06875	15.4	0	367
27	35	45.7	43.8	Hypoxia	5.2375	14.9	0	340
27	50	58.2	53.5	Hypoxia	5.07875	16.6125	0	175
27	50	52.8	58.1	Hypoxia	4.81125	19.0875	0	158
27	70	75.5	74	Hypoxia	4.5775	15.575	0	200
27	70	81.6	77	Hypoxia	4.5725	15.725	0	286
28	25	26.2	25.4	Hypoxia	4.33625	17.5875	0	25
28	25	27.1	26.5	Hypoxia	4.41	20.175	0	58
28	35	38.3	35.2	Hypoxia	4.6	19.7375	0	140
28	35	38.6	40.7	Hypoxia	5.085	16.95	0	154
28	50	63.2	48.4	Hypoxia	4.7825	16.9125	0	105
28	50	58.5	52.3	Hypoxia	4.44	18.1	0	29
28	70	81.6	70.4	Hypoxia	4.45375	17.15	0	50
29	0	0	0	Hypoxia	4.69375	18.7	82.75862	29
29	25	27.6	25.6	Hypoxia	5.93	16.6625	0	31
29	25	29	28.6	Hypoxia	4.90625	18.525	6.643423	49
29	35	41.3	34.6	Hypoxia	4.5175	19.0125	1.528299	71
29	35	48.6	41.4	Hypoxia	4.68625	18.2125	0	152
29	50	58.2	50.3	Hypoxia	4.62375	16.1625	0	27
31	0	0	0	Hypoxia	5.15375	16.6	75	24
31	25	25.9	24.2	Hypoxia	5.13875	15.9875	11.13276	791

Table S4. Continued.

31	35	37.1	31.6	Hypoxia	5.55125	19.35	0	98
31	50	54.3	53.6	Hypoxia	4.9875	12.2	0	237
1	0	0	0	Severe hypoxia	0.1565	22.0875	31.57895	38
1	25	22.5	25.9	Severe hypoxia	0.131125	22.0125	69.89214	11
1	70	62	77.3	Severe hypoxia	0.12725	22.85	0	52
2	25	23.1	26.5	Severe hypoxia	0.143625	21.9875	0	1
2	25	21.6	27.8	Severe hypoxia	0.085125	22.25	83.33333	6
3	25	22	26.4	Severe hypoxia	0.141875	22.9625	26.15385	65
3	70	60.4	79.2	Severe hypoxia	0.2545	20.8875	0	47
4	25	23.3	28.3	Severe hypoxia	0.141875	21.35	32	10
4	25	22.1	29.4	Severe hypoxia	0.21725	20.975	0	1
5	70	59.2	80.7	Severe hypoxia	0.08575	21.25	0	6
6	0	0	0	Severe hypoxia	0.01125	23.875	70.71429	140
6	25	24.6	26.4	Severe hypoxia	0.151875	22.4	77.18821	147
6	25	24.2	29.8	Severe hypoxia	0.229875	21.325	51.11111	4
6	70	57.3	80.2	Severe hypoxia	0.194	22.2625	0	1
6	70	70	74.8	Severe hypoxia	0.083875	24.775	0	38
7	0	0	0	Severe hypoxia	0.1055	21.8375	88.18898	127
7	25	29.3	24	Severe hypoxia	0.121125	21.675	26.54951	120
7	70	74.8	76.2	Severe hypoxia	0.271125	22.1625	0	10
7	70	72.9	75.9	Severe hypoxia	0.2225	22.0375	0	2
8	70	64.3	74.2	Severe hypoxia	0.293	20.85	0	161
8	25	23.5	25.2	Severe hypoxia	0.291375	20.825	0	5
9	25	26.8	29.6	Severe hypoxia	0.203	21.45	13.71388	258
10	0	0	0	Severe hypoxia	0.693375	22.025	47.05882	340
10	25	25.6	26.4	Severe hypoxia	1.693625	20.2125	2.168169	493
10	25	27.9	24.4	Severe hypoxia	1.285375	19.58125	22.98726	93
10	25	26.9	33.9	Severe hypoxia	0.793125	20.1625	32.06723	75
10	70	72	79.8	Severe hypoxia	1.344125	18.85	0	162
10	70	72.4	69.6	Severe hypoxia	0.9565	20.1375	0	86
10	70	72.1	72.7	Severe hypoxia	1.058125	20.5125	0	113
12	0	0	0	Severe hypoxia	0.746875	20.575	0	13
12	35	37.4	37.9	Severe hypoxia	0.546375	21.75	0	90
12	50	51.3	53.5	Severe hypoxia	0.4975	20.2375	0	406
13	50	51.5	51	Severe hypoxia	0.782125	20.8125	0	1
13	0	0	0	Severe hypoxia	0.8405	20.7625	59.31034	145
13	35	30.2	38.5	Severe hypoxia	0.832375	20.7125	0	30
13	25	28.8	21.6	Severe hypoxia	0.617	20.95	9.181859	125
13	70	71.4	70.6	Severe hypoxia	0.731	21.2125	0	227
13	70	67.7	74	Severe hypoxia	0.683625	20.575	0	216
14	0	0	0	Severe hypoxia	0.24325	21.1	66.66667	105
14	50	56.5	48	Severe hypoxia	0.191625	23.375	0	46
14	70	80.2	66.8	Severe hypoxia	0.173625	22.9625	0	40
15	0	0	0	Severe hypoxia	0.1575	20.8625	48.85845	219
15	0	0	0	Severe hypoxia	0.143625	21.95	56.25	160
15	0	0	0	Severe hypoxia	0.1205	22.3875	72.5	40
15	50	48.4	44.4	Severe hypoxia	0.19075	21.975	0	77
15	50	61.4	56.5	Severe hypoxia	0.1205	21.15	0	77
15	70	76.5	79	Severe hypoxia	0.149	20.375	0	100
15	70	78.1	67.7	Severe hypoxia	0.19425	20.9	0	75
16	25	25.8	25.5	Severe hypoxia	0.189625	21.4	89.5112	68
16	35	30.4	27.4	Severe hypoxia	0.1925	21.2875	14.63164	32
17	0	0	0	Severe hypoxia	0.1535	20.5125	84.21053	190
17	0	0	0	Severe hypoxia	0.159375	21.3625	72.72727	88
17	0	0	0	Severe hypoxia	0.373125	20.825	82.25806	62
17	35	30.1	34.5	Severe hypoxia	0.19075	20.4875	0	30
17	35	31.8	40.9	Severe hypoxia	0.18675	20.4875	2.224469	258
17	35	41.6	29.7	Severe hypoxia	0.1165	20.675	0	174
17	50	48.1	47.2	Severe hypoxia	0.202125	20.5375	0	40
17	50	51.2	55	Severe hypoxia	0.2425	21.025	0	67
17	50	61.6	54.1	Severe hypoxia	0.18125	20.225	0	97
17	50	58.7	53.8	Severe hypoxia	0.216375	20.35	0	30

Table S4. Continued.

17	50	63.8	57.7	Severe hypoxia	0.22425	21.0625	0	21
17	70	77.5	59.6	Severe hypoxia	0.140125	20.9625	0	376
18	25	25.3	28.7	Severe hypoxia	0.119	20.375	42.40902	35
18	25	26.1	28.3	Severe hypoxia	0.204625	20.4	60.58432	28
18	25	26.4	26.9	Severe hypoxia	0.15275	20.4375	14.13634	105
18	25	35.3	26.5	Severe hypoxia	0.1955	20.8375	25.44541	50
18	35	44.6	34	Severe hypoxia	0.26	21.625	0	31
18	35	40.2	32.1	Severe hypoxia	0.296125	20.7125	0	11
18	35	36.3	41	Severe hypoxia	0.182875	20.7125	0	14
19	25	32.2	26.5	Severe hypoxia	0.14375	22.0875	49.88672	204
19	25	25.3	25.5	Severe hypoxia	0.127125	23.525	59.272	100
19	25	25.7	25.6	Severe hypoxia	0.38275	21.6	50.83362	165
19	35	38.6	31.2	Severe hypoxia	0.282125	21.0375	1.591134	492
19	25	29.7	25.3	Severe hypoxia	0.40025	20.6375	61.60345	354
19	35	35.4	27.2	Severe hypoxia	0.2835	21.3125	0	91
20	25	25.6	25.5	Severe hypoxia	0.288	21.6125	25.73262	94
20	25	27.2	26	Severe hypoxia	0.389375	20.875	0	22
20	35	34.9	30.6	Severe hypoxia	0.05425	23.05	1.103498	137
20	35	33.7	39.8	Severe hypoxia	0.388625	20.9375	0	47
21	0	0	0	Severe hypoxia	0.301625	21.625	84.55882	136
21	0	0	0	Severe hypoxia	0.147	20.4875	46.37681	69
21	0	0	0	Severe hypoxia	0.129125	19.9375	81.893	243
21	50	58.4	42.5	Severe hypoxia	0.333125	19.9375	2.291693	244
21	35	44.1	41.7	Severe hypoxia	0.0975	22.1625	3.883147	288
21	50	62.4	53.8	Severe hypoxia	0.204125	19.65	0	250
21	50	54.6	63.9	Severe hypoxia	0.204375	20.5375	0	266
21	50	67	54.1	Severe hypoxia	0	21.4375	0	413
21	50	63	50.3	Severe hypoxia	0.03025	21.025	0	252
22	35	44.8	32.5	Severe hypoxia	0.1335	20.4125	0	300
25	0	0	0	Severe hypoxia	0.239	20.05	56.71642	134
25	0	0	0	Severe hypoxia	0.31175	19.7625	66.5272	717
26	0	0	0	Severe hypoxia	0.19	20.325	68.05556	72
27	0	0	0	Severe hypoxia	0.69175	20.3625	49.82456	285
27	0	0	0	Severe hypoxia	0.11825	26.3625	100	246
28	0	0	0	Severe hypoxia	0.419375	21.0125	81.39535	43
30	0	0	0	Severe hypoxia	0.355375	21.975	48.88889	45
30	50	51.4	46.3	Severe hypoxia	0.31625	23.9625	0	282
30	50	47.4	53.6	Severe hypoxia	0.464375	21.45	0	51
30	50	53.1	47	Severe hypoxia	0.446875	25.325	0	75
31	70	75.6	69.1	Severe hypoxia	0.545625	20.2875	0	29
31	0	0	0	Severe hypoxia	0.17825	21.5375	88.83495	206
31	35	37.8	34.4	Severe hypoxia	0.3845	26.45	0	290
31	50	54.5	54	Severe hypoxia	0.328	21.15	0	542
31	70	83.7	77.6	Severe hypoxia	0.1185	25.8	0	694

Table S5. Experimental data for *Bactrocera dorsalis*

Block	Nominal dose	Absorbed dose: bottom	Absorbed dose: middle	Absorbed dose: top	Atmospheric conditions	O_2 (%)	CO_2 (%)	Adult emergence (corrected)	No. insects treated
1	0	0	0	0	Normoxia	21	0	84.61538	26
1	0	0	0	0	Normoxia	21	0	96.59091	88
1	0	0	0	0	Normoxia	21	0	94.2623	122
1	0	0	0	0	Normoxia	21	0	89.72973	185
1	0	0	0	0	Normoxia	21	0	72.6776	183
1	0	0	0	0	Normoxia	21	0	100	234
1	0	0	0	0	Normoxia	21	0	25.35211	142
1	0	0	0	0	Normoxia	21	0	79.60526	152
1	40	53.9	56.8	55.4	Normoxia	21	0	0	259
1	40	53.9	56.8	55.4	Normoxia	21	0	0	134
1	40	53.9	56.8	55.4	Normoxia	21	0	0	138
1	40	53.9	56.8	55.4	Normoxia	21	0	6.913837	126
1	40	59.7	57.1	55.9	Normoxia	21	0	2.074151	240
1	40	59.7	57.1	55.9	Normoxia	21	0	0	4
1	40	59.7	57.1	55.9	Normoxia	21	0	0	41
1	40	59.7	57.1	55.9	Normoxia	21	0	1.659321	75
2	0	0	0	0	Normoxia	21	0	98.4	125
2	0	0	0	0	Normoxia	21	0	65.65217	230
2	0	0	0	0	Normoxia	21	0	68.94737	190
2	0	0	0	0	Normoxia	21	0	70.3125	64
2	0	0	0	0	Normoxia	21	0	90.2439	41
2	0	0	0	0	Normoxia	21	0	83.46457	127
2	0	0	0	0	Normoxia	21	0	95.2381	63
2	0	0	0	0	Normoxia	21	0	73.2852	277
2	0	0	0	0	Normoxia	21	0	41.93548	93
2	40	44	54	45.3	Normoxia	21	0	1.540153	85
2	40	44	54	45.3	Normoxia	21	0	2.247434	233
2	40	44	54	45.3	Normoxia	21	0	2.257121	58
2	40	44	54	45.3	Normoxia	21	0	1.973563	199
2	40	44	54	45.3	Normoxia	21	0	0	356
3	0	0	0	0	Normoxia	21	0	85.04673	107
3	0	0	0	0	Normoxia	21	0	84.61538	26
3	0	0	0	0	Normoxia	21	0	86.95652	138
3	0	0	0	0	Normoxia	21	0	89.47368	76
3	0	0	0	0	Normoxia	21	0	100	19
3	0	0	0	0	Normoxia	21	0	100	8
3	0	0	0	0	Normoxia	21	0	85.71429	42
3	0	0	0	0	Normoxia	21	0	85.47009	117
3	0	0	0	0	Normoxia	21	0	95.09804	102
3	40	43.4	62.6	46.5	Normoxia	21	0	4.261012	104
3	40	43.4	62.6	46.5	Normoxia	21	0	6.154795	18
3	40	43.4	62.6	46.5	Normoxia	21	0	41.54487	8
3	40	43.4	62.6	46.5	Normoxia	21	0	4.344561	51
3	40	43.4	62.6	46.5	Normoxia	21	0	6.330646	35
3	40	45.7	61.8	49.8	Normoxia	21	0	2.215726	50
3	40	45.7	61.8	49.8	Normoxia	21	0	7.729278	43
3	40	45.7	61.8	49.8	Normoxia	21	0	1.007148	220
4	150	151.3	156.8	137.6	Normoxia	21	0	0	24
4	150	151.3	156.8	137.6	Normoxia	21	0	0	5
4	150	149.9	160.5	149.3	Normoxia	21	0	0	27
4	150	149.9	160.5	149.3	Normoxia	21	0	0	75
4	150	158.6	154.7	143.9	Normoxia	21	0	0	90
4	150	150.6	158.8	147.2	Normoxia	21	0	0	129
4	150	150.6	158.8	147.2	Normoxia	21	0	0	198
4	0	0	0	0	Normoxia	21	0	100	8
4	0	0	0	0	Normoxia	21	0	93.61702	94

Table S5. Continued.

4	0	0	0	0	Normoxia	21	0	100	1
4	0	0	0	0	Normoxia	21	0	67.76316	152
4	0	0	0	0	Normoxia	21	0	93.98496	266
4	0	0	0	0	Normoxia	21	0	95.34884	172
4	0	0	0	0	Normoxia	21	0	95.52239	67
4	0	0	0	0	Normoxia	21	0	100	20
5	150	149	155.9	142.5	Normoxia	21	0	0	237
5	150	149	155.9	142.5	Normoxia	21	0	0	78
5	150	152.4	157.5	141.1	Normoxia	21	0	0	375
5	150	152.4	157.5	141.1	Normoxia	21	0	0	6
5	150	143.2	155.5	142.4	Normoxia	21	0	0	111
5	150	143.2	155.5	142.4	Normoxia	21	0	0	81
5	0	0	0	0	Normoxia	21	0	96.89922	129
5	0	0	0	0	Normoxia	21	0	74.63768	138
5	0	0	0	0	Normoxia	21	0	93.39623	106
5	0	0	0	0	Normoxia	21	0	84.18972	253
5	0	0	0	0	Normoxia	21	0	100	5
5	0	0	0	0	Normoxia	21	0	94.18605	86
5	0	0	0	0	Normoxia	21	0	100	96
6	150	146.7	153.2	127.7	Normoxia	21	0	0	445
6	150	146.7	153.2	127.7	Normoxia	21	0	0	700
6	0	0	0	0	Normoxia	21	0	66.66667	186
6	0	0	0	0	Normoxia	21	0	88.70968	62
6	0	0	0	0	Normoxia	21	0	69.40452	487
6	0	0	0	0	Normoxia	21	0	100	1
6	0	0	0	0	Normoxia	21	0	98.31579	475
6	0	0	0	0	Normoxia	21	0	88.88889	27
6	0	0	0	0	Normoxia	21	0	66.59091	440
6	0	0	0	0	Normoxia	21	0	79.76654	514
6	0	0	0	0	Normoxia	21	0	66.44407	599
6	150	147.6	151.2	123.6	Normoxia	21	0	0	537
6	150	148.7	154.9	129.9	Normoxia	21	0	0	86
6	150	148.7	154.9	129.9	Normoxia	21	0	0	672
6	150	143.9	155.8	131	Normoxia	21	0	0	9
7	40	45.5	47.4	47.3	Normoxia	21	0	9.090909	11
7	40	45.5	47.4	47.3	Normoxia	21	0	0.735294	136
7	40	45	47.8	46.7	Normoxia	21	0	0	8
7	40	45	47.8	46.7	Normoxia	21	0	0.793651	126
7	40	47.4	47.9	44.6	Normoxia	21	0	1.06383	94
7	40	47.4	47.9	44.6	Normoxia	21	0	0	106
7	0	0	0	0	Normoxia	21	0	100	2
7	0	0	0	0	Normoxia	21	0	78.94737	38
7	0	0	0	0	Normoxia	21	0	98.71795	78
7	0	0	0	0	Normoxia	21	0	100	3
7	0	0	0	0	Normoxia	21	0	100	3
7	0	0	0	0	Normoxia	21	0	100	14
7	0	0	0	0	Normoxia	21	0	91.57895	95
8	116	116.3	119.5	104.2	Normoxia	21	0	0	44
8	116	115	120.7	109.5	Normoxia	21	0	0	6
8	116	115	120.7	109.5	Normoxia	21	0	0	33
8	116	111.1	117.2	112.1	Normoxia	21	0	0	7
8	116	111.1	117.2	112.1	Normoxia	21	0	0	22
8	0	0	0	0	Normoxia	21	0	100	6
8	0	0	0	0	Normoxia	21	0	18.18182	11
8	0	0	0	0	Normoxia	21	0	90.90909	11
8	0	0	0	0	Normoxia	21	0	89.81481	108
8	0	0	0	0	Normoxia	21	0	100	9
8	0	0	0	0	Normoxia	21	0	95.57522	113
8	0	0	0	0	Normoxia	21	0	100	1
8	0	0	0	0	Normoxia	21	0	75	12
8	0	0	0	0	Normoxia	21	0	75	12

Table S5. Continued.

9	0	0	0	0	Normoxia	21	0	89.77273	176
9	0	0	0	0	Normoxia	21	0	80	50
9	0	0	0	0	Normoxia	21	0	91.35802	162
9	0	0	0	0	Normoxia	21	0	75.35014	357
9	0	0	0	0	Normoxia	21	0	92.91667	240
9	0	0	0	0	Normoxia	21	0	83.3795	361
9	0	0	0	0	Normoxia	21	0	97.46835	79
9	0	0	0	0	Normoxia	21	0	81.0219	137
9	0	0	0	0	Normoxia	21	0	98.99497	199
9	150	142.3	155.7	133.4	Normoxia	21	0	0	72
9	150	142.3	155.7	133.4	Normoxia	21	0	0	175
9	150	145.1	150.1	133.2	Normoxia	21	0	0	218
9	150	145.1	150.1	133.2	Normoxia	21	0	0	176
9	150	150.3	156.2	126.1	Normoxia	21	0	0	68
9	150	150.3	156.2	126.1	Normoxia	21	0	0	142
10	0	0	0	0	Normoxia	21	0	93.38235	272
10	0	0	0	0	Normoxia	21	0	92.891	211
10	0	0	0	0	Normoxia	21	0	94.61538	130
10	0	0	0	0	Normoxia	21	0	91.39073	302
10	0	0	0	0	Normoxia	21	0	84.18079	531
10	0	0	0	0	Normoxia	21	0	92.63158	95
10	0	0	0	0	Normoxia	21	0	90.30471	361
10	0	0	0	0	Normoxia	21	0	97.65625	256
10	150	161	154.9	156.6	Normoxia	21	0	0	76
10	150	161	154.9	156.6	Normoxia	21	0	0	346
10	150	142.8	161.5	125.5	Normoxia	21	0	0	168
10	150	142.8	161.5	125.5	Normoxia	21	0	0	267
10	150	152.5	154	125.5	Normoxia	21	0	0	97
10	150	152.5	154	125.5	Normoxia	21	0	0	212
10	150	147.5	154.6	137.9	Normoxia	21	0	0	115
10	150	147.5	154.6	137.9	Normoxia	21	0	0	158
11	116	111.9	123.1	104.8	Normoxia	21	0	0	101
11	116	111.9	123.1	104.8	Normoxia	21	0	0	191
11	116	115.6	122.7	104	Normoxia	21	0	0	86
11	116	115.6	122.7	104	Normoxia	21	0	0	26
11	116	112.5	123.3	104.3	Normoxia	21	0	0	26
11	116	112.5	123.3	104.3	Normoxia	21	0	0	107
11	0	0	0	0	Normoxia	21	0	83.92157	255
11	0	0	0	0	Normoxia	21	0	82.17391	230
11	0	0	0	0	Normoxia	21	0	84.61538	247
11	0	0	0	0	Normoxia	21	0	92.77778	180
11	0	0	0	0	Normoxia	21	0	98.42932	191
11	0	0	0	0	Normoxia	21	0	81.91489	282
12	116	120.3	122.1	108.3	Normoxia	21	0	0	80
12	116	120.3	122.1	108.3	Normoxia	21	0	0	55
12	0	0	0	0	Normoxia	21	0	84.36019	211
12	0	0	0	0	Normoxia	21	0	79.86577	149
13	116	121.1	119.5	115.2	Normoxia	21	0	0	93
13	116	121.1	119.5	115.2	Normoxia	21	0	0	302
13	116	113.8	123.7	110.2	Normoxia	21	0	0	136
13	116	113.8	123.7	110.2	Normoxia	21	0	0	111
13	116	112.3	118.2	106.8	Normoxia	21	0	0	427
13	116	112.3	118.2	106.8	Normoxia	21	0	0	147
13	0	0	0	0	Normoxia	21	0	90.32258	31
13	0	0	0	0	Normoxia	21	0	96.36364	55
13	0	0	0	0	Normoxia	21	0	83.18584	339
13	0	0	0	0	Normoxia	21	0	92	50
13	0	0	0	0	Normoxia	21	0	79.45205	73
14	116	131.5	132.2	122.1	Normoxia	21	0	0	2
14	0	0	0	0	Normoxia	21	0	33.33333	3
15	116	131.4	136.2	121.4	Normoxia	21	0	0	17
15	116	131.4	136.2	121.4	Normoxia	21	0	0	1

Table S5. Continued.

15	116	126.2	136.4	131	Normoxia	21	0	0	2
15	116	126.2	136.4	131	Normoxia	21	0	0	1
15	116	121.3	129	125.2	Normoxia	21	0	0	52
15	116	121.3	129	125.2	Normoxia	21	0	0	39
15	0	0	0	0	Normoxia	21	0	88.0597	67
15	0	0	0	0	Normoxia	21	0	100	48
15	0	0	0	0	Normoxia	21	0	97.10145	69
15	0	0	0	0	Normoxia	21	0	76.19048	21
15	0	0	0	0	Normoxia	21	0	88.60104	193
16	116	121.4	124.4	105.7	Normoxia	21	0	0	75
16	116	121.4	124.4	105.7	Normoxia	21	0	0	184
16	0	0	0	0	Normoxia	21	0	95.625	160
16	0	0	0	0	Normoxia	21	0	100	1
16	0	0	0	0	Normoxia	21	0	96.92308	65
16	0	0	0	0	Normoxia	21	0	94.02985	201
16	116	118.9	124.9	103.5	Normoxia	21	0	0	227
16	116	118.9	124.9	103.5	Normoxia	21	0	0	82
16	116	121.2	124.5	107.4	Normoxia	21	0	0	141
16	116	121.2	124.5	107.4	Normoxia	21	0	0	34
17	0	0	0	0	Normoxia	21	0	100	7
17	0	0	0	0	Normoxia	21	0	100	3
17	0	0	0	0	Normoxia	21	0	90.90909	22
17	0	0	0	0	Normoxia	21	0	94.44444	18
17	116	110.6	119.9	116.6	Normoxia	21	0	0	12
17	116	110.6	119.9	116.6	Normoxia	21	0	0	116
17	116	109.7	118.3	104.5	Normoxia	21	0	0	61
17	116	109.7	118.3	104.5	Normoxia	21	0	0	5
17	116	109.7	118.3	104.3	Normoxia	21	0	0	49
17	116	109.7	118.3	104.3	Normoxia	21	0	0	37
17	116	110.6	119.9	116.6	Normoxia	21	0	0	11
17	116	110.6	119.9	116.6	Normoxia	21	0	0	53
18	0	0	0	0	Normoxia	21	0	95.73171	164
18	0	0	0	0	Normoxia	21	0	90.69767	43
18	116	118.6	121	110	Normoxia	21	0	0	25
18	116	118.6	121	110	Normoxia	21	0	0	59
18	116	118.6	121	110	Normoxia	21	0	0	24
18	116	118.6	121	110	Normoxia	21	0	0	17
18	116	114	122	113.9	Normoxia	21	0	0	1
18	116	114	122	113.9	Normoxia	21	0	0	58
18	116	117.8	116.1	110.2	Normoxia	21	0	0	20
19	0	0	0	0	Normoxia	21	0	50	2
19	0	0	0	0	Normoxia	21	0	50	8
19	0	0	0	0	Normoxia	21	0	100	1
19	0	0	0	0	Normoxia	21	0	4.040404	198
19	0	0	0	0	Normoxia	21	0	0	12
19	116	110.7	126	117	Normoxia	21	0	0	24
19	116	110.7	126	117	Normoxia	21	0	0	1
19	116	119	115.6	98.6	Normoxia	21	0	0	18
19	116	119	115.6	98.6	Normoxia	21	0	0	1
19	116	119	115.6	98.6	Normoxia	21	0	0	12
19	116	117.9	116.6	100.6	Normoxia	21	0	0	4
19	116	117.9	116.6	100.6	Normoxia	21	0	0	1
20	40	44.5	48.5	44.3	Normoxia	21	0	0	3
20	40	44.5	48.5	44.3	Normoxia	21	0	11.81028	224
20	0	0	0	0	Normoxia	21	0	74.78632	234
20	0	0	0	0	Normoxia	21	0	76.33588	131
20	116	104.8	115	103.6	Normoxia	21	0	0	43
20	116	104.8	115	103.6	Normoxia	21	0	0	30
21	30	41.4	37.6	34.5	Normoxia	21	0	19.06542	107
21	80	63.4	90.4	75.2	Normoxia	21	0	0	6

Table S5. Continued.

21	80	63.4	90.4	75.2	Normoxia	21	0	0	147
21	40	47	42.5	38.9	Normoxia	21	0	17.9019	114
21	0	0	0	0	Normoxia	21	0	66.66667	18
21	0	0	0	0	Normoxia	21	0	100	26
21	116	105.6	121.1	105.1	Normoxia	21	0	0	24
21	116	105.6	121.1	105.1	Normoxia	21	0	0	42
22	30	32	40.1	43.1	Normoxia	21	0	4.816144	131
22	30	32	40.1	43.1	Normoxia	21	0	0	34
22	40	51.5	44.5	40	Normoxia	21	0	0	11
22	0	0	0	0	Normoxia	21	0	95.08197	61
22	116	105.4	116	103.4	Normoxia	21	0	0	53
22	116	105.4	116	103.4	Normoxia	21	0	0	68
23	80	67.1	78.2	80.7	Normoxia	21	0	0	213
23	80	67.1	78.2	80.7	Normoxia	21	0	0	15
23	40	46.6	42	44.7	Normoxia	21	0	0	420
23	0	0	0	0	Normoxia	21	0	33.33333	12
23	0	0	0	0	Normoxia	21	0	92.17391	115
23	116	110.2	114.2	104.4	Normoxia	21	0	0	10
23	116	110.2	114.2	104.4	Normoxia	21	0	0	2
24	0	0	0	0	Normoxia	21	0	50	2
24	116	100.7	121.1	95.7	Normoxia	21	0	0	226
24	116	100.7	121.1	95.7	Normoxia	21	0	0	62
24	116	100.7	121.1	95.7	Normoxia	21	0	0	201
24	116	100.7	121.1	95.7	Normoxia	21	0	0	168
25	30	37.5	37.1	38.5	Normoxia	21	0	0	31
25	30	37.5	37.1	38.5	Normoxia	21	0	10.2661	128
25	80	64.6	71.2	68.8	Normoxia	21	0	0	229
25	80	64.6	71.2	68.8	Normoxia	21	0	0	39
25	40	45	44.6	43.9	Normoxia	21	0	6.041657	435
25	0	0	0	0	Normoxia	21	0	92	25
25	0	0	0	0	Normoxia	21	0	60	40
25	0	0	0	0	Normoxia	21	0	76.34409	93
25	116	124.9	122	97.4	Normoxia	21	0	0	59
25	116	124.9	122	97.4	Normoxia	21	0	0	245
25	116	121.5	113.1	93.8	Normoxia	21	0	0	86
25	116	121.5	113.1	93.8	Normoxia	21	0	0	23
26	30	37.3	42.8	41.1	Normoxia	21	0	6.517957	249
26	30	37.3	42.8	41.1	Normoxia	21	0	0	1
26	80	79.1	85.1	75	Normoxia	21	0	0	6
26	80	79.1	85.1	75	Normoxia	21	0	0	114
26	40	44.6	44.6	43.4	Normoxia	21	0	0	17
26	0	0	0	0	Normoxia	21	0	72.91667	192
26	0	0	0	0	Normoxia	21	0	87.36264	182
26	116	113.9	125.1	120.7	Normoxia	21	0	0	73
26	116	113.9	125.1	120.7	Normoxia	21	0	0	96
26	116	120.7	125.4	111.2	Normoxia	21	0	0	8
26	116	120.7	125.4	111.2	Normoxia	21	0	0	25
27	80	69.2	82.7	72.7	Normoxia	21	0	0	48
27	80	69.2	82.7	72.7	Normoxia	21	0	0	66
27	40	39.5	50.6	44.9	Normoxia	21	0	2.057275	217
27	40	39.5	50.6	44.9	Normoxia	21	0	0	142
27	0	0	0	0	Normoxia	21	0	100	20
27	0	0	0	0	Normoxia	21	0	79.13669	278
27	116	107.2	122.9	111.9	Normoxia	21	0	0	35
27	116	107.2	122.9	111.9	Normoxia	21	0	0	222
27	116	110	131.5	123.6	Normoxia	21	0	0	5
27	116	110	131.5	123.6	Normoxia	21	0	0	141
28	40	38.8	45.1	43.5	Normoxia	21	0	0	192
28	40	38.8	45.1	43.5	Normoxia	21	0	4.293227	55
28	40	43.7	42.9	41.2	Normoxia	21	0	9.977219	71
28	0	0	0	0	Normoxia	21	0	82.82443	262
28	0	0	0	0	Normoxia	21	0	86.52482	141
29	40	42.1	44.3	41.5	Normoxia	21	0	0	35

Table S5. Continued.

29	40	42.1	44.3	41.5	Normoxia	21	0	0	5
29	0	0	0	0	Normoxia	21	0	87.39496	119
29	0	0	0	0	Normoxia	21	0	75	4
29	116	106	108.2	119.7	Normoxia	21	0	0	70
29	116	106	108.2	119.7	Normoxia	21	0	0	30
29	116	120.6	112.1	97.4	Normoxia	21	0	0	28
29	116	104	101.1	124.9	Normoxia	21	0	0	63
29	116	104	101.1	124.9	Normoxia	21	0	0	194
30	0	0	0	0	Normoxia	21	0	96.42857	28
30	0	0	0	0	Normoxia	21	0	90.625	64
30	116	108.3	119.4	99.2	Normoxia	21	0	0	24
30	116	108.3	119.4	99.2	Normoxia	21	0	0	197
31	30	40.9	42.6	47.9	Normoxia	21	0	0	61
31	30	40.9	42.6	47.9	Normoxia	21	0	0	8
31	80	90.3	88.5	80.2	Normoxia	21	0	0	117
31	80	90.3	88.5	80.2	Normoxia	21	0	0	12
31	0	0	0	0	Normoxia	21	0	91.26984	126
31	0	0	0	0	Normoxia	21	0	89	100
32	30	27.8	30.5	30.8	Normoxia	21	0	13.15547	41
32	30	27.8	30.5	30.8	Normoxia	21	0	0	62
32	0	0	0	0	Normoxia	21	0	92.10526	38
32	0	0	0	0	Normoxia	21	0	93.37017	181
32	116	114.8	135.2	120.2	Normoxia	21	0	0	24
32	116	114.8	135.2	120.2	Normoxia	21	0	0	107
32	116	112.4	132.2	127.5	Normoxia	21	0	0	14
32	116	112.4	132.2	127.5	Normoxia	21	0	0	115
33	0	0	0	0	Normoxia	21	0	90	10
33	0	0	0	0	Normoxia	21	0	88.52459	61
33	116	124.9	127	121.5	Normoxia	21	0	0	2
33	116	124.9	127	121.5	Normoxia	21	0	0	124
34	30	31.4	36.4	36.3	Normoxia	21	0	0	133
34	30	31.4	36.4	36.3	Normoxia	21	0	18.76349	57
34	80	78.3	84.1	91.3	Normoxia	21	0	0	205
34	80	78.3	84.1	91.3	Normoxia	21	0	0.279979	382
34	0	0	0	0	Normoxia	21	0	86.91589	107
34	0	0	0	0	Normoxia	21	0	100	2
35	30	31	33.5	30.3	Normoxia	21	0	4.056631	27
35	30	31	33.5	30.3	Normoxia	21	0	5.36907	102
35	80	68.6	81	74.7	Normoxia	21	0	0	379
35	80	68.6	81	74.7	Normoxia	21	0	0	311
35	0	0	0	0	Normoxia	21	0	91.30435	92
36	0	0	0	0	Normoxia	21	0	71.05263	114
36	0	0	0	0	Normoxia	21	0	83.06452	124
20	0	0	0	0	Hypoxia	5.03	14.275	96.875	32
20	0	0	0	0	Hypoxia	5.03	14.275	62.33766	77
20	40	48.8	43.5	43.7	Hypoxia	4.84625	16.0625	5.591967	71
20	40	48.8	43.5	43.7	Hypoxia	4.84625	16.0625	7.887967	218
20	116	135.3	95.4	115.9	Hypoxia	5.14	14.65	0	28
20	116	135.3	95.4	115.9	Hypoxia	5.14	14.65	0	4
20	40	46.2	46.3	49	Hypoxia	5.69	13.8125	12.80082	62
20	40	46.2	46.3	49	Hypoxia	5.69	13.8125	0	41
20	116	122.6	110.5	131.4	Hypoxia	5.18875	14.65	0	29
20	116	122.6	110.5	131.4	Hypoxia	5.18875	14.65	0	5
20	116	119.3	122	91.6	Hypoxia	5.29125	15.4125	0	195
20	116	119.3	122	91.6	Hypoxia	5.29125	15.4125	0	78
21	30	35.9	40.6	39.8	Hypoxia	4.9475	16.4625	29.32991	176
21	30	35.9	40.6	39.8	Hypoxia	4.9475	16.4625	45.87201	123
21	80	78.9	88.4	66.8	Hypoxia	5.48	14.9125	0	28
21	0	0	0	0	Hypoxia	4.95	16.9875	51.51515	165

Table S5. Continued.

21	0	0	0	0	Hypoxia	4.95	16.9875	85.16129	155
21	0	0	0	0	Hypoxia	5.66125	15.3	38.88889	18
21	0	0	0	0	Hypoxia	5.66125	15.3	54.31034	232
21	40	46	43.1	43	Hypoxia	5.15875	14.825	84.73978	17
21	40	46	43.1	43	Hypoxia	5.15875	14.825	0	7
21	40	48.6	45.8	41	Hypoxia	4.84	15.4	0	49
21	116	116.9	119.1	118.5	Hypoxia	7.14125	12.3625	0	14
21	116	116.9	119.1	118.5	Hypoxia	7.14125	12.3625	0	37
21	116	117.9	121	133.9	Hypoxia	4.9325	15.5625	0	108
21	116	116.2	115.8	85.2	Hypoxia	4.54	13.5	0	7
21	116	116.2	115.8	85.2	Hypoxia	4.54	13.5	0	92
21	116	98.9	117.1	112.7	Hypoxia	5.0875	15.7	0	4
21	116	98.9	117.1	112.7	Hypoxia	5.0875	15.7	0	133
22	30	38.2	37.6	37.5	Hypoxia	5.9525	14.8125	51.68511	59
22	80	67.9	86.6	71.5	Hypoxia	4.86375	16.6375	0	1
22	80	67.9	86.6	71.5	Hypoxia	4.86375	16.6375	0	7
22	0	0	0	0	Hypoxia	5.31	15.025	96.2963	27
22	0	0	0	0	Hypoxia	5.31	15.025	100	2
22	0	0	0	0	Hypoxia	5.31	15.025	4.597701	87
22	40	47.6	42.4	42.8	Hypoxia	5.1675	15.6375	0	29
22	116	131.9	116.4	137.2	Hypoxia	4.86375	16.6375	0	8
22	116	131.9	116.4	137.2	Hypoxia	4.86375	16.6375	0	4
22	116	104.8	121.3	104.1	Hypoxia	6.57875	13.7125	0	417
22	116	104.8	121.3	104.1	Hypoxia	6.57875	13.7125	0	4
23	80	78.1	82.6	66.7	Hypoxia	5.716667	14.65	0	402
23	80	78.1	82.6	66.7	Hypoxia	5.716667	14.65	0	124
23	80	78.5	80.5	77.1	Hypoxia	4.423333	14.85	0	88
23	80	78.5	80.5	77.1	Hypoxia	4.423333	14.85	0	55
23	0	0	0	0	Hypoxia	4.401667	15.21667	60	25
23	0	0	0	0	Hypoxia	4.401667	15.21667	88.88889	99
23	40	46	48.8	45.3	Hypoxia	5	14.55	31.84713	180
23	40	46	48.8	45.3	Hypoxia	5	14.55	7.237985	44
23	116	123.6	131.6	149.6	Hypoxia	5.376667	15.23333	0	129
23	116	123.6	131.6	149.6	Hypoxia	5.376667	15.23333	0	176
23	116	111.4	126.9	108.6	Hypoxia	5.15	10.78	0	118
23	116	111.4	126.9	108.6	Hypoxia	5.15	10.78	0	80
23	116	130.7	100.4	126.9	Hypoxia	5.166667	14.73333	0	12
23	116	130.7	100.4	126.9	Hypoxia	5.166667	14.73333	0	3
24	0	0	0	0	Hypoxia	4.6025	16.1625	85.71429	35
24	116	101	138.7	111.4	Hypoxia	4.69125	14.4125	0	11
24	116	101	138.7	111.4	Hypoxia	4.69125	14.4125	0	35
24	116	116.7	125.2	109.1	Hypoxia	4.405	16.325	0	21
24	116	116.7	125.2	109.1	Hypoxia	4.405	16.325	0	71
25	30	38	43.9	34.5	Hypoxia	5	12.75714	2.456188	107
25	30	38	43.9	34.5	Hypoxia	5	12.75714	0	1
25	80	62.9	76.8	67.8	Hypoxia	4.244286	13.9	0	131
25	80	62.9	76.8	67.8	Hypoxia	4.244286	13.9	0	86
25	0	0	0	0	Hypoxia	5.177143	14.01429	92.85714	238
25	0	0	0	0	Hypoxia	5.177143	14.01429	61.45251	179
25	40	48.9	39.9	49.2	Hypoxia	4.775714	13.62857	6.410051	41
25	40	48.9	39.9	49.2	Hypoxia	4.775714	13.62857	7.337393	197
25	0	0	0	0	Hypoxia	5.942857	12.05714	90.625	32
25	0	0	0	0	Hypoxia	5.942857	12.05714	93.93939	33
25	40	43.8	42.9	41.5	Hypoxia	4.804286	14.1	0.264398	497
25	40	43.8	42.9	41.5	Hypoxia	4.804286	14.1	9.198423	100
25	116	127	124.7	90	Hypoxia	4.937143	13.17143	0	26
25	116	106.9	138.6	133.8	Hypoxia	5.73	12.95714	0	1
25	116	106.9	138.6	133.8	Hypoxia	5.73	12.95714	0	19
25	116	112.7	108.5	113.6	Hypoxia	4.708571	13.92857	0	46
25	116	112.7	108.5	113.6	Hypoxia	4.708571	13.92857	0	69
26	150	155	158.2	197.2	Hypoxia	5.57625	13.325	0	70
26	150	155	158.2	197.2	Hypoxia	5.57625	13.325	0	61
26	30	37.9	38.9	38.2	Hypoxia	4.95625	14.8125	0	3
26	80	89.3	90.4	86.8	Hypoxia	4.73375	15.6875	0	25

Table S5. Continued.

26	80	89.3	90.4	86.8	Hypoxia	4.73375	15.6875	0	110
26	0	0	0	0	Hypoxia	6.5625	13.325	92.12598	127
26	0	0	0	0	Hypoxia	6.5625	13.325	77.08333	144
26	40	44.3	52.4	35.7	Hypoxia	5.81375	14.4125	1.006806	248
26	40	44.3	52.4	35.7	Hypoxia	5.81375	14.4125	0	331
26	116	125.1	134.7	130.3	Hypoxia	5.73375	15.0625	0	74
26	116	125.1	134.7	130.3	Hypoxia	5.73375	15.0625	0	191
27	150	134.2	160.7	142.3	Hypoxia	4.71875	15.225	0	15
27	150	161.2	149.3	146.7	Hypoxia	4.38375	15.9875	0	135
27	150	161.2	149.3	146.7	Hypoxia	4.38375	15.9875	0	152
27	80	83.6	87.7	66.2	Hypoxia	4.43	15.3875	0	3
27	80	83.6	87.7	66.2	Hypoxia	4.43	15.3875	0	123
27	0	0	0	0	Hypoxia	4.5175	16.2625	66.26506	166
27	0	0	0	0	Hypoxia	4.5175	16.2625	80.55556	36
27	40	52.6	42.8	42.4	Hypoxia	4.89125	15.1	0.424362	263
27	40	52.6	42.8	42.4	Hypoxia	4.89125	15.1	41.85268	8
27	0	0	0	0	Hypoxia	4.43125	17.0375	100	61
27	0	0	0	0	Hypoxia	4.43125	17.0375	70	70
27	116	113	120.2	123.5	Hypoxia	5.5	14.45	0	27
27	116	113	120.2	123.5	Hypoxia	5.5	14.45	0	39
28	150	146.3	149.8	158.8	Hypoxia	5.03125	15.625	0	49
28	150	146.3	149.8	158.8	Hypoxia	5.03125	15.625	0	57
28	150	128.6	167.6	152.3	Hypoxia	5.595	14.575	0	42
28	150	128.6	167.6	152.3	Hypoxia	5.595	14.575	0	190
28	150	142	143.3	165.6	Hypoxia	4.6125	15.9625	0	60
28	150	142	143.3	165.6	Hypoxia	4.6125	15.9625	0	162
28	40	42.3	44.2	40.9	Hypoxia	5.825	14.825	14.75797	40
28	0	0	0	0	Hypoxia	6.60125	13.1375	75	12
28	0	0	0	0	Hypoxia	6.60125	13.1375	100	1
28	40	46.4	51.3	47.7	Hypoxia	5.41875	15	29.51594	8
29	150	127	167.9	152.7	Hypoxia	7.635	11.8375	0	20
29	150	138.1	158.2	145.4	Hypoxia	6.84375	13.125	0	85
29	150	138.1	158.2	145.4	Hypoxia	6.84375	13.125	0	112
29	0	0	0	0	Hypoxia	5.05875	12.4625	95.65217	46
29	0	0	0	0	Hypoxia	5.05875	12.4625	92	50
29	40	42.3	44.5	44.2	Hypoxia	5.6075	14.425	51.06332	41
29	40	42.3	44.5	44.2	Hypoxia	5.6075	14.425	16.79355	22
29	0	0	0	0	Hypoxia	4.82625	15.925	96.22642	53
29	0	0	0	0	Hypoxia	4.82625	15.925	98.07692	52
29	116	106	128.1	124.1	Hypoxia	5.43	15.4	0	48
29	116	106	128.1	124.1	Hypoxia	5.43	15.4	0	40
29	116	125.7	113.7	123.7	Hypoxia	4.8375	14.775	0	1
29	116	125.7	113.7	123.7	Hypoxia	4.8375	14.775	0	19
29	116	109.8	127.5	109.2	Hypoxia	6.355	13.475	0	13
29	116	109.8	127.5	109.2	Hypoxia	6.355	13.475	0	14
30	150	140.3	147.6	142.6	Hypoxia	6.275	13.7	0	22
30	150	140.3	147.6	142.6	Hypoxia	6.275	13.7	0	180
30	0	0	0	0	Hypoxia	5.6525	13.5625	89.55224	134
30	0	0	0	0	Hypoxia	5.6525	13.5625	66.66667	3
30	0	0	0	0	Hypoxia	4.8725	15.6625	92.59259	135
30	0	0	0	0	Hypoxia	4.8725	15.6625	92.77778	180
30	0	0	0	0	Hypoxia	5.56875	13.9125	100	18
30	0	0	0	0	Hypoxia	5.56875	13.9125	74.31193	109
30	116	109.1	120.1	119	Hypoxia	5.56375	13.1875	0	18
30	116	109.1	120.1	119	Hypoxia	5.56375	13.1875	0	23
31	30	39.8	42.7	39.9	Hypoxia	5.31875	13.35	0	3
31	30	39.8	42.7	39.9	Hypoxia	5.31875	13.35	2.65521	209
31	30	41.7	42.1	40.8	Hypoxia	7.925	11.6875	32.37144	24
31	30	41.7	42.1	40.8	Hypoxia	7.925	11.6875	2.774695	120
31	80	79.1	85.2	84	Hypoxia	5.175	14.4875	0	290
31	80	79.1	85.2	84	Hypoxia	5.175	14.4875	0	23

Table S5. Continued.

31	80	77.6	84.3	80.1	Hypoxia	5.97125	13.7875	0	3
31	80	77.6	84.3	80.1	Hypoxia	5.97125	13.7875	0	146
31	40	39.7	47.6	51.6	Hypoxia	5.93	12.4375	0	128
31	40	39.7	47.6	51.6	Hypoxia	5.93	12.4375	1.261225	88
31	0	0	0	0	Hypoxia	5.80125	13.15	90.27778	72
31	0	0	0	0	Hypoxia	5.80125	13.15	89.00524	191
31	116	106.2	129.1	124.2	Hypoxia	5.51375	15.25	0	45
31	116	106.2	129.1	124.2	Hypoxia	5.51375	15.25	0	34
32	30	34.9	32.1	36.8	Hypoxia	5.24	14.975	18.49283	175
32	30	34.9	32.1	36.8	Hypoxia	5.24	14.975	19.83906	87
32	80	61.9	79.3	72.4	Hypoxia	5.36875	14.7	0	45
32	80	61.9	79.3	72.4	Hypoxia	5.36875	14.7	0	229
32	150	153.7	194	147.4	Hypoxia	4.66	16.1875	0	32
32	150	153.7	194	147.4	Hypoxia	4.66	16.1875	0	42
32	150	164.5	186.4	162.4	Hypoxia	4.755	15.35	0	98
32	150	164.5	186.4	162.4	Hypoxia	4.755	15.35	0	6
32	40	39.1	47.6	47.3	Hypoxia	5.52875	14.0375	0	38
32	40	39.1	47.6	47.3	Hypoxia	5.52875	14.0375	19.26337	28
32	116	110.7	128.2	121.2	Hypoxia	4.92625	15.15	0	60
32	116	110.7	128.2	121.2	Hypoxia	4.92625	15.15	0	81
32	116	127.9	129.6	123.8	Hypoxia	4.7175	14.35	0	66
32	116	127.9	129.6	123.8	Hypoxia	4.7175	14.35	0	137
33	30	32.8	37.1	36.3	Hypoxia	5.2775	15.325	2.036038	440
33	30	32.8	37.1	36.3	Hypoxia	5.2775	15.325	0.872588	385
33	150	174.5	156.4	197.6	Hypoxia	5.4225	14.95	0	92
33	150	174.5	156.4	197.6	Hypoxia	5.4225	14.95	0	53
33	40	41.8	46.9	54.2	Hypoxia	6.765	13.4875	1.696698	132
33	40	41.8	46.9	54.2	Hypoxia	6.765	13.4875	0	36
33	0	0	0	0	Hypoxia	5.9475	13.9375	84.61538	26
33	0	0	0	0	Hypoxia	5.9475	13.9375	92.68293	41
33	0	0	0	0	Hypoxia	5.36875	14.7	93.75	192
33	0	0	0	0	Hypoxia	5.36875	14.7	95.08197	61
33	116	133.2	139.7	151.9	Hypoxia	5.12625	14.975	0	214
33	116	133.2	139.7	151.9	Hypoxia	5.12625	14.975	0	101
33	116	122.4	149.2	137.5	Hypoxia	5.2675	14.075	0	159
33	116	122.4	149.2	137.5	Hypoxia	5.2675	14.075	0	204
34	30	34.2	38.9	38.6	Hypoxia	4.84875	15.8125	82.8877	40
34	30	34.2	38.9	38.6	Hypoxia	4.84875	15.8125	29.16869	11
34	30	34.2	38.9	38.6	Hypoxia	5.7175	14.925	0	19
34	30	34.2	38.9	38.6	Hypoxia	5.7175	14.925	2.673797	40
34	80	82.6	91.5	89.6	Hypoxia	5.93125	14.975	0	19
34	80	82.6	91.5	89.6	Hypoxia	5.93125	14.975	0	8
34	150	186.5	184.2	169.7	Hypoxia	5.48375	15.0125	0	2
34	150	186.5	184.2	169.7	Hypoxia	5.48375	15.0125	0	13
34	116	128.1	120.8	158.8	Hypoxia	5.48375	15.0125	0	2
34	116	128.1	120.8	158.8	Hypoxia	5.48375	15.0125	0	13
34	0	0	0	0	Hypoxia	4.441625	16.0125	79.06977	43
34	0	0	0	0	Hypoxia	4.441625	16.0125	94.2029	69
35	30	32.9	33.6	25.9	Hypoxia	4.93	17.05	0.365097	300
35	30	32.9	33.6	25.9	Hypoxia	4.93	17.05	23.56954	395
35	80	86.1	89.5	86.8	Hypoxia	5.235	15.6125	0	13
35	80	86.1	89.5	86.8	Hypoxia	5.235	15.6125	0	31
35	30	33.6	30.6	32	Hypoxia	5.76875	16.125	0.608495	360
35	30	33.6	30.6	32	Hypoxia	5.76875	16.125	9.957184	11
35	80	88.1	86.7	83.5	Hypoxia	5.54125	14.7875	0	17
35	80	88.1	86.7	83.5	Hypoxia	5.54125	14.7875	0	1
35	30	30.6	34.6	33.2	Hypoxia	5.57375	15.4625	24.18173	77
35	30	30.6	34.6	33.2	Hypoxia	5.57375	15.4625	0.470082	233
35	150	150.6	188.1	169.6	Hypoxia	5.725	14.5	0	4
35	150	150.6	188.1	168.6	Hypoxia	5.725	14.5	0	29
35	150	156.2	161	130.7	Hypoxia	4.87875	15.8625	0	10
35	150	156.2	161	130.7	Hypoxia	4.87875	15.8625	0	59

Table S5. Continued.

35	0	0	0	0	Hypoxia	4.6625	16.1375	100	3
35	0	0	0	0	Hypoxia	4.6625	16.1375	100	6
36	80	91.3	90	88.6	Hypoxia	5.005	15.2625	0	209
36	80	91.3	90	88.6	Hypoxia	5.005	15.2625	0	341
36	30	33	38.1	33.1	Hypoxia	4.8425	14.6625	2.981648	174
36	30	33	38.1	33.1	Hypoxia	4.8425	14.6625	25.94034	15
36	80	94.1	93.7	95.9	Hypoxia	4.95625	16.0375	0	262
36	80	94.1	93.7	95.9	Hypoxia	4.95625	16.0375	0	269
36	30	34.8	38.4	32.4	Hypoxia	4.5125	16.8125	13.69521	161
36	30	34.8	38.4	32.4	Hypoxia	4.5125	16.8125	0	5
36	80	75.3	85.9	86.3	Hypoxia	4.745	15.5625	0	291
36	80	75.3	85.9	86.3	Hypoxia	4.745	15.5625	0	319
36	30	31.4	37.9	38.2	Hypoxia	4.685	15.3	1.85819	349
36	30	31.4	37.9	38.2	Hypoxia	4.685	15.3	3.759469	138
36	40	49.4	51.6	45.7	Hypoxia	5.24875	14.875	25.166	201
36	40	49.4	51.6	45.7	Hypoxia	5.24875	14.875	0	273
36	40	49.4	51.6	45.7	Hypoxia	5.165	15.5625	0	240
36	40	49.4	51.6	45.7	Hypoxia	5.165	15.5625	0	167
36	40	46.6	52.7	47.5	Hypoxia	5.21	15.425	30.77667	354
36	40	46.6	52.7	47.5	Hypoxia	5.21	15.425	0.800628	324
36	0	0	0	0	Hypoxia	4.74125	15.525	70.8502	247
36	0	0	0	0	Hypoxia	4.74125	15.525	80.95238	42
36	0	0	0	0	Hypoxia	4.9825	15.0625	79.91071	224
36	116	128.8	133.3	118	Hypoxia	4.425	15.5	0	175
36	116	128.8	133.3	118	Hypoxia	4.425	15.5	0	357
36	116	110.5	113.2	118	Hypoxia	4.6725	15.825	0	312
36	116	110.5	113.2	118	Hypoxia	4.6725	15.825	0	155
36	116	114.7	119	116.4	Hypoxia	5.8475	14.2375	0	311
36	116	114.7	119	116.4	Hypoxia	5.8475	14.2375	0	50
1	0	0	0	0	Severe hypoxia	0.261143	22.94286	80.4878	82
1	0	0	0	0	Severe hypoxia	0.261143	22.94286	87.87879	165
1	0	0	0	0	Severe hypoxia	0.261143	22.94286	86.84211	38
1	0	0	0	0	Severe hypoxia	0.261143	22.94286	90.32258	62
1	40	58.7	60.2	53.9	Severe hypoxia	0.548143	21.12857	17.16539	29
1	40	58.7	60.2	53.9	Severe hypoxia	0.548143	21.12857	0	25
1	40	58.7	60.2	53.9	Severe hypoxia	0.548143	21.12857	14.17774	79
1	40	58.7	60.2	53.9	Severe hypoxia	0.548143	21.12857	0	63
1	40	58.7	60.2	53.9	Severe hypoxia	0.548143	21.12857	38.51995	42
1	40	58.7	60.2	53.9	Severe hypoxia	0.548143	21.12857	20.07243	93
1	40	58.7	60.2	53.9	Severe hypoxia	0.548143	21.12857	12.44491	130
1	0	0	0	0	Severe hypoxia	0.064067	21.54	95.48387	155
1	0	0	0	0	Severe hypoxia	0.064067	21.54	100	274
1	0	0	0	0	Severe hypoxia	0.064067	21.54	21.53846	65
1	0	0	0	0	Severe hypoxia	0.064067	21.54	90.37433	187
1	40	59.7	57.1	55.9	Severe hypoxia	0.311286	21.42857	17.77844	105
1	40	59.7	57.1	55.9	Severe hypoxia	0.311286	21.42857	29.49904	135
1	40	59.7	57.1	55.9	Severe hypoxia	0.311286	21.42857	9.304603	107
2	0	0	0	0	Severe hypoxia	0.431111	22.76667	75.16779	149
2	0	0	0	0	Severe hypoxia	0.431111	22.76667	21.47059	340
2	0	0	0	0	Severe hypoxia	0.431111	22.76667	71.02273	176
2	0	0	0	0	Severe hypoxia	0.431111	22.76667	86.34361	227
2	0	0	0	0	Severe hypoxia	0.431111	22.76667	47.08333	240
2	40	41.3	67.6	40.3	Severe hypoxia	0.220375	23.45	6.545652	300
2	40	41.3	67.6	40.3	Severe hypoxia	0.220375	23.45	0.928461	141
2	40	41.3	67.6	40.3	Severe hypoxia	0.220375	23.45	0	90
2	40	41.3	67.6	40.3	Severe hypoxia	0.220375	23.45	52.522	167
2	40	41.3	67.6	40.3	Severe hypoxia	0.220375	23.45	20.76807	353
3	0	0	0	0	Severe hypoxia	0.308125	22.225	83.33333	42
3	0	0	0	0	Severe hypoxia	0.308125	22.225	56.25	16
3	0	0	0	0	Severe hypoxia	0.308125	22.225	76.8595	242
3	0	0	0	0	Severe hypoxia	0.308125	22.225	66.13546	251

Table S5. Continued.

3	0	0	0	0	Severe hypoxia	0.308125	22.225	87.64045	356
3	0	0	0	0	Severe hypoxia	0.308125	22.225	96.21212	132
3	0	0	0	0	Severe hypoxia	0.308125	22.225	73.72263	137
3	0	0	0	0	Severe hypoxia	0.308125	22.225	88.37209	86
3	0	0	0	0	Severe hypoxia	0.308125	22.225	47.12042	382
3	40	44.4	59.6	48.1	Severe hypoxia	0.538375	21.5625	3.85823	201
3	40	44.4	59.6	48.1	Severe hypoxia	0.538375	21.5625	0	7
3	40	44.4	59.6	48.1	Severe hypoxia	0.538375	21.5625	6.569539	371
3	40	44.4	59.6	48.1	Severe hypoxia	0.538375	21.5625	10.55108	21
3	40	44.4	59.6	48.1	Severe hypoxia	0.538375	21.5625	3.258421	34
3	40	46.3	63.7	40.2	Severe hypoxia	0.186	21.6125	7.193916	77
3	40	46.3	63.7	40.2	Severe hypoxia	0.186	21.6125	1.877734	59
3	40	46.3	63.7	40.2	Severe hypoxia	0.186	21.6125	9.60148	150
3	40	46.3	63.7	40.2	Severe hypoxia	0.186	21.6125	21.36995	197
3	40	46.3	63.7	40.2	Severe hypoxia	0.186	21.6125	0	109
5	150	150.4	173	137.1	Severe hypoxia	0.474125	19.6875	0	69
5	150	150.4	173	137.1	Severe hypoxia	0.474125	19.6875	0	133
5	150	154.2	157	146.9	Severe hypoxia	0.502625	19.725	0	59
5	150	154.2	157	146.9	Severe hypoxia	0.502625	19.725	0	157
5	150	146.2	156.9	184.8	Severe hypoxia	0.4425	20.1875	0	55
5	150	146.2	156.9	184.8	Severe hypoxia	0.4425	20.1875	0	51
5	0	0	0	0	Severe hypoxia	0.518125	20.2	89.88095	168
5	0	0	0	0	Severe hypoxia	0.518125	20.2	100	70
5	0	0	0	0	Severe hypoxia	0.518125	20.2	87.27273	110
5	0	0	0	0	Severe hypoxia	0.518125	20.2	91.34615	208
5	0	0	0	0	Severe hypoxia	0.518125	20.2	98.11321	53
5	0	0	0	0	Severe hypoxia	0.518125	20.2	88.46154	104
6	150	164.1	161.1	145.2	Severe hypoxia	0.039429	22.5	0	275
6	150	164.1	161.1	145.2	Severe hypoxia	0.039429	22.5	0	149
6	150	147.1	169.2	129.3	Severe hypoxia	0.589714	20.55714	0	9
6	150	156.9	164.6	150.7	Severe hypoxia	0.138571	20.58571	0	16
6	150	156.9	164.6	150.7	Severe hypoxia	0.138571	20.58571	0	5
6	0	0	0	0	Severe hypoxia	0.188714	20.15714	69.49153	59
6	0	0	0	0	Severe hypoxia	0.188714	20.15714	90.38462	104
6	150	157.3	154.9	134.6	Severe hypoxia	0.292286	19.08571	0	83
6	0	0	0	0	Severe hypoxia	0.118286	22.95714	91.57303	356
6	0	0	0	0	Severe hypoxia	0.118286	22.95714	28.57143	42
6	0	0	0	0	Severe hypoxia	0.061857	21.12857	100	1
6	0	0	0	0	Severe hypoxia	0.061857	21.12857	100	1
7	40	50	48.1	50.1	Severe hypoxia	0.1945	21.5375	0	3
7	40	50	48.1	50.1	Severe hypoxia	0.1945	21.5375	37.77778	90
7	40	46.1	47.1	45.3	Severe hypoxia	0.07825	20.8125	0	5
7	40	46.1	47.1	45.3	Severe hypoxia	0.07825	20.8125	10.46025	10
7	40	47.8	46.5	44.5	Severe hypoxia	0.06125	22.5875	54.87345	61
7	0	0	0	0	Severe hypoxia	0.096125	20.6125	95.71429	140
7	0	0	0	0	Severe hypoxia	0.096125	20.6125	93.02326	43
7	0	0	0	0	Severe hypoxia	0.0085	21.7	77.77778	27
7	0	0	0	0	Severe hypoxia	0.0085	21.7	100	4
8	116	112.9	119.8	111	Severe hypoxia	0.122625	20.0125	0	145
8	116	112.9	119.8	111	Severe hypoxia	0.122625	20.0125	0	12
8	116	115.8	119.2	105.9	Severe hypoxia	0.32875	20.025	0	19
8	116	115.8	119.2	105.9	Severe hypoxia	0.32875	20.025	0	31
8	116	113.7	119.5	115.6	Severe hypoxia	0.17075	20.2	0	46
8	116	113.7	119.5	115.6	Severe hypoxia	0.17075	20.2	0	10
8	0	0	0	0	Severe hypoxia	0.21125	20.5	33.33333	33
8	0	0	0	0	Severe hypoxia	0.23225	20.1	94.28571	105
8	0	0	0	0	Severe hypoxia	0.23225	20.1	89.47368	19
8	0	0	0	0	Severe hypoxia	0.1195	20.175	57.89474	19
8	0	0	0	0	Severe hypoxia	0.1195	20.175	100	2
9	150	149.2	147.5	120.8	Severe hypoxia	0	27.675	0	197
9	150	149.2	147.5	120.8	Severe hypoxia	0	27.675	0	197
9	150	155.5	151.8	125	Severe hypoxia	0.152375	23.5125	0	206

Table S5. Continued.

9	150	155.5	151.8	125	Severe hypoxia	0.152375	23.5125	0	130
9	150	151.2	157.3	130.1	Severe hypoxia	0	25.6125	0	182
9	150	151.2	157.3	130.1	Severe hypoxia	0	25.6125	0	149
9	0	0	0	0	Severe hypoxia	0.136125	23.1125	88.57143	105
9	0	0	0	0	Severe hypoxia	0.136125	23.1125	61.29032	341
9	0	0	0	0	Severe hypoxia	0.025	23.9125	83.83838	99
9	0	0	0	0	Severe hypoxia	0.025	23.9125	91.20879	91
9	0	0	0	0	Severe hypoxia	0.001875	24.6375	90.29126	103
9	0	0	0	0	Severe hypoxia	0.001875	24.6375	83.88158	304
10	150	142.9	148.1	120.2	Severe hypoxia	0.150875	21.5875	0	169
10	150	142.9	148.1	120.2	Severe hypoxia	0.150875	21.5875	0	207
10	150	146.6	155.1	121.4	Severe hypoxia	0.100125	22.325	0	148
10	150	146.6	155.1	121.4	Severe hypoxia	0.100125	22.325	0	145
10	150	142	142.4	143.7	Severe hypoxia	0.4975	20.6625	0	83
10	150	142	142.4	143.7	Severe hypoxia	0.4975	20.6625	0	464
10	150	147.6	154	126.9	Severe hypoxia	0.0315	26.6375	0	327
10	150	147.6	154	126.9	Severe hypoxia	0.0315	26.6375	0	260
10	0	0	0	0	Severe hypoxia	0.04975	24.225	84.34505	313
10	0	0	0	0	Severe hypoxia	0.04975	24.225	1.449275	69
10	0	0	0	0	Severe hypoxia	0.396125	20.875	97.28507	221
10	0	0	0	0	Severe hypoxia	0.396125	20.875	0	73
10	0	0	0	0	Severe hypoxia	0.16775	22.125	76.14379	306
10	0	0	0	0	Severe hypoxia	0.16775	22.125	95.78947	95
10	0	0	0	0	Severe hypoxia	0.10025	21.625	83.78378	370
11	116	123.6	123.3	102	Severe hypoxia	0.201125	21.1625	0	36
11	116	123.6	123.3	102	Severe hypoxia	0.201125	21.1625	0	112
11	116	124.5	117	107.2	Severe hypoxia	0.308625	20.95	0	60
11	116	124.5	117	107.2	Severe hypoxia	0.308625	20.95	0	153
11	116	114.3	120.5	103.8	Severe hypoxia	0.160225	21.85	0	149
11	116	114.3	120.5	103.8	Severe hypoxia	0.160225	21.85	0	34
11	0	0	0	0	Severe hypoxia	0.08975	21.225	93.45238	168
11	0	0	0	0	Severe hypoxia	0.08975	21.225	90.66667	75
11	0	0	0	0	Severe hypoxia	0.21875	21.4875	64.63415	82
11	0	0	0	0	Severe hypoxia	0.232	21.45	86.44068	59
11	0	0	0	0	Severe hypoxia	0.232	21.45	94.78827	307
12	116	118.2	120.4	102	Severe hypoxia	0.15175	23.1125	0	134
12	116	118.2	120.4	102	Severe hypoxia	0.15175	23.1125	0	271
12	0	0	0	0	Severe hypoxia	0.013875	21.31375	86	50
12	0	0	0	0	Severe hypoxia	0.013875	21.31375	100	14
13	116	112.2	120.1	109	Severe hypoxia	0.457625	21.325	0	136
13	116	112.2	120.1	109	Severe hypoxia	0.457625	21.325	0	6
13	116	112	117	102.5	Severe hypoxia	0.080875	22.7375	0	15
13	116	112	117	102.5	Severe hypoxia	0.080875	22.7375	0	23
13	116	113.7	116.9	104.5	Severe hypoxia	0.10175	24.0625	0	57
13	116	113.7	116.9	104.5	Severe hypoxia	0.10175	24.0625	0	45
13	0	0	0	0	Severe hypoxia	0.03675	23.1625	37.5	8
13	0	0	0	0	Severe hypoxia	0.03675	23.1625	90.90909	99
13	0	0	0	0	Severe hypoxia	0.18325	23.275	100	24
13	0	0	0	0	Severe hypoxia	0.490375	21.3625	87.37864	103
13	0	0	0	0	Severe hypoxia	0.18325	23.275	100	65
14	116	123.9	131.1	113	Severe hypoxia	0.430625	20.7875	0	3
14	116	123.9	131.1	113	Severe hypoxia	0.430625	20.7875	0	2
14	116	129	137	122	Severe hypoxia	0.0915	22.3375	0	2
14	0	0	0	0	Severe hypoxia	0.375375	20.825	83.33333	6
14	0	0	0	0	Severe hypoxia	0.375375	20.825	85.71429	7
14	0	0	0	0	Severe hypoxia	0.05475	22.3375	100	3
15	116	128.5	131.9	119.8	Severe hypoxia	0.072125	24.725	0	55
15	116	128.5	131.9	119.8	Severe hypoxia	0.072125	24.725	0	81
15	116	131.8	134	127.1	Severe hypoxia	0.33	23.45	0	180
15	116	131.8	134	127.1	Severe hypoxia	0.33	23.45	0	31

Table S5. Continued.

15	116	128.7	131.7	116.5	Severe hypoxia	0.206875	21.45	0	16
15	116	128.7	131.7	116.5	Severe hypoxia	0.206875	21.45	0	67
15	0	0	0	0	Severe hypoxia	0.0005	21.7875	100	1
15	0	0	0	0	Severe hypoxia	0.0005	21.7875	81.81818	330
15	0	0	0	0	Severe hypoxia	0.33275	21.1375	78.94737	19
15	0	0	0	0	Severe hypoxia	0.33275	21.1375	96.07843	51
15	0	0	0	0	Severe hypoxia	0	25.1	85.71429	7
15	0	0	0	0	Severe hypoxia	0	25.1	95.2381	21
16	116	124.3	135.3	101.3	Severe hypoxia	0.223625	21.7875	0	32
16	116	124.3	135.3	101.3	Severe hypoxia	0.223625	21.7875	0	38
16	116	121.1	127.1	120.1	Severe hypoxia	0.2595	21.45	0	19
16	116	121.1	127.1	120.1	Severe hypoxia	0.2595	21.45	0	157
16	0	0	0	0	Severe hypoxia	0.081875	22.7875	85.29412	68
16	0	0	0	0	Severe hypoxia	0.081875	22.7875	88.88889	9
16	0	0	0	0	Severe hypoxia	0.257875	21.075	100	10
16	0	0	0	0	Severe hypoxia	0.257875	21.075	98.11321	53
16	0	0	0	0	Severe hypoxia	0.114625	24.5125	95.16129	62
16	0	0	0	0	Severe hypoxia	0.114625	24.5125	77.06422	109
17	150	163.2	154.5	130.5	Severe hypoxia	0.035	23.8	0	4
17	150	163.2	154.5	130.5	Severe hypoxia	0.035	23.8	0	9
17	116	110.8	118.8	112.6	Severe hypoxia	0.048125	24.2625	0	30
17	116	110.8	118.8	112.6	Severe hypoxia	0.048125	24.2625	0	71
17	116	115.9	116	106.8	Severe hypoxia	0.304875	23.0875	0	4
17	116	115.9	116	106.8	Severe hypoxia	0.304875	23.0875	0	19
17	116	124.5	118.4	109.4	Severe hypoxia	0.416375	20.65	0	41
17	116	124.5	118.4	109.4	Severe hypoxia	0.416375	20.65	0	4
17	116	122.9	116.8	111.7	Severe hypoxia	0.4225	20.6875	0	31
17	116	122.9	116.8	111.7	Severe hypoxia	0.4225	20.6875	0	204
18	40	46.1	49.5	46.8	Severe hypoxia	0.046875	22.3875	59.59957	9
18	40	46.1	49.5	46.8	Severe hypoxia	0.046875	22.3875	74.57462	141
18	40	49.9	41.7	37.5	Severe hypoxia	0.0155	21.2625	0	4
18	40	49.9	41.7	37.5	Severe hypoxia	0.0155	21.2625	60.08584	25
18	116	112	133.9	115.4	Severe hypoxia	0.25975	21.3875	0	4
18	116	112	133.9	115.4	Severe hypoxia	0.25975	21.3875	0	2
18	116	114.7	122.8	113.3	Severe hypoxia	0.2885	20.5625	0	4
18	116	114.7	122.8	113.3	Severe hypoxia	0.2885	20.5625	0	5
18	116	122	114.6	97.9	Severe hypoxia	0.04675	21.9375	0	2
18	116	122	114.6	97.9	Severe hypoxia	0.04675	21.9375	0	8
18	0	0	0	0	Severe hypoxia	0.097625	20.9375	100	2
18	0	0	0	0	Severe hypoxia	0.097625	20.9375	100	1
19	40	43.6	41.8	41	Severe hypoxia	0.44325	20.55	0	1
19	40	41.5	44.5	41.4	Severe hypoxia	0.048875	21.1875	100	2
19	116	124.1	118.6	132.2	Severe hypoxia	0.292	21	0	2
19	116	122.1	114.4	111.8	Severe hypoxia	0.527375	20.5375	0	16
19	116	111.8	124.1	134.1	Severe hypoxia	0.303875	21	0	1
19	116	111.8	124.1	134.1	Severe hypoxia	0.303875	21	0	7
19	116	116.6	115.8	117.5	Severe hypoxia	0.577925	20.4875	0	1
19	116	116.6	115.8	117.5	Severe hypoxia	0.577925	20.4875	0	2
19	116	113	115.6	130	Severe hypoxia	0.110375	20.6375	0	3
19	116	115.9	116.8	110.5	Severe hypoxia	0.113875	21.1125	0	27
19	116	115.9	116.8	110.5	Severe hypoxia	0.113875	21.1125	0	18
19	0	0	0	0	Severe hypoxia	0.0635	21	80	40
19	0	0	0	0	Severe hypoxia	0.0635	21	33.33333	3
19	0	0	0	0	Severe hypoxia	0.115875	21.1	100	9
27	116	115.7	138.3	136.4	Severe hypoxia	0.273875	21.2125	0	47
27	116	115.7	138.3	136.4	Severe hypoxia	0.273875	21.2125	0	114
27	0	0	0	0	Severe hypoxia	0.64225	20.675	87.42515	167
27	0	0	0	0	Severe hypoxia	0.64225	20.675	0	1
27	0	0	0	0	Severe hypoxia	0.82875	20.075	93.50649	77
29	40	50.8	42.4	48	Severe hypoxia	0.7765	20.5125	0	2
29	40	50.8	42.4	48	Severe hypoxia	0.7765	20.5125	28.14919	70
29	40	41.6	48.6	47.2	Severe hypoxia	0.826125	20.6125	52.94416	107
29	40	41.6	48.6	47.2	Severe hypoxia	0.826125	20.6125	50.30181	71

Table S5. Continued.

29	116	108.8	118	121.6	Severe hypoxia	1.02025	20.0625	0	8
29	116	108.8	118	121.6	Severe hypoxia	1.02025	20.0625	0	20
29	116	106.8	134.7	152.3	Severe hypoxia	0.61725	20.825	0	78
29	116	106.8	134.7	152.3	Severe hypoxia	0.61725	20.825	0	41
29	116	103.7	123.2	134.4	Severe hypoxia	1.02625	20.075	0	163
29	116	103.7	123.2	134.4	Severe hypoxia	1.02625	20.075	0	141
29	0	0	0	0	Severe hypoxia	1.02325	20.1125	87.69231	65
29	0	0	0	0	Severe hypoxia	1.02325	20.1125	70.58824	68
30	116	121	122.1	125.6	Severe hypoxia	0.63175	20.9125	0	97
30	116	121	122.1	125.6	Severe hypoxia	0.63175	20.9125	0	79
30	0	0	0	0	Severe hypoxia	0.797375	20.5875	80	10
30	0	0	0	0	Severe hypoxia	0.797375	20.5875	87.7551	49
31	30	40.4	41.8	44.7	Severe hypoxia	0.77325	20.4125	51.67292	219
31	30	40.4	41.8	44.7	Severe hypoxia	0.77325	20.4125	40.58509	134
31	80	65.1	76.7	76.4	Severe hypoxia	0.8995	20.3	0	65
31	80	65.1	76.7	76.4	Severe hypoxia	0.8995	20.3	0	42
31	30	38.7	42.3	43.4	Severe hypoxia	0.69875	20.475	64.60483	67
31	30	38.7	42.3	43.4	Severe hypoxia	0.69875	20.475	62.09107	143
31	0	0	0	0	Severe hypoxia	0.75675	20.125	87.61905	210
31	0	0	0	0	Severe hypoxia	0.75675	20.125	97.36842	38
32	80	66.6	80.6	70.1	Severe hypoxia	1.01225	20.975	0	93
32	80	66.6	80.6	70.1	Severe hypoxia	1.01225	20.975	0	140
32	30	36	37.1	33	Severe hypoxia	0.75125	20.6625	51.72083	219
32	30	36	37.1	33	Severe hypoxia	0.75125	20.6625	42.96711	236
32	116	123.2	123.6	146.2	Severe hypoxia	0.782	21.4	0	411
32	116	123.2	123.6	146.2	Severe hypoxia	0.782	21.4	0	122
32	116	121.6	127.7	138.5	Severe hypoxia	0.79525	21.275	0	31
32	116	121.6	127.7	138.5	Severe hypoxia	0.79525	21.275	0	243
32	0	0	0	0	Severe hypoxia	1.119	20.175	92.73743	179
32	0	0	0	0	Severe hypoxia	1.119	20.175	84.61538	52
33	80	86.5	88.1	98.4	Severe hypoxia	0.992	19.8375	0	87
33	80	86.5	88.1	98.4	Severe hypoxia	0.992	19.8375	0	73
33	30	32.8	35.9	36.2	Severe hypoxia	0.922	19.9	39.43031	71
33	30	32.8	35.9	36.2	Severe hypoxia	0.922	19.9	68.01875	135
33	40	42.9	50.9	46.9	Severe hypoxia	1.008625	19.9375	64.20306	75
33	40	42.9	50.9	46.9	Severe hypoxia	1.008625	19.9375	40.54524	58
33	116	128.1	144.1	143.7	Severe hypoxia	0.8135	20.2375	0	145
33	116	128.1	144.1	143.7	Severe hypoxia	0.8135	20.2375	0	88
33	0	0	0	0	Severe hypoxia	0.756125	20.2	97.61905	84
33	0	0	0	0	Severe hypoxia	0.756125	20.2	68.03653	219
34	80	76.6	82.2	76.1	Severe hypoxia	0.2785	21.325	0	23
34	80	76.6	82.2	76.1	Severe hypoxia	0.2785	21.325	0	69
34	30	33.9	42.4	42.5	Severe hypoxia	0.803375	20.9375	37.20065	46
34	30	33.9	42.4	42.5	Severe hypoxia	0.803375	20.9375	0	6
34	40	43.7	47.1	48.6	Severe hypoxia	0.52	20.725	0	1
34	0	0	0	0	Severe hypoxia	0.151	22.5125	70.83333	24
34	0	0	0	0	Severe hypoxia	0.151	22.5125	96.93878	98
35	80	78.1	74.3	86.4	Severe hypoxia	0.373625	21.9	0	52
35	80	78.1	74.3	86.4	Severe hypoxia	0.373625	21.9	0	342
35	30	32.7	32.4	36.6	Severe hypoxia	0.295625	22.6875	18.25484	6
35	80	92.6	88.7	93.1	Severe hypoxia	0.623125	20.875	0	44
35	80	95.6	88.7	93.1	Severe hypoxia	0.623125	20.875	0	10
35	30	32.2	35.6	34.5	Severe hypoxia	0.144875	22.525	47.71561	101
35	30	32.2	35.6	34.5	Severe hypoxia	0.144875	22.525	59.84576	97
35	80	85.8	84	80.1	Severe hypoxia	0.583625	21.0875	0	10
35	80	85.8	84	80.1	Severe hypoxia	0.583625	21.0875	0	19
35	30	40.9	36.4	36.6	Severe hypoxia	0.123625	23.6625	5.215668	21
35	30	40.9	36.4	36.6	Severe hypoxia	0.123625	23.6625	15.647	7

Table S6. Experimental data for *Ceratitis capitata*

Block	Nominal dose	Absorbed dose: bottom	Absorbed dose: middle	Absorbed dose: top	Atmospheric conditions	O_2 (%)	CO_2 (%)	Adult emergence (corrected)	No. insects treated
1	0	0	0	0	Normoxia	21	0	92.85714	70
1	0	0	0	0	Normoxia	21	0	88.0597	134
1	0	0	0	0	Normoxia	21	0	85	60
1	0	0	0	0	Normoxia	21	0	92.0354	113
1	0	0	0	0	Normoxia	21	0	95.12195	123
1	0	0	0	0	Normoxia	21	0	95.76271	118
1	0	0	0	0	Normoxia	21	0	97.65625	128
1	0	0	0	0	Normoxia	21	0	91.09589	146
1	100	95.1	126.4	107.3	Normoxia	21	0	0	23
1	100	95.1	126.4	107.3	Normoxia	21	0	0	42
3	100	100.3	102	91.4	Normoxia	21	0	0	179
3	100	100.3	102	91.4	Normoxia	21	0	0	8
1	30	32.5	43.7	34.4	Normoxia	21	0	1.506409	72
1	30	32.5	43.7	34.4	Normoxia	21	0	0	70
1	30	32.5	43.7	34.4	Normoxia	21	0	0	51
1	30	32.5	43.7	34.4	Normoxia	21	0	0	44
1	30	32.5	43.7	34.4	Normoxia	21	0	12.0511	18
1	30	32.5	43.7	34.4	Normoxia	21	0	0	62
1	30	32.5	43.7	34.4	Normoxia	21	0	0	9
1	30	32.5	43.7	34.4	Normoxia	21	0	0.809402	134
1	30	32.5	43.7	34.4	Normoxia	21	0	5.378175	121
1	30	32.5	43.7	34.4	Normoxia	21	0	0	21
1	30	32.5	43.7	34.4	Normoxia	21	0	0	5
1	100	95.1	126.4	107.3	Normoxia	21	0	0	86
1	100	95.1	126.4	107.3	Normoxia	21	0	0	13
1	100	95.1	126.4	107.3	Normoxia	21	0	0	2
1	100	95.1	126.4	107.3	Normoxia	21	0	0	47
2	30	34.7	32.7	27.2	Normoxia	21	0	2.411613	117
2	30	34.7	32.7	27.2	Normoxia	21	0	7.206614	137
2	0	0	0	0	Normoxia	21	0	30.43478	207
2	0	0	0	0	Normoxia	21	0	85.83333	240
2	0	0	0	0	Normoxia	21	0	81.34328	134
2	0	0	0	0	Normoxia	21	0	80.4878	82
2	0	0	0	0	Normoxia	21	0	78.33333	180
2	0	0	0	0	Normoxia	21	0	58.22454	383
2	0	0	0	0	Normoxia	21	0	44.40154	264
2	0	0	0	0	Normoxia	21	0	84.23423	222
2	0	0	0	0	Normoxia	21	0	91.21622	148
2	0	0	0	0	Normoxia	21	0	74.31193	109
2	30	37.9	38.4	34.8	Normoxia	21	0	2.137026	132
2	30	37.9	38.4	34.8	Normoxia	21	0	0	28
2	30	33.4	32.1	30	Normoxia	21	0	9.248769	427
2	30	33.4	32.1	30	Normoxia	21	0	0	55
2	30	32.7	33.2	29.2	Normoxia	21	0	9.529981	74
2	30	32.7	33.2	29.2	Normoxia	21	0	0	24
2	30	33	35.1	28.1	Normoxia	21	0	49.93473	161
2	30	33	35.1	28.1	Normoxia	21	0	2.979797	142
3	100	102.8	103.9	97.6	Normoxia	21	0	0	30
3	100	102.8	103.9	97.6	Normoxia	21	0	0	129
3	0	0	0	0	Normoxia	21	0	98.3871	62
3	0	0	0	0	Normoxia	21	0	82.35294	17
3	0	0	0	0	Normoxia	21	0	100	16
3	0	0	0	0	Normoxia	21	0	88.70968	62
3	0	0	0	0	Normoxia	21	0	81.66667	120
3	0	0	0	0	Normoxia	21	0	83.33333	12
3	0	0	0	0	Normoxia	21	0	85.29412	34

Table S6. Continued.

3	0	0	0	0	Normoxia	21	0	97.05882	34
3	0	0	0	0	Normoxia	21	0	50	2
3	100	101.7	106.8	98.3	Normoxia	21	0	0	9
3	100	101.7	106.8	98.3	Normoxia	21	0	0	1
3	100	102.2	100.7	89.2	Normoxia	21	0	0	72
3	100	102.2	100.7	89.2	Normoxia	21	0	0	35
4	0	0	0	0	Normoxia	21	0	83.33333	60
4	0	0	0	0	Normoxia	21	0	62.5	40
4	0	0	0	0	Normoxia	21	0	90	10
4	0	0	0	0	Normoxia	21	0	96.66667	30
4	100	118.3	109.6	97.1	Normoxia	21	0	0	56
4	100	118.3	109.6	97.1	Normoxia	21	0	0	10
4	100	105.5	119.8	93.1	Normoxia	21	0	0	19
4	100	105.5	119.8	93.1	Normoxia	21	0	0	45
4	100	104	108.6	104.2	Normoxia	21	0	0	18
4	100	104	108.6	104.2	Normoxia	21	0	0	15
5	0	0	0	0	Normoxia	21	0	92.30769	13
5	0	0	0	0	Normoxia	21	0	100	14
5	0	0	0	0	Normoxia	21	0	64.70588	17
5	0	0	0	0	Normoxia	21	0	61.90476	21
5	0	0	0	0	Normoxia	21	0	90	10
5	100	106.6	108.6	101.8	Normoxia	21	0	0	16
5	100	108.9	111.7	100.8	Normoxia	21	0	0	1
6	0	0	0	0	Normoxia	21	0	100	5
6	0	0	0	0	Normoxia	21	0	86.36364	22
6	0	0	0	0	Normoxia	21	0	100	1
6	0	0	0	0	Normoxia	21	0	83.33333	12
6	100	103.2	111.8	94.9	Normoxia	21	0	0	7
6	100	109.3	114.1	107.1	Normoxia	21	0	0	2
6	100	103.2	111.8	94.9	Normoxia	21	0	0	4
6	100	108.7	111.6	95.3	Normoxia	21	0	0	5
6	100	106.1	111	96.3	Normoxia	21	0	0	2
6	100	106.1	111	96.3	Normoxia	21	0	0	4
7	0	0	0	0	Normoxia	21	0	86.5	104
7	0	0	0	0	Normoxia	21	0	100	4
7	100	105.5	106.6	92.8	Normoxia	21	0	0	18
7	100	107.6	103.1	104	Normoxia	21	0	0	17
7	100	105.4	112.8	106.9	Normoxia	21	0	0	2
7	100	105.4	112.8	106.9	Normoxia	21	0	0	30
8	0	0	0	0	Normoxia	21	0	62.0438	137
8	0	0	0	0	Normoxia	21	0	75	56
8	0	0	0	0	Normoxia	21	0	97	99
8	0	0	0	0	Normoxia	21	0	91.15044	113
8	0	0	0	0	Normoxia	21	0	64.70588	34
8	0	0	0	0	Normoxia	21	0	79.3	92
8	0	0	0	0	Normoxia	21	0	92.10526	38
8	0	0	0	0	Normoxia	21	0	97.19626	107
8	100	97.5	106.7	91.3	Normoxia	21	0	0	53
8	100	97.5	106.7	91.3	Normoxia	21	0	0	32
8	100	106.8	107.4	87.3	Normoxia	21	0	0	62
8	100	106.8	107.4	87.3	Normoxia	21	0	0	65
8	100	101.8	102.9	89.6	Normoxia	21	0	0	52
8	100	101.8	102.9	89.6	Normoxia	21	0	0	10
9	0	0	0	0	Normoxia	21	0	97.14286	35
9	0	0	0	0	Normoxia	21	0	91.42857	70
9	0	0	0	0	Normoxia	21	0	97.2973	111
9	0	0	0	0	Normoxia	21	0	92.47312	93
9	0	0	0	0	Normoxia	21	0	55.55556	18
9	0	0	0	0	Normoxia	21	0	70.21277	94
9	0	0	0	0	Normoxia	21	0	78.26087	23
9	0	0	0	0	Normoxia	21	0	76.4	110

Table S6. Continued.

9	100	105.8	108.6	95.1	Normoxia	21	0	0	44
9	100	105.8	108.6	95.1	Normoxia	21	0	0	116
9	100	100.1	105.6	91.8	Normoxia	21	0	0	78
9	100	100.1	105.6	91.8	Normoxia	21	0	0	87
9	100	102.9	106.9	89.2	Normoxia	21	0	0	102
10	0	0	0	0	Normoxia	21	0	0	83
10	0	0	0	0	Normoxia	21	0	0	6
10	0	0	0	0	Normoxia	21	0	97.05882	34
10	0	0	0	0	Normoxia	21	0	25	4
10	0	0	0	0	Normoxia	21	0	100	8
10	100	96.7	103.4	100.6	Normoxia	21	0	0	98
10	100	104.8	103.5	98.1	Normoxia	21	0	0	32
10	100	104.8	103.5	98.1	Normoxia	21	0	0	41
10	100	95.1	94.9	85.1	Normoxia	21	0	0	2
10	100	95.1	94.9	85.1	Normoxia	21	0	0	2
10	100	96.7	103.4	100.6	Normoxia	21	0	0	2
11	0	0	0	0	Normoxia	21	0	92.77108	83
11	0	0	0	0	Normoxia	21	0	70	10
11	0	0	0	0	Normoxia	21	0	81.81818	11
11	0	0	0	0	Normoxia	21	0	100	20
11	100	100.1	102.4	105	Normoxia	21	0	0	38
11	100	102.5	102.6	97.9	Normoxia	21	0	0	13
11	100	102.5	102.6	97.9	Normoxia	21	0	0	1
11	100	103.8	105.9	86.6	Normoxia	21	0	0	8
11	100	101.4	103.2	81.4	Normoxia	21	0	0	21
11	100	100.1	102.4	105	Normoxia	21	0	0	20
11	100	101.4	103.2	81.4	Normoxia	21	0	0	11
11	100	103.8	105.9	86.6	Normoxia	21	0	0	1
12	30	29.2	27.2	25.3	Normoxia	21	0	2.457123	114
12	30	29.2	27.2	25.3	Normoxia	21	0	0	3
12	0	0	0	0	Normoxia	21	0	0	2
12	0	0	0	0	Normoxia	21	0	71.42857	28
12	100	91.7	102.2	88.7	Normoxia	21	0	0	74
12	100	91.6	101.5	91.7	Normoxia	21	0	0	46
12	100	91.7	102.2	88.7	Normoxia	21	0	0	1
13	30	33.2	31.2	32.2	Normoxia	21	0	0	68
13	30	33.2	31.2	32.2	Normoxia	21	0	0	24
13	30	35.9	35.4	32.5	Normoxia	21	0	0	5
13	30	35.9	35.4	32.5	Normoxia	21	0	10.20304	11
13	0	0	0	0	Normoxia	21	0	89.0625	64
13	100	100.8	102.5	90.6	Normoxia	21	0	0	36
13	100	100.8	102.5	90.6	Normoxia	21	0	0	34
13	100	97.2	102.2	96.8	Normoxia	21	0	0	45
13	100	97.2	102.2	96.8	Normoxia	21	0	0	142
14	0	0	0	0	Normoxia	21	0	40.74074	27
14	0	0	0	0	Normoxia	21	0	50	14
14	100	85.2	104.2	92.2	Normoxia	21	0	0	7
14	100	85.2	104.2	92.2	Normoxia	21	0	0	21
14	100	92.9	104.6	93.2	Normoxia	21	0	0	16
14	100	92.9	104.6	93.2	Normoxia	21	0	0	11
15	30	29.8	35.2	32.2	Normoxia	21	0	0.978349	246
15	30	29.8	35.2	32.2	Normoxia	21	0	1.449843	83
15	0	0	0	0	Normoxia	21	0	83.13253	166
15	100	100	104.8	112.8	Normoxia	21	0	0	83
15	100	100	104.8	112.8	Normoxia	21	0	0	204
16	50	48.9	58	49.1	Normoxia	21	0	0	64
16	30	30.6	32.2	31.7	Normoxia	21	0	0	28
16	30	30.6	32.2	31.7	Normoxia	21	0	1.724138	59
16	0	0	0	0	Normoxia	21	0	97.14286	35
16	0	0	0	0	Normoxia	21	0	100	27
16	100	107.8	126.7	109.7	Normoxia	21	0	0	1
16	100	107.8	126.7	109.7	Normoxia	21	0	0	51

Table S6. Continued.

16	100	101.8	127.5	102.6	Normoxia	21	0	0	29
16	100	101.8	127.5	102.6	Normoxia	21	0	0	36
17	30	25.6	27.2	29.2	Normoxia	21	0	0	22
17	30	25.6	27.2	29.2	Normoxia	21	0	0	23
17	0	0	0	0	Normoxia	21	0	78.94737	19
17	0	0	0	0	Normoxia	21	0	87.09677	31
17	100	117.1	110.3	110.6	Normoxia	21	0	0	110
17	100	117.1	110.3	110.6	Normoxia	21	0	0	84
18	70	76.2	76.7	76.3	Normoxia	21	0	0	67
18	70	76.2	76.7	76.3	Normoxia	21	0	0	60
18	50	49.8	56	52.6	Normoxia	21	0	0	22
18	50	49.8	56	52.6	Normoxia	21	0	0	16
18	0	0	0	0	Normoxia	21	0	67.10526	76
18	0	0	0	0	Normoxia	21	0	100	5
19	20	21.2	19.2	18.8	Normoxia	21	0	27.27273	11
19	50	47.7	50.9	49.6	Normoxia	21	0	0	15
20	20	22.9	24.7	22.6	Normoxia	21	0	36.21701	4
19	70	72.4	70.9	71.8	Normoxia	21	0	0	12
19	70	72.4	70.9	71.8	Normoxia	21	0	0	56
19	50	47.7	50.9	49.6	Normoxia	21	0	0	49
20	20	22.9	24.7	22.6	Normoxia	21	0	0	79
20	70	67.8	75.4	75.3	Normoxia	21	0	0	15
20	70	67.8	75.4	75.3	Normoxia	21	0	0	132
20	50	52.4	50.1	50.8	Normoxia	21	0	0	5
20	50	52.4	50.1	50.8	Normoxia	21	0	0	348
20	0	0	0	0	Normoxia	21	0	84.21053	38
20	0	0	0	0	Normoxia	21	0	53.84615	13
21	20	18	20.1	18.5	Normoxia	21	0	1.639344	61
21	20	18	20.1	18.5	Normoxia	21	0	0	146
21	20	20.7	22.5	20	Normoxia	21	0	4.761905	126
21	20	20.7	22.5	20	Normoxia	21	0	3.921569	102
21	70	64.4	78	69.1	Normoxia	21	0	0	136
21	70	64.4	78	69.1	Normoxia	21	0	0	50
21	70	70.5	79.4	69.9	Normoxia	21	0	0	136
21	70	70.5	79.4	69.9	Normoxia	21	0	0	267
22	0	0	0	0	Normoxia	21	0	16.66667	6
22	0	0	0	0	Normoxia	21	0	72.58065	62
22	0	0	0	0	Normoxia	21	0	74.46809	94
22	50	48.7	56	45.5	Normoxia	21	0	5.087505	36
22	50	48.7	56	45.5	Normoxia	21	0	0	99
22	70	72.4	72.4	67.2	Normoxia	21	0	0	85
22	70	72.4	72.4	67.2	Normoxia	21	0	0	15
23	0	0	0	0	Normoxia	21	0	87.9	58
23	0	0	0	0	Normoxia	21	0	85.9	64
12	0	0	0	0	Hypoxia	4.9125	16.225	62.5	8
12	0	0	0	0	Hypoxia	4.9125	16.225	90.32258	62
12	100	102.3	104.9	112	Hypoxia	5.156	16.300	0	49
12	100	102.3	104.9	112	Hypoxia	5.156	16.300	0	24
12	100	115.1	94.8	99.6	Hypoxia	5.0188	14.85	0	12
13	0	0	0	0	Hypoxia	5.1563	16.138	71.60494	81
13	0	0	0	0	Hypoxia	5.1563	16.138	85.71429	14
13	30	36.1	31.8	31.8	Hypoxia	5.1988	15.375	0	41
13	30	36.1	31.8	31.8	Hypoxia	5.1988	15.375	0	30
13	0	0	0	0	Hypoxia	4.375	16.775	81.13208	53
13	0	0	0	0	Hypoxia	4.375	16.775	71.42857	21
13	30	31.6	34.9	31	Hypoxia	6.1975	14.425	4.156794	27
13	30	31.6	34.9	31	Hypoxia	6.1975	14.425	0	17
13	100	90.9	104	102.1	Hypoxia	5.1988	15.163	0	91
13	100	122.7	115.9	110.1	Hypoxia	5.7838	14.238	0	36
13	100	122.7	115.9	110.1	Hypoxia	5.7838	14.238	0	3

Table S6. Continued.

13	100	104	107.8	109.9	Hypoxia	4.5875	16.675	0	21
13	100	104	107.8	109.9	Hypoxia	4.5875	16.675	0	5
14	0	0	0	0	Hypoxia	5.3388	16.163	69.09091	110
14	0	0	0	0	Hypoxia	5.3388	16.163	67.1875	64
14	100	107.9	93.7	106.3	Hypoxia	4.4013	17.325	0	52
14	100	107.9	93.7	106.3	Hypoxia	4.4013	17.325	0	81
14	100	100.2	107	115	Hypoxia	5.3188	15.263	0	21
14	100	100.2	107	115	Hypoxia	5.3188	15.263	0	5
15	50	46.8	57.4	55.6	Hypoxia	5.4375	14.55	0	158
15	50	46.8	57.4	55.6	Hypoxia	5.4375	14.55	0	71
15	30	34.3	30.6	36.7	Hypoxia	5.005	14.463	54.15162	40
15	30	34.3	30.6	36.7	Hypoxia	5.005	14.463	19.62943	141
15	0	0	0	0	Hypoxia	4.81	13.125	80.55556	108
15	0	0	0	0	Hypoxia	4.81	13.125	70.347	317
15	30	34.5	35.6	36	Hypoxia	5.1113	15.7	0	1
15	30	34.5	35.6	36	Hypoxia	5.1113	15.7	16.04493	30
15	100	107.8	102.1	102.8	Hypoxia	4.9388	15.763	0	68
15	100	107.8	102.1	102.8	Hypoxia	4.9388	15.763	0	162
15	100	108.6	101.5	108.2	Hypoxia	4.8788	14.088	0	121
15	100	108.6	101.5	108.2	Hypoxia	4.8788	14.088	0	98
15	100	106.6	109.9	114.3	Hypoxia	5.2325	14.075	0	167
15	100	106.6	109.9	114.3	Hypoxia	5.2325	14.075	0	209
15	100	97	122.8	126.6	Hypoxia	5.3775	14.963	0	138
15	100	97	122.8	126.6	Hypoxia	5.3775	14.963	0	120
15	100	100	115.5	114.3	Hypoxia	6.34	14.313	0	94
15	100	100	115.5	114.3	Hypoxia	6.34	14.313	0	42
15	100	117.3	119.1	113.2	Hypoxia	4.7725	15.188	0	99
15	100	117.3	119.1	113.2	Hypoxia	4.7725	15.188	0	137
16	50	55.5	52.2	49.2	Hypoxia	4.8213	16.3	0	50
16	50	55.5	52.2	49.2	Hypoxia	4.8213	16.3	0	29
16	0	0	0	0	Hypoxia	5.74	14.586	92.30769	13
16	0	0	0	0	Hypoxia	5.74	14.586	75	8
16	30	31.3	34.8	36.2	Hypoxia	4.9325	15.65	0	4
16	30	31.3	34.8	36.2	Hypoxia	4.9325	15.65	9.219989	33
16	100	112.9	109.6	123.4	Hypoxia	4.17	16.35	0	82
16	100	112.9	109.6	123.4	Hypoxia	4.17	16.35	0	12
16	100	95.8	105.9	113.5	Hypoxia	5.1413	15.438	0	13
16	100	95.8	105.9	113.5	Hypoxia	5.1413	15.438	0	43
17	0	0	0	0	Hypoxia	5.4488	14.963	95.2381	21
17	30	32	35.7	33.1	Hypoxia	4.8825	15.85	0	3
17	30	32	35.7	33.1	Hypoxia	4.8825	15.85	13.02194	37
17	100	108.5	114.5	114.3	Hypoxia	4.7388	17.063	0	7
17	100	108.5	114.5	114.3	Hypoxia	4.7388	17.063	0	16
18	20	19.2	21.3	19.2	Hypoxia	5.4788	15.275	0	12
18	20	19.2	21.3	19.2	Hypoxia	5.4788	15.275	15.73911	38
18	70	74.7	87.6	83.4	Hypoxia	5.435	16.013	0	20
18	70	74.7	87.6	83.4	Hypoxia	5.435	16.013	0	88
19	20	21.3	24.3	21.5	Hypoxia	6.0588	14.65	9.836066	61
19	20	21.3	24.3	21.5	Hypoxia	6.0588	14.65	33.33333	18
19	70	70.6	68.9	76.3	Hypoxia	5.81	15.863	0	49
19	70	70.6	68.9	76.3	Hypoxia	5.81	15.863	0	33
19	50	47.8	55.8	48.7	Hypoxia	6.67	14.038	0	1
19	50	47.8	55.8	48.7	Hypoxia	6.67	14.038	0	26
20	20	20.8	22.2	20.4	Hypoxia	5.2113	15.825	28.98551	5
20	20	20.8	22.2	20.4	Hypoxia	5.2113	15.825	18.11594	8
20	70	61.2	72	69.3	Hypoxia	5.25	15.7	0	277
20	70	61.2	72	69.3	Hypoxia	5.25	15.7	0	156
20	20	23.6	22.6	21.3	Hypoxia	4.3138	16.925	80.5153	90
20	20	23.6	22.6	21.3	Hypoxia	4.3138	16.925	36.23188	20
20	50	52.3	52.6	56.5	Hypoxia	5.1438	16.588	0	41
20	50	52.3	52.6	56.5	Hypoxia	5.1438	16.588	3.813883	38
20	70	70.5	77.6	70.2	Hypoxia	6.6613	13.313	0	23
20	70	70.5	77.6	70.2	Hypoxia	6.6613	13.313	0	2

Table S6. Continued.

20	50	56.9	59.8	48.9	Hypoxia	4.15	16.725	0	105
20	50	56.9	59.8	48.9	Hypoxia	4.15	16.725	0	169
21	20	21	19.4	15.5	Hypoxia	4.53	16.65	34.48276	145
21	20	21	19.4	15.5	Hypoxia	4.53	16.65	4.545455	22
21	70	71.2	77	71.2	Hypoxia	5.0988	16.175	0	396
21	70	71.2	77	71.2	Hypoxia	5.0988	16.175	0	237
21	20	20.1	20.7	16.8	Hypoxia	4.6363	17.775	5.990783	217
21	20	20.1	20.7	16.8	Hypoxia	4.6363	17.775	0	188
21	70	71.2	77	71.2	Hypoxia	5.0988	16.175	0	396
21	70	71.2	77	71.2	Hypoxia	5.0988	16.175	0	237
21	50	50.6	52.9	50.5	Hypoxia	5.2638	15.675	0	284
21	50	50.6	52.9	50.5	Hypoxia	5.2638	15.675	0	5
21	70	74.9	75.7	70.7	Hypoxia	5.4988	15.838	0	205
21	70	74.9	75.7	70.7	Hypoxia	5.4988	15.838	0	264
21	50	50.8	57.5	52.2	Hypoxia	5.38	16.313	0	43
21	50	50.8	57.5	52.2	Hypoxia	5.38	16.313	0	82
22	0	0	0	0	Hypoxia	5.5525	16.313	75	52
22	0	0	0	0	Hypoxia	5.5525	16.313	58.82353	68
22	20	23.6	25.2	18.6	Hypoxia	5.2238	16.65	86.23286	51
22	20	23.6	25.2	18.6	Hypoxia	5.2238	16.65	15.69859	35
22	20	21	21.7	19.1	Hypoxia	5.2775	15.375	0	5
22	20	21	21.7	19.1	Hypoxia	5.2775	15.375	30.52503	6
22	30	30.4	35.5	24.9	Hypoxia	5.6788	15.425	91.57509	2
22	30	30.4	35.5	24.9	Hypoxia	5.6788	15.425	0	97
22	50	54.8	54	49.5	Hypoxia	5.3688	15.425	0	34
22	50	54.8	54	49.5	Hypoxia	5.3688	15.425	0	7
22	70	73.6	65.2	71.2	Hypoxia	5.54	14.913	0	36
22	70	73.6	65.2	71.2	Hypoxia	5.54	14.913	0	51
22	70	67.8	72.1	70.1	Hypoxia	5.77	15.5	0	40
22	70	67.8	72.1	70.1	Hypoxia	5.77	15.5	0	18
23	0	0	0	0	Hypoxia	4.6313	15.075	50	4
23	20	23.4	27.8	23.5	Hypoxia	4.7788	15.425	86.3061	4
23	20	23.4	27.8	23.5	Hypoxia	4.7788	15.425	21.19799	38
23	30	33.7	38.5	37.5	Hypoxia	4.7213	15.763	0	14
23	30	33.7	38.5	37.5	Hypoxia	4.7213	15.763	0	4
23	20	22.3	26.1	24.7	Hypoxia	4.7725	15.338	23.01496	5
23	20	22.3	26.1	24.7	Hypoxia	4.7725	15.338	51.14435	9
23	30	35.6	37.5	35.3	Hypoxia	4.6675	16.304	0	22
23	70	73.1	77	77.1	Hypoxia	5.075	15.238	0	7
23	70	73.1	77	77.1	Hypoxia	5.075	15.238	0	80
23	50	60.2	58.3	58.2	Hypoxia	4.835	16.675	0	10
23	50	60.2	58.3	58.2	Hypoxia	4.835	16.675	0	10
23	70	70.5	75	71.8	Hypoxia	4.9888	15.1	0	66
23	70	70.5	75	71.8	Hypoxia	4.9888	15.1	0	46
23	50	59.1	62.2	62.7	Hypoxia	4.5788	16.925	0	6
23	50	59.1	62.2	62.7	Hypoxia	4.5788	16.925	0	79
23	100	95.8	117.7	115.4	Hypoxia	5.5975	14.963	0	9
23	100	95.8	117.7	115.4	Hypoxia	5.5975	14.963	0	6
23	100	98.8	107	105.1	Hypoxia	4.7163	15.938	0	30
23	100	98.8	107	105.1	Hypoxia	4.7163	15.938	0	18
23	100	91.8	106.1	106.2	Hypoxia	5.1725	15.313	0	6
23	100	91.8	106.1	106.2	Hypoxia	5.1725	15.313	0	41
2	30	30.7	35.9	34.6	Severe hypoxia	0.3365	21.438	3.358184	84
2	30	30.7	35.9	34.6	Severe hypoxia	0.3365	21.438	6.653006	106
2	30	32.3	34.6	27.2	Severe hypoxia	0.2931	20.2	28.84985	44
2	30	32.3	34.6	27.2	Severe hypoxia	0.2931	20.2	0	26
2	30	34.9	37.1	29	Severe hypoxia	1.865	18.9	0	13
2	30	34.9	37.1	29	Severe hypoxia	1.865	18.9	18.39701	46
2	30	34.3	32.8	31.4	Severe hypoxia	0.1968	20.85	0	37

Table S6. Continued.

2	30	34.3	32.8	31.4	Severe hypoxia	0.1968	20.85	0	104
2	30	33.9	37	26.2	Severe hypoxia	0.1689	21.738	37.17431	129
2	30	33.9	37	26.2	Severe hypoxia	0.1689	21.738	81.80536	100
2	0	0	0	0	Severe hypoxia	0.1953	20.813	80.43478	92
2	0	0	0	0	Severe hypoxia	0.1953	20.813	88.09524	42
2	0	0	0	0	Severe hypoxia	0.1953	20.813	94.96855	159
2	0	0	0	0	Severe hypoxia	1.9208	19.938	88.23529	85
2	0	0	0	0	Severe hypoxia	1.9208	19.938	82.45614	57
2	0	0	0	0	Severe hypoxia	1.9208	19.938	100	15
3	100	102.3	104.2	100.5	Severe hypoxia	0.060	19.688	0	38
3	100	102.3	104.2	100.5	Severe hypoxia	0.060	19.688	0	29
3	100	102.2	105.1	95.5	Severe hypoxia	0.1149	20.288	0	48
3	100	102.2	105.1	95.5	Severe hypoxia	0.1149	20.288	0	91
3	100	101.9	107.7	95.6	Severe hypoxia	0.1546	20.35	0	13
3	100	102.9	107.8	96.6	Severe hypoxia	0.1546	20.35	0	38
3	100	99.7	102.3	89.7	Severe hypoxia	0.008	20.438	0	70
3	100	99.7	102.3	89.7	Severe hypoxia	0.008	20.438	0	29
3	0	0	0	0	Severe hypoxia	0.0926	20.625	98.64865	148
3	0	0	0	0	Severe hypoxia	0.0926	20.625	92	50
3	0	0	0	0	Severe hypoxia	0.0046	20.388	66.66667	3
3	0	0	0	0	Severe hypoxia	0.0046	20.388	95.2381	42
3	0	0	0	0	Severe hypoxia	0.0594	20.613	100	45
3	0	0	0	0	Severe hypoxia	0.0594	20.613	91.74312	109
3	0	0	0	0	Severe hypoxia	0.0911	20.788	94.89796	98
3	0	0	0	0	Severe hypoxia	0.0911	20.788	89.15663	83
4	100	103.4	112.1	88	Severe hypoxia	0.0751	21.738	0	64
4	100	103.4	112.1	88	Severe hypoxia	0.0751	21.738	0	4
4	100	103	110.9	90.2	Severe hypoxia	0.0318	24.413	0	29
4	100	103	110.9	90.2	Severe hypoxia	0.0318	24.413	0	10
4	100	103.1	107.5	90.8	Severe hypoxia	0.0245	24.113	0	24
4	100	103.1	107.5	90.8	Severe hypoxia	0.0245	24.113	0	12
4	0	0	0	0	Severe hypoxia	0.0419	22.875	88.37209	43
4	0	0	0	0	Severe hypoxia	0.0419	22.875	87.5	24
4	0	0	0	0	Severe hypoxia	0.1346	22.013	70.37037	27
4	0	0	0	0	Severe hypoxia	0.1346	22.013	87.5	32
4	0	0	0	0	Severe hypoxia	0.152	21.513	81.81818	22
5	100	104.7	109.6	99.7	Severe hypoxia	0.1568	21.325	0	2
5	100	104.7	109.6	99.7	Severe hypoxia	0.1568	21.325	0	2
5	100	119.6	112.4	99.8	Severe hypoxia	0.2081	20.888	0	6
5	0	0	0	0	Severe hypoxia	0.0909	21.613	42.85714	7
5	0	0	0	0	Severe hypoxia	0.1136	21.575	75	4
5	0	0	0	0	Severe hypoxia	0.0474	21.038	100	4
5	0	0	0	0	Severe hypoxia	0.0474	21.038	0	1
6	100	111.8	115.6	97	Severe hypoxia	0.298	20.938	0	8
6	100	109.3	114.1	107.1	Severe hypoxia	0.6278	20.613	0	4
6	100	108.5	114.4	105.6	Severe hypoxia	0.057	23.2	0	3
6	0	0	0	0	Severe hypoxia	0.2218	20.85	100	2
6	0	0	0	0	Severe hypoxia	0.2218	20.85	88.88889	9
6	0	0	0	0	Severe hypoxia	0.1068	21.338	62.5	8
6	0	0	0	0	Severe hypoxia	0.1068	21.338	66.66667	3
6	0	0	0	0	Severe hypoxia	0.363	20.588	100	2
7	100	103.3	108	103.4	Severe hypoxia	0.09	20.763	0	32
7	100	110.9	109.9	103.1	Severe hypoxia	0.0394	21.313	0	41
7	100	107.3	105.2	99.8	Severe hypoxia	0.2555	20.538	0	2
7	100	107.3	105.2	99.8	Severe hypoxia	0.2555	20.538	0	2
7	0	0	0	0	Severe hypoxia	0.1219	21.763	94.23077	52
7	0	0	0	0	Severe hypoxia	0.205	21.063	89.47368	19
8	100	92.5	108.2	99	Severe hypoxia	0.1143	22.75	0	67
8	100	92.5	108.2	99	Severe hypoxia	0.1143	22.75	0	52
8	100	96	105.3	113.6	Severe hypoxia	0.0648	22.788	0	43
8	100	96	105.3	113.6	Severe hypoxia	0.0648	22.788	0	67
8	100	97.5	106.7	91.3	Severe hypoxia	0.38	22.063	0	76
8	100	97.5	106.7	91.3	Severe hypoxia	0.38	22.063	0	180
8	0	0	0	0	Severe hypoxia	0.1741	23.275	87.04663	193

Table S6. Continued.

8	0	0	0	0	Severe hypoxia	0.338	21.075	69.64286	56
8	0	0	0	0	Severe hypoxia	0.338	21.075	9.615385	52
8	0	0	0	0	Severe hypoxia	0.1523	22.938	90.90909	99
8	0	0	0	0	Severe hypoxia	0.1523	22.938	89.42308	104
9	100	107.3	112.7	93.7	Severe hypoxia	0.2151	23.216	0	5
9	100	107.3	112.7	93.7	Severe hypoxia	0.2151	23.216	0	59
9	100	117.3	112.8	111.6	Severe hypoxia	0.1284	22.914	0	78
9	100	117.3	112.8	111.6	Severe hypoxia	0.1284	22.914	0	37
9	100	110.3	117.4	89.2	Severe hypoxia	0.0409	23.286	0	5
9	100	110.3	117.4	89.2	Severe hypoxia	0.0409	23.286	0	1
9	0	0	0	0	Severe hypoxia	0.1789	22.929	42.26804	97
9	0	0	0	0	Severe hypoxia	0.1789	22.929	66.66667	3
9	0	0	0	0	Severe hypoxia	0.1283	22.914	95.2381	84
9	0	0	0	0	Severe hypoxia	0.1283	22.914	89.74359	39
9	0	0	0	0	Severe hypoxia	0.3643	21.329	63.63636	33
9	0	0	0	0	Severe hypoxia	0.3643	21.329	69.23077	13
9	0	0	0	0	Severe hypoxia	0.1093	23.457	93.10345	58
9	0	0	0	0	Severe hypoxia	0.1093	23.457	83.11688	77
10	30	27.9	35.8	29.3	Severe hypoxia	0.3081	23.775	0	5
10	30	27.9	35.8	29.3	Severe hypoxia	0.3081	23.775	0	1
10	30	26.6	27	30.9	Severe hypoxia	0.1238	22.588	0	4
10	30	26.6	27	30.9	Severe hypoxia	0.1238	22.588	62.61261	36
10	30	33.1	34.7	29.6	Severe hypoxia	0.7745	20.725	0	7
10	30	33.1	34.7	29.6	Severe hypoxia	0.7745	20.725	0	1
10	100	94.1	109.9	91.1	Severe hypoxia	0.3353	23.963	0	9
10	100	110.5	102.3	94.5	Severe hypoxia	0.3065	21.45	0	4
10	100	110.5	102.3	94.5	Severe hypoxia	0.3065	21.45	0	17
10	100	102.8	99.4	90.9	Severe hypoxia	0.3069	21.725	0	4
10	100	98.3	107.9	89.6	Severe hypoxia	0.422	20.938	0	24
10	0	0	0	0	Severe hypoxia	0.4645	23.3	100	3
10	0	0	0	0	Severe hypoxia	0.1046	23.225	100	3
10	0	0	0	0	Severe hypoxia	0.1046	23.225	71.42857	7
11	30	35.1	36.7	30.2	Severe hypoxia	0	24.5	46.43209	30
11	30	35.1	36.7	30.2	Severe hypoxia	0	24.5	14.518	8
11	30	33.4	34.2	31.4	Severe hypoxia	0.0195	22.775	8.934155	13
11	30	33.4	34.2	31.4	Severe hypoxia	0.0195	22.775	39.48897	106
11	100	106.9	103.2	85.6	Severe hypoxia	0.2114	21.588	0	11
11	100	106.9	103.2	85.6	Severe hypoxia	0.2114	21.588	0	101
11	100	101.2	106.1	86.5	Severe hypoxia	0.327	21.288	0	2
11	100	101.2	106.1	86.5	Severe hypoxia	0.327	21.288	0	1
11	100	97.6	106.2	90.2	Severe hypoxia	0.4646	21.05	0	6
11	100	97.6	106.2	90.2	Severe hypoxia	0.4646	21.05	0	7
11	100	101.1	112.9	115.3	Severe hypoxia	0.2441	21.425	0	4
11	100	101.1	112.9	115.3	Severe hypoxia	0.2441	21.425	0	5
11	100	107.3	104.1	76	Severe hypoxia	0.2125	21.925	0	4
11	100	107.3	104.1	76	Severe hypoxia	0.2125	21.925	0	14
11	0	0	0	0	Severe hypoxia	0.0323	22.5	100	21
14	100	95.3	105	97.1	Severe hypoxia	0.6601	21.488	0	21
14	100	95.3	105	97.1	Severe hypoxia	0.6601	21.488	0	10
14	100	101	90.5	103	Severe hypoxia	0.937	20.538	0	4
14	100	101	90.5	103	Severe hypoxia	0.937	20.538	0	8
14	0	0	0	0	Severe hypoxia	0.6288	21.688	0	24
14	0	0	0	0	Severe hypoxia	0.6288	21.688	0	12
17	30	27.9	30.2	31.7	Severe hypoxia	0.4121	21.463	32.42963	26
17	30	27.9	30.2	31.7	Severe hypoxia	0.4121	21.463	77.43401	14
17	100	102.7	128.6	114.4	Severe hypoxia	0.9953	20.15	0	12
17	100	102.7	128.6	114.4	Severe hypoxia	0.9953	20.15	0	11
17	0	0	0	0	Severe hypoxia	0.6353	21.338	75.92593	108

Table S6. Continued.

17	0	0	0	0	Severe hypoxia	0.6353	21.338	63.63636	11
18	20	22.4	21.9	20.7	Severe hypoxia	0.5963	22.175	47.87402	10
18	20	22.4	21.9	20.7	Severe hypoxia	0.5963	22.175	57.8793	31
18	50	55.9	56.4	64.7	Severe hypoxia	0.5779	20.8	0	17
18	50	55.9	56.4	64.7	Severe hypoxia	0.5779	20.8	0	34
18	70	69.7	71.4	77.8	Severe hypoxia	0.5035	20.525	0	8
18	70	69.7	71.4	77.8	Severe hypoxia	0.5035	20.525	0	4
18	20	21.9	24.8	21.2	Severe hypoxia	0.551	21	79.74482	90
18	20	21.9	24.8	21.2	Severe hypoxia	0.551	21	85.44087	7
18	50	53.9	54.8	57.7	Severe hypoxia	0.6391	21.288	0	14
18	50	53.9	54.8	57.7	Severe hypoxia	0.6391	21.288	0	50
18	70	66	80.8	76.1	Severe hypoxia	0.6731	20.613	0	52
18	70	66	80.8	76.1	Severe hypoxia	0.6731	20.613	0	26
19	20	21.5	21.7	21.1	Severe hypoxia	0.5714	21.7	73.91304	23
19	20	21.5	21.7	21.1	Severe hypoxia	0.5714	21.7	72.72727	44
19	50	50.3	55.1	56.7	Severe hypoxia	0.907	20.813	0	40
19	50	50.3	55.1	56.7	Severe hypoxia	0.907	20.813	0	12
19	70	63.2	75.2	84.3	Severe hypoxia	0.422	21.288	0	62
19	70	63.2	75.2	84.3	Severe hypoxia	0.422	21.288	0	3
19	20	18.8	21.5	18.4	Severe hypoxia	0.3815	21.488	58.97436	39
19	20	18.8	21.5	18.4	Severe hypoxia	0.3815	21.488	73.41772	79
19	50	49	51.2	55.1	Severe hypoxia	0.5106	21.163	0	1
19	50	49	51.2	55.1	Severe hypoxia	0.5106	21.163	0	6
19	70	74.6	80.4	71.7	Severe hypoxia	0.7418	21.275	0	5
19	70	74.6	80.4	71.7	Severe hypoxia	0.7418	21.275	0	1
19	30	33	40.3	38.4	Severe hypoxia	0.692	20.725	0	15
19	30	33	40.3	38.4	Severe hypoxia	0.692	20.725	17.34694	98
20	20	22	22.8	20.7	Severe hypoxia	0.5057	22.013	100	260
20	20	22	22.8	20.7	Severe hypoxia	0.5057	22.013	100	5
20	50	53.2	49.6	57.2	Severe hypoxia	0.6559	20.675	0	22
20	50	53.2	49.6	57.2	Severe hypoxia	0.6559	20.675	0	44
20	70	72.5	70.6	77.3	Severe hypoxia	0.777	21.4	0	128
20	70	72.5	70.6	77.3	Severe hypoxia	0.777	21.4	0	67
20	20	21.5	21.1	20.6	Severe hypoxia	0.6828	20.638	20.70393	7
20	20	21.5	21.1	20.6	Severe hypoxia	0.6828	20.638	100	62
20	50	52.6	57.9	54.3	Severe hypoxia	0.7885	20.613	0	74
20	50	52.6	57.9	54.3	Severe hypoxia	0.7885	20.613	0	20
20	70	75.2	81.7	74.2	Severe hypoxia	0.5201	21.175	0	85
20	70	75.2	81.7	74.2	Severe hypoxia	0.5201	21.175	0	96
21	20	18.7	19.7	19.5	Severe hypoxia	0.3053	21.013	9.52381	252
21	20	18.7	19.7	19.5	Severe hypoxia	0.3053	21.013	44.68085	47
21	50	48.2	52.9	52.8	Severe hypoxia	0.4081	20.775	0	24
21	50	48.2	52.9	52.8	Severe hypoxia	0.4081	20.775	0	161
21	70	68.6	84.9	74.6	Severe hypoxia	0.3841	21.163	0	111
21	70	68.6	84.9	74.6	Severe hypoxia	0.3841	21.163	0	318
21	20	20.5	20.5	14.7	Severe hypoxia	0.7381	20.613	77.19298	228
21	20	20.5	20.5	14.7	Severe hypoxia	0.7381	20.613	25	8
21	50	52.2	56.5	56.2	Severe hypoxia	0.3611	21.175	0	107
21	50	52.2	56.5	56.2	Severe hypoxia	0.3611	21.175	0	50
21	70	71.7	72.6	71.9	Severe hypoxia	0.6894	21.763	0	110
21	70	71.7	72.6	71.9	Severe hypoxia	0.6894	21.763	0	181
22	0	0	0	0	Severe hypoxia	0.4314	22.188	100	20
22	0	0	0	0	Severe hypoxia	0.4314	22.188	55.17241	58
22	20	22.6	20.3	17.7	Severe hypoxia	0.8164	20.963	79.20008	37
22	20	22.6	20.3	17.7	Severe hypoxia	0.8164	20.963	58.60806	25
22	20	25.3	21.6	16.8	Severe hypoxia	0.4256	20.963	81.40008	27
22	20	25.3	21.6	16.8	Severe hypoxia	0.4256	20.963	100	39
22	50	52.1	46.6	55	Severe hypoxia	0.6594	20.413	0	171
22	50	52.1	46.6	55	Severe hypoxia	0.6594	20.413	0	9
22	50	53.2	56.6	44.9	Severe hypoxia	0.403	21.075	0	23
22	50	53.2	56.6	44.9	Severe hypoxia	0.403	21.075	0	1
22	70	72.7	72.8	74.7	Severe hypoxia	0.3665	20.988	0	54
22	70	72.3	78.4	71.5	Severe hypoxia	0.708	20.388	0	10
22	70	72.3	78.4	71.5	Severe hypoxia	0.708	20.388	0	13

Table S7. Percentages [mean ± SE (total number of individuals)] of dead insects dissected from puparia (CAL, CCP, PCP, PHA, PEA) and emerged adults (DFA, FFA) of *Anastrepha fraterculus* after irradiation of third instars at different nominal doses and atmospheric conditions

Dose (Gy)	Atmospheric conditions[1]	n	CAL[2, 3]	CCP [2, 3]	PCP [2, 3]	PHA [2, 3]	PEA[2, 3]	DFA[2, 3]	FFA[2, 3]
0	Normoxia	10	15.6 ± 4.2 (95) A	1.2 ± 0.6 (11) A	0.7 ± 0.3 (8) A	5.4 ± 1.5 (54) A	2.7 ± 1.6 (9) A	1.9 ± 0.9 (22) A	72.6 ± 5.3 (763) A
0	Hypoxia	5	22.0 ± 8.3 (101) A	0.9 ± 0.7 (6) A	2.0 ± 0.8 (9) A	3.2 ± 1.1 (25) A	0.7 ± 0.3 (7) A	1.3 ± 0.7 (22) A	69.9 ± 9.6 (701) A
0	Severe hypoxia	6	10.3 ± 3.3 (57) A	1.1 ± 0.5 (6) A	1.1 ± 0.7 (7) A	5.8 ± 2.4 (29) A	2.0 ± 0.4 (14) A	1.5 ± 0.6 (17) A	78.2 ± 5.6 (906) A
25	Normoxia	5	92.8 ± 2.6 (1160) A	0.2 ± 0.4 (3) A	1.0 ± 0.8 (4) A	1.7 ± 0.7 (27) A	0.4 ± 0.2 (7) A	0.7 ± 0.4 (18) A	3.2 ± 1.5 (66) A
25	Hypoxia	5	68.4 ± 11.9 (621) B	1.9 ± 1.1 (9) B	1.7 ± 1.0 (9) AB	13.9 ± 7.0 (73) B	3.3 ± 1.7 (20) A	3.1 ± 1.1 (21) B	7.6 ± 3.4 (53) A
25	Severe hypoxia	6	27.0 ± 4.0 (292) C	1.1 ± 0.4 (12) AB	3.9 ± 0.8 (38) B	20.2 ± 2.1 (247) B	9.9 ± 2.1 (119) B	5.9 ± 1.1 (71) C	32.0 ± 4.4 (426) B
35	Normoxia	5	97.5 ± 1.5 (1917) A	0.0 ± 0.0 (0) A	0.9 ± 0.6 (18) A	0.9 ± 0.6 (20) A	0.2 ± 0.1 (5) A	0.2 ± 0.1 (5) A	0.3 ± 1.5 (7) A
35	Hypoxia	5	85.0 ± 13.0 (963) A	0.8 ± 0.5 (10) AB	1.3 ± 0.8 (21) A	2.1 ± 1.6 (15) A	0.4 ± 0.4 (1) A	0.4 ± 0.4 (1) A	10.0 ± 10.0 (24) A
35	Severe hypoxia	5	34.6 ± 2.1 (333) B	2.7 ± 1.4 (28) B	7.6 ± 2.2 (87) B	37.2 ± 3.9 (416) B	4.9 ± 0.8 (40) B	1.2 ± 0.8 (22) A	11.7 ± 6.5 (56) A
50	Normoxia	5	100.0 ± 0.0 (734) A	0.0 ± 0.0 (0) A	0.0 ± 0.0 (0) A	0.0 ± 0.0 (0) A	0.0 ± 0.0 (0) A	0.0 ± 0.0 (0) A	0.0 ± 0.0 (0) A
50	Hypoxia	5	100.0 ± 0.0 (640) A	0.0 ± 0.0 (0) A	0.0 ± 0.0 (0) A	0.0 ± 0.0 (0) A	0.0 ± 0.0 (0) A	0.0 ± 0.0 (0) A	0.0 ± 0.0 (0) A
50	Severe hypoxia	5	98.1 ± 0.9 (626) A	0.5 ± 0.4 (2) A	0.7 ± 0.7 (7) A	0.8 ± 0.8 (1) A	0.0 ± 0.0 (0) A	0.0 ± 0.0 (0) A	0.0 ± 0.0 (0) A
70	Normoxia	13	100 ± 0.0 (2877) A	0.0 ± 0.0 (0) A	0.0 ± 0.0 (0) A	0.0 ± 0.0 (0) A	0.0 ± 0.0 (0) A	0.0 ± 0.0 (0) A	0.0 ± 0.0 (0) A
70	Hypoxia	6	100.0 ± 0.0 (1151) A	0.0 ± 0.0 (0) A	0.0 ± 0.0 (0) A	0.0 ± 0.0 (0) A	0.0 ± 0.0 (0) A	0.0 ± 0.0 (0) A	0.0 ± 0.0 (0) A
70	Severe hypoxia	10	100.0 ± 0.0 (2188) A	0.0 ± 0.0 (0) A	0.0 ± 0.0 (0) A	0.0 ± 0.0 (0) A	0.0 ± 0.0 (0) A	0.0 ± 0.0 (0) A	0.0 ± 0.0 (0) A

[1] Normoxia (~21.0% O_2, 0.0% CO_2), hypoxia (5.5 ± 0.1% O_2, 15.7 ± 0.2% CO_2) and severe hypoxia (0.3 ± 0.02% O_2, 22.2 ± 0.2% CO_2). [2] CAL (coarctate larvae), CCP (cryptocephalic pupae), PCP (phanerocephalic pupae), PHA (pharate adults), PEA (partially emerged adults), DFA (deformed adults), FFA (fully formed adults). [3] Different letters indicate significant differences between groups (estimated marginal means contrasts, $P < 0.05$)

Table S8. Percentages [mean ± SE (total number of individuals)] of dead insects dissected from puparia (CAL, CCP, PCP, PHA, PEA) and emerged adults (DFA, FFA) of *Anastrepha ludens* after irradiation of third instars at different nominal doses and atmospheric conditions

Dose (Gy)	Atmospheric conditions[1]	n	CAL[2, 3]	CCP [2, 3]	PCP [2, 3]	PHA [2, 3]	PEA[2, 3]	DFA[2, 3]	FFA[2, 3]
0	Normoxia	6	1.3 ± 0.8 (3) A	0.3 ± 0.3 (1) A	0.6 ± 0.6 (2) A	1.6 ± 1.0 (4) A	5.2 ± 3.8 (7) A	1.36 ± 0.9 (2) A	89.7 ± 3.6 (161) A
0	Hypoxia	5	2.9 ± 1.8 (14) A	0.0 ± 0.0 (0) A	0.2 ± 0.1 (3) A	0.7 ± 0.4 (6) A	1.4 ± 0.6 (21) B	1.2 ± 0.5 (16) A	93.6 ± 1.8 (1062) A
0	Anoxia	6	3.9 ± 3.3 (27) A	0.3 ± 0.3 (2) A	0.4 ± 0.3 (3) A	1.1 ± 0.6 (9) A	4.4 ± 1.5 (29) A	0.7 ± 0.4 (5) A	89.2 ± 3.9 (641) A
25	Normoxia	6	83.3 ± 12.7 (605) A	0.4 ± 0.3 (4) A	0.7 ± 0.4 (3) A	6.4 ± 5.7 (17) A	5.6 ± 4.4 (18) A	0.4 ± 0.4 (1) A	3.2 ± 1.9 (14) A
25	Hypoxia	6	78.2 ± 5.6 (1323) A	0.6 ± 0.3 (11) A	4.1 ± 1.8 (48) B	12.1 ± 3.6 (160) A	1.3 ± 0.2 (23) B	0.6 ± 0.2 (11) A	3.1 ± 1.4 (74) A
25	Anoxia	6	28.9 ± 15.3 (116) B	0.9 ± 0.7 (2) A	4.1 ± 2.1 (11) B	22.7 ± 6.0 (93) B	13.6 ± 3.8 (79) C	11.9 ± 9.3 (64) B	17.8 ± 7.5 (106) B
35	Normoxia	6	98.6 ± 0.9 (834) A	0.0 ± 0.0 (0) A	0.2 ± 0.2 (2) A	1.1 ± 0.7 (10) A	0.04 ± 0.04 (1) A	0.0 ± 0.0 (0) A	0.0 ± 0.0 (0) A
35	Hypoxia	6	98.7 ± 0.9 (738) A	0.0 ± 0.0 (0) A	0.4 ± 0.4 (3) A	0.8 ± 0.6 (7) A	0.1 ± 0.1 (1) A	0.0 ± 0.0 (0) A	0.0 ± 0.0 (0) A
35	Anoxia	6	81.7 ± 8.2 (448) A	0.0 ± 0.0 (0) A	3.0 ± 2.1 (38) B	14.5 ± 6.2 (153) B	0.7 ± 0.6 (2) A	0.1 ± 0.1 (2) A	0.0 ± 0.0 (0) A
50	Normoxia	6	100.0 ± 0.0 (924) A	0.0 ± 0.0 (0) A	0.0 ± 0.0 (0) A	0.0 ± 0.0 (0) A	0.0 ± 0.0 (0) A	0.0 ± 0.0 (0) A	0.0 ± 0.0 (0) A
50	Hypoxia	6	100.0 ± 0.0 (1795) A	0.0 ± 0.0 (0) A	0.0 ± 0.0 (0) A	0.0 ± 0.0 (0) A	0.0 ± 0.0 (0) A	0.0 ± 0.0 (0) A	0.0 ± 0.0 (0) A
50	Anoxia	6	98.4 ± 1.6 (716) A	0.1 ± 0.1 (2) A	0.0 ± 0.0 (0) A	1.6 ± 1.6 (5) A	0.0 ± 0.0 (0) A	0.0 ± 0.0 (0) A	0.0 ± 0.0 (0) A
70	Normoxia	5	100.0 ± 0.0 (398) A	0.0 ± 0.0 (0) A	0.0 ± 0.0 (0) A	0.0 ± 0.0 (0) A	0.0 ± 0.0 (0) A	0.0 ± 0.0 (0) A	0.0 ± 0.0 (0) A
70	Hypoxia	6	100.0 ± 0.0 (1106) A	0.0 ± 0.0 (0) A	0.0 ± 0.0 (0) A	0.0 ± 0.0 (0) A	0.0 ± 0.0 (0) A	0.0 ± 0.0 (0) A	0.0 ± 0.0 (0) A
70	Anoxia	6	100.0 ± 0.0 (1009) A	0.0 ± 0.0 (0) A	0.0 ± 0.0 (0) A	0.0 ± 0.0 (0) A	0.0 ± 0.0 (0) A	0.0 ± 0.0 (0) A	0.0 ± 0.0 (0) A

[1] Normoxia (~21.0% O_2, 0.0% CO_2), hypoxia (5.5 ± 0.1% O_2, 15.7 ± 0.2% CO_2) and severe hypoxia (0.3 ± 0.02% O_2, 22.2 ± 0.2% CO_2). [2] CAL (coarctate larvae), CCP (cryptocephalic pupae), PCP (phanerocephalic pupae), PHA (pharate adults), PEA (partially emerged adults), DFA (deformed adults), FFA (fully formed adults). [3] Different letters indicate significant differences between groups (estimated marginal means contrasts, $P < 0.05$).

Table S9. Percentages [mean ± SE (total number of individuals)] of dead insects dissected from puparia (CAL, CCP, PCP, PHA, PEA) and emerged adults (DFA, FFA) of *Bactrocera dorsalis* after irradiation of third instars at different nominal doses and atmospheric conditions

Dose (Gy)	Atmospheric conditions[1]	n	CAL[2, 3]	CCP [2, 3]	PCP [2, 3]	PHA [2, 3]	PEA[2, 3]	DFA[2, 3]	FFA[2, 3]
0	Normoxia	5	5.3 ± 1.4 (74) A	0.0 ± 0.0 (0) A	0.5 ± 0.5 (3) A	1.8 ± 0.8 (38) A	0.4 ± 0.3 (10) A	1.0 ± 0.6 (12) AB	90.9 ± 2.1 (960) A
0	Hypoxia	3	3.3 ± 1.6 (8) A	0.52 ± 0.52 (1) A	0.0 ± 0.0 (0) A	0.3 ± 0.3 (1) A	0.3 ± 0.3 (4) A	0.6 ± 0.6 (6) A	94.9 ± 1.5 (330) A
0	Severe hypoxia	4	5.9 ± 1.9 (30) A	0.1 ± 0.1 (1) A	0.7 ± 0.7 (3) A	1.6 ± 0.9 (9) A	0.0 ± 0.0 (0) A	2.4 ± 1.7 (6) B	89.4 ± 2.6 (506) A
30	Normoxia	3	81.6 ± 7.2 (375) A	0.6 ± 0.3 (3) A	2.6 ± 1.3 (6) A	8.2 ± 3.5 (34) A	0.6 ± 0.3 (3) A	0.4 ± 2.6 (1) A	6.0 ± 3.3 (23) A
30	Hypoxia	3	69.5 ± 9.3 (606) A	1.66 ± 1.02 (12) A	2.0 ± 0.7 (23) A	17.5 ± 5.1 (197) AB	2.0 ± 0.8 (20) A	2.1 ± 1.2 (18) B	5.2 ± 2.4 (44) A
30	Severe hypoxia	3	13.2 ± 3.9 (76) B	1.0 ± 0.4 (8) A	3.3 ± 0.9 (22) A	26.2 ± 2.7 (223) B	6.5 ± 3.0 (82) B	13.9 ± 4.7 (94) C	35.7 ± 9.3 (351) B
40	Normoxia	3	72.7 ± 14.7 (527) A	0.4 ± 0.4 (1) A	5.2 ± 2.8 (18) A	17.5 ± 10.2 (55) A	0.9 ± 0.4 (3) A	2.2 ± 1.4 (6) A	1.0 ± 0.7 (6) A
40	Hypoxia	3	62.4 ± 16.8 (419) AB	0.0 ± 0.0 (0) A	0.4 ± 0.4 (1) B	19.9 ± 8.3 (30) A	0.0 ± 0.0 (0) A	9.9 ± 6.4 (5) B	7.4 ± 3.4 (8) AB
40	Severe hypoxia	3	38.5 ± 18.3 (48) B	10.6 ± 9.9 (3) B	1.7 ± 1.7 (6) B	25.7 ± 11.4 (54) A	1.8 ± 1.2 (6) A	2.3 ± 1.5 (8) A	19.3 ± 9.0 (54) B
80	Normoxia	3	99.9 ± 0.1 (942) A	0.0 ± 0.0 (0) A	0.0 ± 0.0 (0) A	0.0 ± 0.0 (0) A	0.0 ± 0.0 (0) A	0.0 ± 0.0 (0) A	0.05 ± 0.05 (1) A
80	Hypoxia	3	100.0 ± 0.0 (365) A	0.0 ± 0.0 (0) A	0.0 ± 0.0 (0) A	0.0 ± 0.0 (0) A	0.0 ± 0.0 (0) A	0.0 ± 0.0 (0) A	0.0 ± 0.0 (0) A
80	Severe hypoxia	3	99.2 ± 0.6 (506) A	0.0 ± 0.0 (0) A	0.0 ± 0.0 (0) A	0.4 ± 0.3 (2) A	0.4 ± 0.3 (2) A	0.0 ± 0.0 (0) A	0.0 ± 0.0 (0) A
116	Normoxia	3	100.0 ± 0.0 (561) A	0.0 ± 0.0 (0) A	0.0 ± 0.0 (0) A	0.0 ± 0.0 (0) A	0.0 ± 0.0 (0) A	0.0 ± 0.0 (0) A	0.00 ± 0.00 (0) A
116	Hypoxia	3	100.0 ± 0.0 (142) A	0.0 ± 0.0 (0) A	0.0 ± 0.0 (0) A	0.0 ± 0.0 (0) A	0.0 ± 0.0 (0) A	0.0 ± 0.0 (0) A	0.0 ± 0.0 (0) A
116	Severe hypoxia	4	100.0 ± 0.0 (650) A	0.0 ± 0.0 (0) A	0.0 ± 0.0 (0) A	0.0 ± 0.0 (0) A	0.0 ± 0.0 (0) A	0.0 ± 0.0 (0) A	0.0 ± 0.0 (0) A

[1] Normoxia (~21.0% O_2, 0.0% CO_2), hypoxia (5.3 ± 0.04% O_2, 15.0 ± 0.1% CO_2) and severe hypoxia (0.3 ± 0.02% O_2, 21.6 ± 0.1% CO_2). [2] CAL (coarctate larvae), CCP (cryptocephalic pupae), PCP (phanerocephalic pupae), PHA (pharate adults), PEA (partially emerged adults), DFA (deformed adults), FFA (fully formed adults). [3] Different letters indicate significant differences between groups (estimated marginal means contrasts, $P < 0.05$).

Table S10. Percentages [mean ± SE (total number of individuals)] of dead insects dissected from puparia (CAL, CCP, PCP, PHA, PEA) and emerged adults (DFA, FFA) of *Ceratitis capitata* after irradiation of third instars at different nominal doses and atmospheric conditions

Dose (Gy)	Atmospheric conditions[1]	n	CAL[2, 3]	CCP [2, 3]	PCP [2, 3]	PHA [2, 3]	PEA[2, 3]	DFA[2, 3]	FFA[2, 3]
0	Normoxia	3	2.9 ± 0.8 (10) A	1.0 ± 0.9 (2) A	1.4 ± 0.6 (6) A	4.3 ± 1.4 (11) A	0.8 ± 0.5 (3) A	0.3 ± 0.3 (2) A	89.3 ± 1.7 (255) A
0	Hypoxia	3	2.9 ± 1.0 (16) A	0.2 ± 0.2 (1) A	2.3 ± 1.4 (8) A	6.1 ± 2.7 (19) A	1.3 ± 0.7 (5) A	0.7 ± 0.6 (5) A	86.4 ± 3.0 (358) A
0	Severe hypoxia	3	10.9 ± 5.3 (5) A	0.0 ± 0.0 (0) A	5.9 ± 4.2 (11) A	6.9 ± 4.1 (6) A	2.6 ± 2.4 (2) A	0.2 ± 0.2 (1) A	73.5 ± 11.3 (102) B
20	Normoxia	3	43.4 ± 10.8 (129) A	0.0 ± 0.0 (0) A	3.9 ± 1.6 (14) A	24.3 ± 6.2 (68) A	3.3 ± 2.4 (14) A	1.3 ± 1.1 (3) A	23.8 ± 15.7 (14) A
20	Hypoxia	3	26.2 ± 5.6 (50) B	3.3 ± 1.6 (5) B	2.4 ± 1.1 (5) A	37.5 ± 6.6 (73) B	2.3 ± 1.4 (7) A	2.7 ± 1.6 (4) A	25.8 ± 6.9 (37) A
20	Severe hypoxia	3	6.7 ± 2.9 (7) C	0.0 ± 0.0 (0) A	10.9 ± 4.2 (11) B	19.2 ± 8.7 (14) A	4.7 ± 4.1 (3) B	0.9 ± 0.6 (3) A	57.5 ± 12.4 (99) B
30	Normoxia	3	93.5 ± 5.0 (382) A	0.6 ± 0.6 (2) A	0.6 ± 0.6 (1) A	3.2 ± 2.3 (16) A	0.7 ± 0.6 (2) A	0.1 ± 0.1 (1) A	1.4 ± 1.1 (4) A
30	Hypoxia	3	47.9 ± 11.7 (140) B	17.5 ± 16.5 (4) B	1.1 ± 0.6 (4) A	22.4 ± 5.5 (63) B	0.1 ± 0.1 (1) A	0.4 ± 0.4 (4) A	10.5 ± 5.8 (41) B
30	Severe hypoxia	3	15.4 ± 4.9 (17) C	2.8 ± 1.8 (4) C	9.7 ± 3.8 (12) B	43.6 ± 10.1 (32) C	0.0 ± 0.0 (0) A	3.1 ± 1.9 (3) B	25.4 ± 6.3 (28) C
50	Normoxia	3	100.0 ± 0.0 (442) A	0.0 ± 0.0 (0) A	0.0 ± 0.0 (0) A	0.0 ± 0.0 (0) A	0.0 ± 0.0 (0) A	0.0 ± 0.0 (0) A	0.0 ± 0.0 (0) A
50	Hypoxia	3	98.4 ± 1.6 (118) A	0.0 ± 0.0 (0) A	0.8 ± 0.8 (1) A	0.7 ± 0.7 (1) A	0.0 ± 0.0 (0) A	0.0 ± 0.0 (0) A	0.0 ± 0.0 (0) A
50	Severe hypoxia	3	89.5 ± 5.9 (118) A	3.7 ± 3.7 (4) B	0.2 ± 0.2 (1) A	6.5 ± 2.7 (11) B	0.0 ± 0.0 (0) A	0.0 ± 0.0 (0) A	0.0 ± 0.0 (0) A
70	Normoxia	3	100.0 ± 0.0 (283) A	0.0 ± 0.0 (0) A	0.0 ± 0.0 (0) A	0.0 ± 0.0 (0) A	0.0 ± 0.0 (0) A	0.0 ± 0.0 (0) A	0.0 ± 0.0 (0) A
70	Hypoxia	3	99.5 ± 0.5 (161) A	0.0 ± 0.0 (0) A	0.0 ± 0.0 (0) A	0.5 ± 0.5 (1) A	0.0 ± 0.0 (0) A	0.0 ± 0.0 (0) A	0.0 ± 0.0 (0) A
70	Severe hypoxia	3	98.6 ± 0.6 (316) A	0.0 ± 0.0 (0) A	1.0 ± 0.6 (3) A	0.4 ± 0.4 (1) A	0.0 ± 0.0 (0) A	0.0 ± 0.0 (0) A	0.0 ± 0.0 (0) A
100	Normoxia	4	100.0 ± 0.0 (536) A	0.0 ± 0.0 (0) A	0.0 ± 0.0 (0) A	0.0 ± 0.0 (0) A	0.0 ± 0.0 (0) A	0.0 ± 0.0 (0) A	0.0 ± 0.0 (0) A
100	Hypoxia	3	100.0 ± 0.0 (440) A	0.0 ± 0.0 (0) A	0.0 ± 0.0 (0) A	0.0 ± 0.0 (0) A	0.0 ± 0.0 (0) A	0.0 ± 0.0 (0) A	0.0 ± 0.0 (0) A
100	Severe hypoxia	3	100.0 ± 0.0 (149) A	0.0 ± 0.0 (0) A	0.0 ± 0.0 (0) A	0.0 ± 0.0 (0) A	0.0 ± 0.0 (0) A	0.0 ± 0.0 (0) A	0.0 ± 0.0 (0) A

[1] Normoxia (~21.0% O_2, 0.0% CO_2), hypoxia (5.2 ± 0.05% O_2, 15.6 ± 0.1% CO_2) and severe hypoxia (0.4 ± 0.03% O_2, 21.5 ± 0.1% CO_2). [2] CAL (coarctate larvae), CCP (cryptocephalic pupae), PCP (phanerocephalic pupae), PHA (pharate adults), PEA (partially emerged adults), DFA (deformed adults), FFA (fully formed adults). [3] Different letters indicate significant differences between groups (estimated marginal means contrasts, $P < 0.05$)
